# Substituted 1,2,4-Triazoles as Novel and Selective Inhibitors of Leukotriene Biosynthesis Targeting 5-Lipoxygenase-Activating Protein

**DOI:** 10.1021/acsomega.3c03682

**Published:** 2023-08-18

**Authors:** Abdurrahman Olğaç, İrfan Çapan, Philipp Dahlke, Paul M. Jordan, Oliver Werz, Erden Banoglu

**Affiliations:** † Department of Pharmaceutical Chemistry, Faculty of Pharmacy, Gazi University, Yenimahalle 06560 ,Ankara ,Turkey; ‡ Department of Drug Discovery, Evias Pharmaceutical R&D Ltd., Yenimahalle06830 ,Ankara ,Turkey; § Department of Material and Material Processing Technologies Technical Sciences Vocational College, Gazi University, Yenimahalle06374 ,Ankara ,Turkey; ∥ Department of Pharmaceutical/Medicinal Chemistry, Institute of Pharmacy, Friedrich Schiller University Jena, Philosophenweg 14, D-7743 Jena, Germany

## Abstract

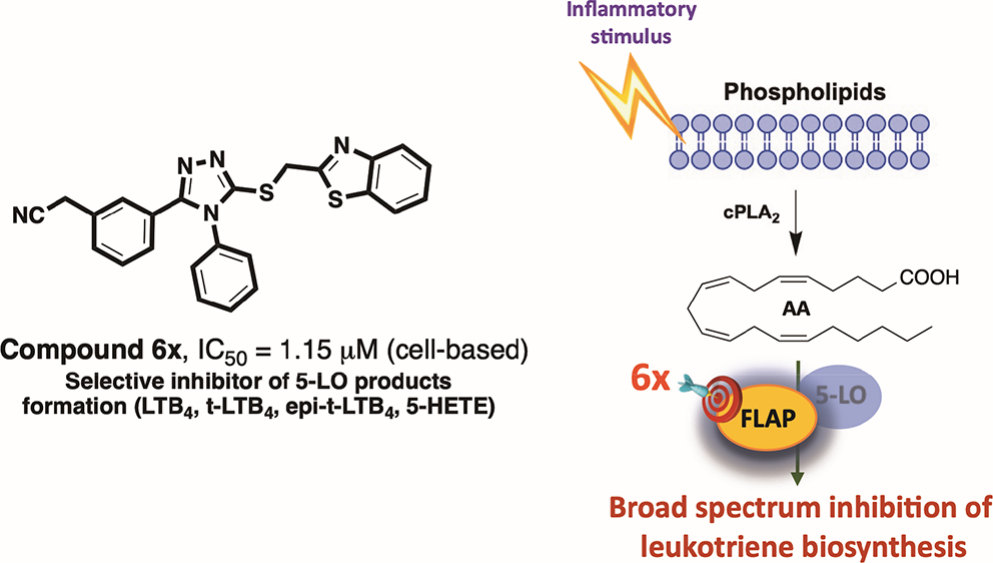

5-Lipoxygenase-activating protein (FLAP) is a regulator of cellular leukotriene biosynthesis, which governs the transfer of arachidonic acid (AA) to 5-lipoxygenase for efficient metabolism. Here, the synthesis and FLAP-antagonistic potential of fast synthetically accessible 1,2,4-triazole derivatives based on a previously discovered virtual screening hit compound is described. Our findings reveal that simple structural variations on 4,5-diaryl moieties and the 3-thioether side chain of the 1,2,4-triazole scaffold markedly influence the inhibitory potential, highlighting the significant chemical features necessary for FLAP antagonism. Comprehensive metabololipidomics analysis in activated FLAP-expressing human innate immune cells and human whole blood showed that the most potent analogue **6x** selectively suppressed leukotriene B_4_ formation evoked by bacterial exotoxins without affecting other branches of the AA pathway. Taken together, the 1,2,4-triazole scaffold is a novel chemical platform for the development of more potent FLAP antagonists, which warrants further exploration for their potential as a new class of anti-inflammatory agents.

## 1 Introduction

**Figure 1:**
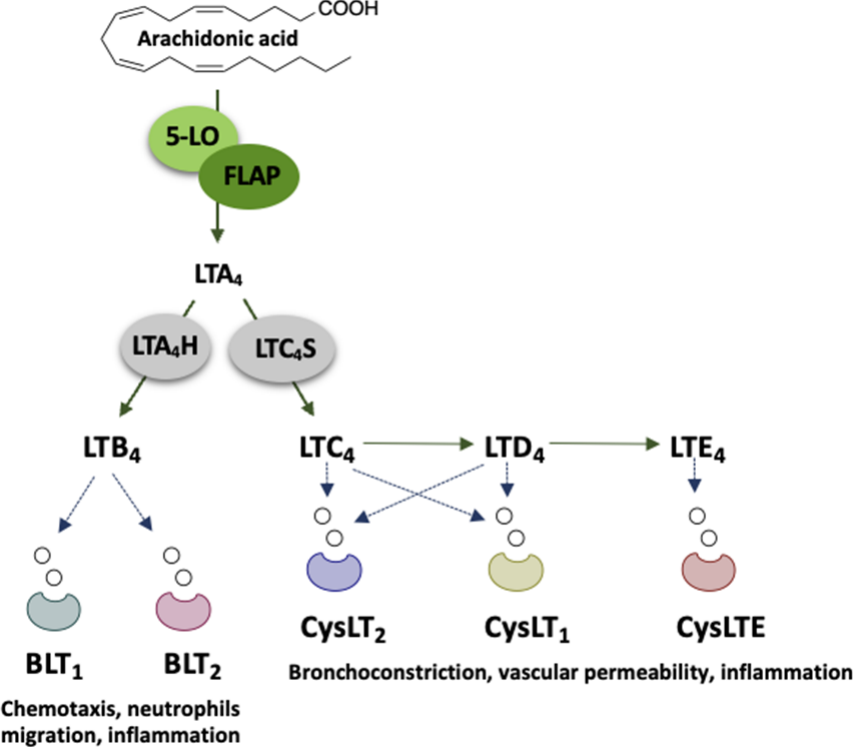
Schematic representation of the 5-LO branch of the AA pathway for the formation of LTs.

Leukotrienes (LTs) are a family of bioactive lipid mediators produced from arachidonic acid (AA) through the 5-lipoxygenase (5-LO) pathway, which have various inflammatory and vasoactive effects.^1,2^ The 5-LO branch of the AA cascade produces LTB_4_ and cysteinyl (Cys) LTs from an unstable LTA_4_ intermediate via the action of LTA_4_ hydrolase and LTC_4_ synthase, respectively. These bioactive LTs exert their actions via distinct receptors such as BL*T*_1/2_ for LTB_4_ and CysLTRs for CysLTs (Figure 1).^3^

LTB_4_ is pro-inflammatory and acts as a chemoattractant for leukocytes and neutrophils, while cysLTs cause bronchoconstriction, airway edema, and vascular leakage.^1^ To date, the most advanced drug class targeting this branch are cysLTR_1_ antagonists as antiasthmatic drugs, i.e., montelukast,^4^ but this drug class has limited clinical indication as it essentially blocks the action of LTD_4_ in the lungs, resulting in decreased inflammation and relaxation of smooth muscle.^5^ Meanwhile, the development of broad-spectrum inhibitors of LT biosynthesis (LTB_4_, C_4_, D_4_, and E_4_) is less progressed. Zileuton is the only clinical example of a direct 5-LO inhibitor for asthma treatment, but its use is often limited due to idiosyncratic hepatotoxicity and poor pharmacokinetics.^6^ In this regard, there remains an ever-increasing demand to search for novel anti-inflammatory drugs that target the LT pathway.

**Figure 2:**
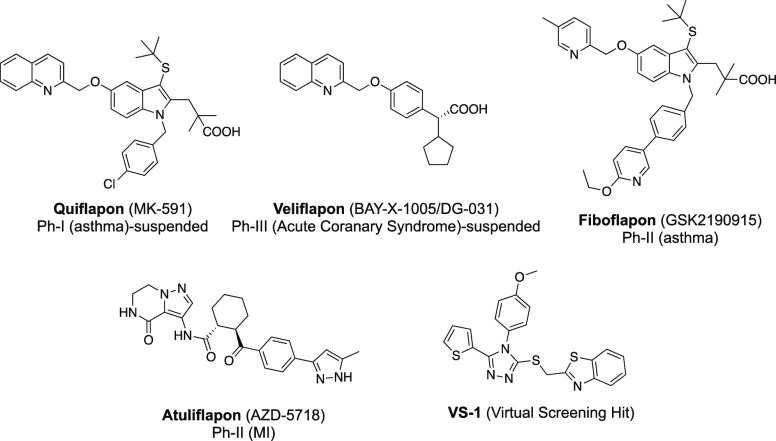
Chemical structures of FLAP antagonists that entered clinical trials and the virtual screening hit compound **VS-1**.

Over the past three decades, there has been great interest in 5-lipoxygenase activating protein (FLAP) as an essential partner of 5-LO, which transfers AA to 5-LO for efficient metabolism.^7^ FLAP is an indispensable regulatory protein for 5-LO without any enzymatic activity at the nuclear membrane to assemble the LT synthetic complex.^8^ Hence, it is envisaged that suppression of the FLAP function would selectively intervene with massive, pro-inflammatory, and vasoactive LT production to provide a therapeutic benefit for chronic inflammatory diseases besides asthma.^9,10^ Indeed, prevailing clinical data imply that FLAP antagonists exert potent and safer anti-LT efficacy in asthma and chronic artery disease, as exemplified by quiflapon,^11,12^ veliflapon,^13^ fiboflapon,^14,15^ and very recently atuliflapon^16,17^ (Figure 2). However, despite great interest in this biological target over the past few decades, no FLAP antagonist has so far reached clinical practice.^7,18^

**Figure 3:**
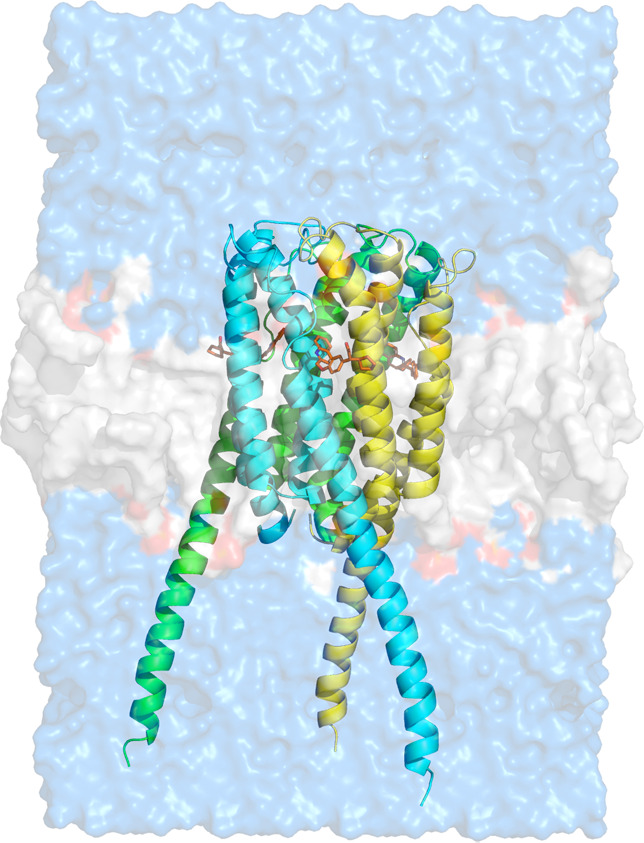
Structural overview of FLAP modeled in the nuclear membrane. The whole structure was generated by aligning the crystal structure with the best resolution (PDB code: 6VGC) on the predicted FLAP model by AlphaFold2.^28−30^ Missing residues on the nuclear region (bottom) and on the loops located at the cytosolic region (top) of the protein were added after a relaxation (energy minimization with an RMSD cutoff 0.3) by using homology modeling software Prime.^31−33^ Membrane structure and water molecules were added using the predicted membrane orientations from OPM server^34^ with the *System Builder* tool in Maestro.^35^

FLAP is a homotrimer located in the nuclear membrane comprising four transmembrane helices (α1−α4) in each monomer. Based on the reported FLAP crystal structures, ligands that bind FLAP are largely embedded in the nonpolar center of the trimer, which is located between the α2 and α4 of one FLAP monomer and the α1′ and α2′ of the other FLAP monomer (Figure 3).^19,20^ Moreover, polar parts of the ligands stick out to the phosphate-exposed solvent accessible protein surface, where the ligands can form salt-bridges/H-bonds with basic side chains such as Lys116 and His28; these, in turn, engage with the anionic phosphate groups of the plasma membrane on the side adjacent to the aqueous cytosol. These ligands have little exposure to the lipid bilayer when bound to FLAP.

Our groups have long been involved in the development of novel LT biosynthesis inhibitors targeting FLAP with distinct chemical scaffolds.^21,23−26^ Thus, as a result of a recent virtual screening approach for novel chemotypes that interfere with FLAP function, a 1,2,4-triazole derivative (**VS-1**, Figure 2) was identified as a novel FLAP antagonist that has a distinct scaffold compared to reported FLAP antagonists.^27^ To further elucidate the structural determinants of this class of compounds for FLAP antagonism, the current work reports the synthesis, biological evaluation, and analysis of structure–activity relationships (SAR) of novel 1,2,4-triazole analogs that could potentially provide further insight into drug development in the AA pathway targeting FLAP.

## 2 Results and Discussion

### 2.1 Chemistry

**Scheme 1:**
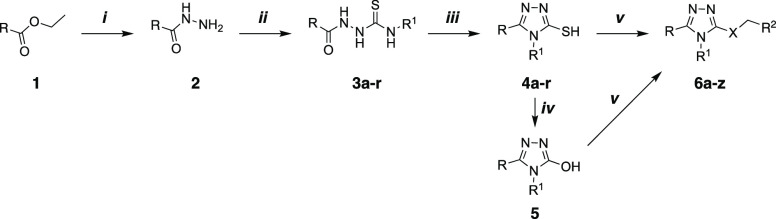
Reagents and Conditions: (i) NH_2_NH_2_•H_2_O, EtOH, Δ; (ii) isothiocyanate derivatives, EtOH, Δ; (iii) 2N NaOH or saturated NaHCO_3_, Δ; (iv) 50% H_2_O_2_, Δ; (v) alkyl halide, AcCN, Et_3_N, rt

To determine the effects of the substitution pattern on 4,5-diaryl subunits and the influence of the thioether side chain of the 1,2,4-triazole core on FLAP antagonism, compounds **6a** and its analogs (Table 1, **6b–z**) were synthesized by following the synthetic procedure demonstrated in Scheme 1. The general method is straightforward and utilizes the cyclodehydration of thiosemicarbazide intermediates (**3**), which were conveniently produced from corresponding acyl hydrazides (**2**), under basic conditions to form corresponding 3-mercapto-4,5-disubstituted-1,2,4-triazoles (**4**).^36,37^ For the synthesis of compounds with modified spacers by a formal exchange of the sulfur by oxygen, the 3-hydroxy-1,2,4-triazole intermediate **5** was directly prepared by heating **4** in a 50% hydrogen peroxide solution under basic conditions. The target compounds **6a–z** were finally generated by simple alkylation of intermediates **4** or **5** with the corresponding alkyl halides in acetonitrile in the presence of triethylamine. The final purity of the target compounds was corroborated by UPLC-MS prior to biological evaluation (purity was >97%). Compound structure elucidation was done through high-resolution mass spectrometry (HRMS) and ^1^H- and ^13^C NMR spectral data as given in the Supporting Information.

**Table 1:**
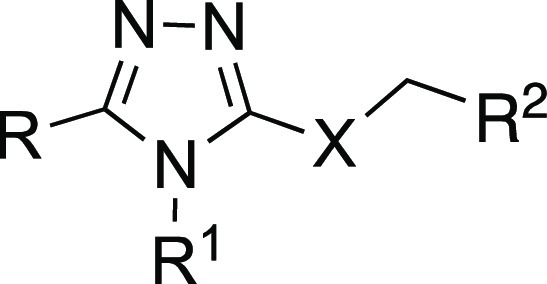
Inhibitory Effects of New 1,2,4-Triazole Analogs on 5-LO Product Synthesis in a Cell-based Assay using Intact Neutrophils^a^

**#**	**R**	**R**^**1**^	**X**	**R**^**2**^	**5-LO product formation in neutrophils**^a^, **remaining activity (% of control) at**		**IC**_**50**_**(μM)**
					**1 μM**	**10 μM**	
**6a**(VS1)	thiophen-2-yl	4-OMe-Ph	S	benzothiazol-2-yl	100.0 ± 6.8	6.0 ± 6.0	2.18 ± 0.65
**6b**	thiophen-2-yl	Me	S	benzothiazol-2-yl	79.9 ± 18.2	72.0 ± 35.7	>10
**6c**	thiophen-2-yl	3-OMe-Ph	S	benzothiazol-2-yl	72.8 ± 3.2	4.0 ± 1.6	1.58 ± 0.59
**6d**	thiophen-2-yl	4-Cl-Ph	S	benzothiazol-2-yl	68.1 ± 8.2	0.0 ± 0.0	1.36 ± 0.27
**6e**	thiophen-2-yl	4-COOH-Ph	S	benzothiazol-2-yl	93.0 ± 14.3	55.6 ± 10.3	∼10
**6f**	thiophen-2-yl	3-COOH-Ph	S	benzothiazol-2-yl	94.8 ± 2.0	81.2 ± 5.4	>10
**6g**	thiophen-2-yl	4-CH_2_COOH-Ph	S	benzothiazol-2-yl	93.8 ± 9.1	77.6 ± 13.4	>10
**6h**	thiophen-2-yl	4-CH_2_CH_2_COOH-Ph	S	benzothiazol-2-yl	102.8 ± 8.5	104.7 ± 3.6	>10
**6i**	thiophen-2-yl	4-NO_2_–Ph	S	benzothiazol-2-yl	95.5 ± 6.1	49.7 ± 11.8	∼10
**6j**	thiophen-2-yl	4-OMe-Ph	S	quinolin-2-yl	95.4 ± 21.6	21.1 ± 10.3	3.69 ± 1.02
**6k**	thiophen-2-yl	3-OMe-Ph	S	quinolin-2-yl	96.4 ± 5.0	20.7 ± 16.4	4.17 ± 0.27
**6l**	thiophen-2-yl	4-OMe-Ph	S	5-CF_3_-furan-2-yl	83.4 ± 6.8	1.0 ± 1.7	1.42 ± 0.31
**6m**	thiophen-2-yl	4-OMe-Ph	S	5-Me-pyridin-2-yl	97.7 ± 5.2	49.3 ± 10.7	∼10
**6n**	thiophen-2-yl	4-OMe-Ph	O	benzothiazol-2-yl	85.9 ± 5.3	0.6 ± 0.5	3.01 ± 0.84
**6o**	thiophen-2-yl	4-OMe-Ph	O	quinolin-2-yl	96.8 ± 3.9	37.7 ± 13.2	6.34 ± 0.79
**6p**	Me	4-OMe-Ph	S	benzothiazol-2-yl	94.7 ± 5.0	95.4 ± 19.6	>10
**6q**	Ph	4-OMe-Ph	S	benzothiazol-2-yl	57.9 ± 11.1	0.0 ± 0.0	1.28 ± 0.19
**6r**	4-Cl-Ph	4-OMe-Ph	S	benzothiazol-2-yl	61.6 ± 15.5	82.6 ± 8.8	>10
**6s**	4-CN-Ph	4-OMe-Ph	S	benzothiazol-2-yl	71.7 ± 11.1	-	1.92 ± 0.58
**6t**	4-CH_2_CN-Ph	4-OMe-Ph	S	benzothiazol-2-yl	72.8 ± 15.0	9.0 ± 5.0	3.62 ± 1.14
**6u**	4-CH_2_COOH-Ph	4-OMe-Ph	S	benzothiazol-2-yl	89.6 ± 13.5	60.8 ± 13.2	>10
**6v**	4-CH_2_CN-Ph	4-Cl-Ph	S	benzothiazol-2-yl	70.9 ± 21.8	0.0 ± 0.0	1.35 ± 0.07
**6w**	4-CH_2_CN-Ph	Ph	S	benzothiazol-2-yl	55.3 ± 17.9	0.0 ± 0.0	1.51 ± 0.24
**6x**	3-CH_2_CN-Ph	Ph	S	benzothiazol-2-yl	37.8 ± 13.6	0.2 ± 0.4	1.15 ± 0.21
**6y**	3-CH_2_CN-Ph	4-Cl-Ph	S	benzothiazol-2-yl	72.7 ± 3.2	4.7 ± 1.6	1.43 ± 0.27
**6z**	4-CN-Ph	Ph	S	benzothiazol-2-yl	52.6 ± 12.3	0.0 ± 0.0	1.93 ± 0.37

a Compounds were tested in human neutrophils stimulated with 2.5 μM A23187. Data are given as percentage of control at 1 and 10 μM inhibitor concentration (means ± SEM, *n* = 3). The FLAP antagonist 3-[3-(*tert*-butylthio)-1-(4-chlorobenzyl)-5-isopropyl-1*H*-indol-2-yl]-2,2-dimethyl propanoic acid (MK-886; IC_50_ = 0.08 μM) was used as a positive control.^25,38^

b The IC_50_ values are given as mean ± SEM of *n* = 3 determinations.

### 2.2 Biological Evaluation and SAR

Although FLAP is dispensable for 5-LO activity under cell-free conditions, it is essential for cellular LT biosynthesis, and FLAP antagonists do not have inhibitory activity toward 5-LO in cell homogenates.^39,40^ Hence, for determination of FLAP antagonism, we applied a well-documented “FLAP-dependent” cell-based assay for suppression of 5-LO product (5-H(p)ETE, LTB_4_ and its all-trans isomers) formation using human neutrophils stimulated by Ca^2+^-ionophore A23817.^25^ In addition, a “FLAP-independent” cell-free 5-LO activity assay using isolated recombinant 5-LO was employed to rule out direct inhibitory effects against 5-LO.

We previously identified the **VS-1** (**6a** in Table 1, IC_50_ = 2.18 μM) having a 1,2,4-triazole skeleton as a new chemotype for blocking FLAP function,^27^ which lacks typical scaffolds of reported FLAP antagonists.^7,18^ To deduce SARs around the 1,2,4-triazole skeleton, we randomly prepared analogs of **VS-1** (**6a**), which incorporate changes that would help in elucidating the structural features required to inhibit FLAP function (Table 1). First, we investigated the influence of the 4-methoxyphenyl moiety on the 4-position of the triazole ring (Table 1). Removal of the 4-methoxyphenyl group of **6a** resulted in a complete loss of FLAP function (**6b**, IC_50_ = > 10 μM). Therefore, we subsequently examined the effect of the substituent on the phenyl group attached to the 4-position of the triazole ring by casual introduction of different substituents. Moving the methoxy group to the meta position (**6c**, IC_50_ = 1.58 μM) or replacing it with a chloro group (**6d**, IC_50_ = 1.36 μM) was beneficial to enhance the inhibitory potency against 5-LO product formation. Next, SAR was further explored by replacing the methoxy group with polar carboxyl or nitro groups (**6e–i**), which all abolished the inhibitory activity. This indicated that a phenyl group with rather hydrophobic substituents at the 4-position of the triazole ring is important for FLAP antagonistic activity. It is known that the quinoline ring occurs as a frequently recurring chemical fragment in the architecture of FLAP antagonists (see Figure 2), and simple heteroaryl modifications may also modulate the inhibitory activity of FLAP function.^7,38^ Thus, we briefly explored the quinoline (**6j–k**, IC_50_ = 3.69–4.17 μM), 5- CF_3_-furan-2-yl (**6l**, IC_50_ = 1.42 μM), and 5-Me-pyridin-2-yl (**6m**, IC_50_ ∼ 10 μM) rings as replacements of the benzothiazole ring, which clearly indicated that the antagonistic activity of FLAP is sensitive to the nature of the heteroaryl fragment connected to the triazole via a thio-methyl linker at the 3-position. In addition, we briefly examined the thioether spacer by replacing it with an ether, which also caused a decrease in inhibitory activity (**6n–o**, IC_50_ = 3.0–6.3 μM).

Having elucidated the necessity of the 4-methoxyphenyl ring and the thio-methyl-benzothiazole side arm, we then investigated the effect of the hydrophobic thiophene ring on the 5-position of the 1,2,4-triazole core. While complete removal of the thiophene ring impairs the inhibitory potency (**6p**, IC_50_ = > 10 μM), the activity was restored by reinstallation of an aromatic phenyl ring (**6q**, IC_50_ = 1.28 μM). These results directed us further to conduct SAR studies around the 5-phenyl of the triazole, while keeping the other parts of the molecule intact. While the 4-chlorophenyl group at this position failed to substantially inhibit 5-LO product formation (**6r**, ∼ 20% inhibition at 10 μM), the activity was refurbished by replacing the 4-chloro with the more polar 4-nitrile substituent (**6s**, IC_50_ = 1.92 μM) that is an H-bond acceptor group with a rodlike geometry and a minuscule steric demand.^41^ Homologation of the nitrile in **6s** to acetonitrile gave **6t**, although with a decreased potency (IC_50_ = 3.62 μM). In addition, replacing the nitrile group with an H-bond acceptor carboxyl group in **6u** diminished the inhibitory potency (40% inhibition at 10 μM). Moreover, while keeping the 4-acetonitrile group at 5-phenyl of the triazole intact, we introduced the 4-chlorophenyl or phenyl groups to the 4-position of the triazole core, which resulted in more active compounds (**6v–w**, IC_50_ = 1.35–1.51 μM). Lastly, moving the acetonitrile substituent of **6w** to the 3-position at **6x**, both with a phenyl group at the 4-position of the triazole core, further enhanced the inhibitory activity (IC_50_ = 1.15 μM), leading to the most potent derivative in the series. Therefore, we selected **6x** for comprehensive analysis of its potency toward 5-LO product formation and its selectivity among various enzymes in the AA cascade, as well as its effects on de novo-biosynthesized lipid mediators that critically regulate both inflammation and resolution, by employing targeted liquid chromatography–tandem mass spectrometry-based metabololipidomics.^42^

### 2.3 **6x** Modulates Lipid Mediator Signature Profiles in Activated 5-LO-Rich Immune Cells and in Human Blood

**Figure 4:**
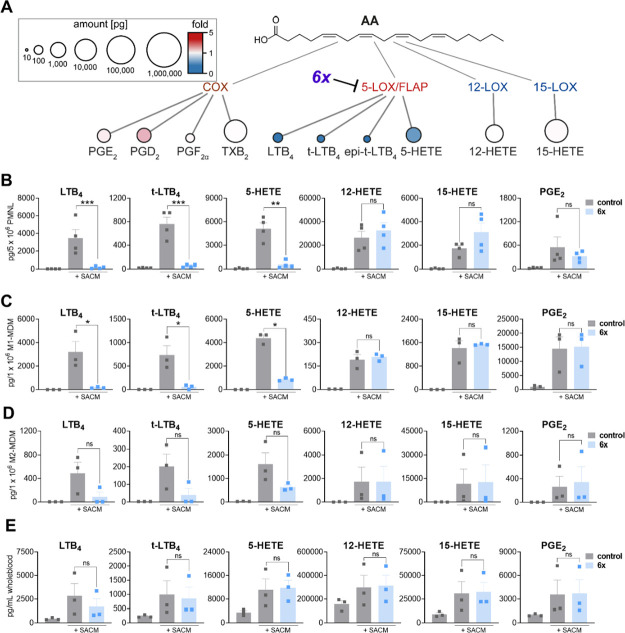
Modulation of lipid mediator profiles in activated 5-LO-rich immune cells and whole blood by **6x**. (A) Quantitative LM pathway analysis and effects of **6x** in exotoxin-stimulated M2-MDM. Node size represents the mean values in pg/2 × 10^6^ cells, and intensity of color denotes the fold change of **6x**- versus vehicle-treated cells for each LM; *n* = 3. Effects of **6x** on the bioactive LTs and PGE_2_ produced in human neutrophils (B), M1-MDM (C), M2-MDM (D), and freshly drawn human whole blood (E). Statistical analysis was done by matched one-way ANOVA with Tukey's multiple comparison test, *p < 0.05, **p < 0.01, ***p < 0.001, **6x** vs control.

Previous results showed that FLAP antagonists, such as MK886, can effectively inhibit LT formation but also affect other branches in the lipid mediator (LM) network, namely, 12/15-LO or COX-1/2 pathways.^42^ To investigate the potential redirection of LM formation due to FLAP antagonism, we studied the impact of **6x** on modulation of LM profiles of relevant cells by performing comprehensive LM metabololipidomics using UPLC-MS-MS (Figure 4).

5-LO-expressing innate immune cells, namely, neutrophils, M1- and M2-monocyte-derived macrophages (MDMs), which also possess various other LOs and COX enzymes,^43^ were pretreated with **6x** (3 μM) for 30 min before exposing to *Staphylococcus aureus*-conditioned medium (SACM, 1%) for 90 min, which contains bacterial exotoxins that are suitable stimuli to activate all relevant LM pathways in these cells.^44^ Formed LM derived from AA in **6x**-treated M2-MDM are shown by quantitative LM pathway analysis, revealing potent suppression of all 5-LO/FLAP-mediated LMs, while others such as COX and 12/15-LO products remained unaffected (Figure 4A). Notably, we observed cell type-specific inhibition of 5-LO/FLAP-mediated products such as LTB_4_, t-LTB_4,_ and 5-HETE. Thus, in neutrophils and M1-MDM cells expressing abundant FLAP, **6x** potently inhibited 5-LO/FLAP product formation (Figure 4B,C and Table 2), while in M2-MDMs, the efficiency of **6x** was reduced in accordance with minor FLAP expression in this MDM phenotype^42^ (Figure 4D and Table 2). Interestingly, the potency to inhibit proinflammatory LTB_4_ was higher than the suppression of 5-HETE formation in both M1- and M2-MDMs (Table 2). Moreover, **6x** suppressed EPA-derived 5-LO/FLAP products such as 5-HEPE effectively, whereas DHA-derived 7-HDHA was less reduced (Table 2). In all investigated cell types, we found that **6x** did not affect other prominent LM such as 12-HETE, 15-HETE, or PGE_2_, representing 12-LO, 15-LO, and COX pathways, respectively (Figure 4B–D and Table 2). Besides inhibition in isolated cells, we also investigated the ability of **6x** to suppress 5-LO/FLAP-mediated LT formation in human whole blood. Therefore, we pretreated freshly withdrawn human blood with **6x** (30 μM) for 30 min prior to stimulation with SACM (3%) for 90 min. **6x** decreased LTB_4_ levels by around 50%, while other 5-LO/FLAP-, 12/15-LO-, or COX-mediated products are essentially unchanged, suggesting a lower potency of **6x** as a free drug in human whole blood (Figure 4E and Table 2). Concerning the selectivity of **6x** toward FLAP over 5-LO, **6x** did not inhibit 5-LO product formation in cell-free assays using isolated human recombinant 5-LO, whereas the direct 5-LO inhibitor zileuton used as a reference showed potency in this respect (Figure S1).

**Table 2:**
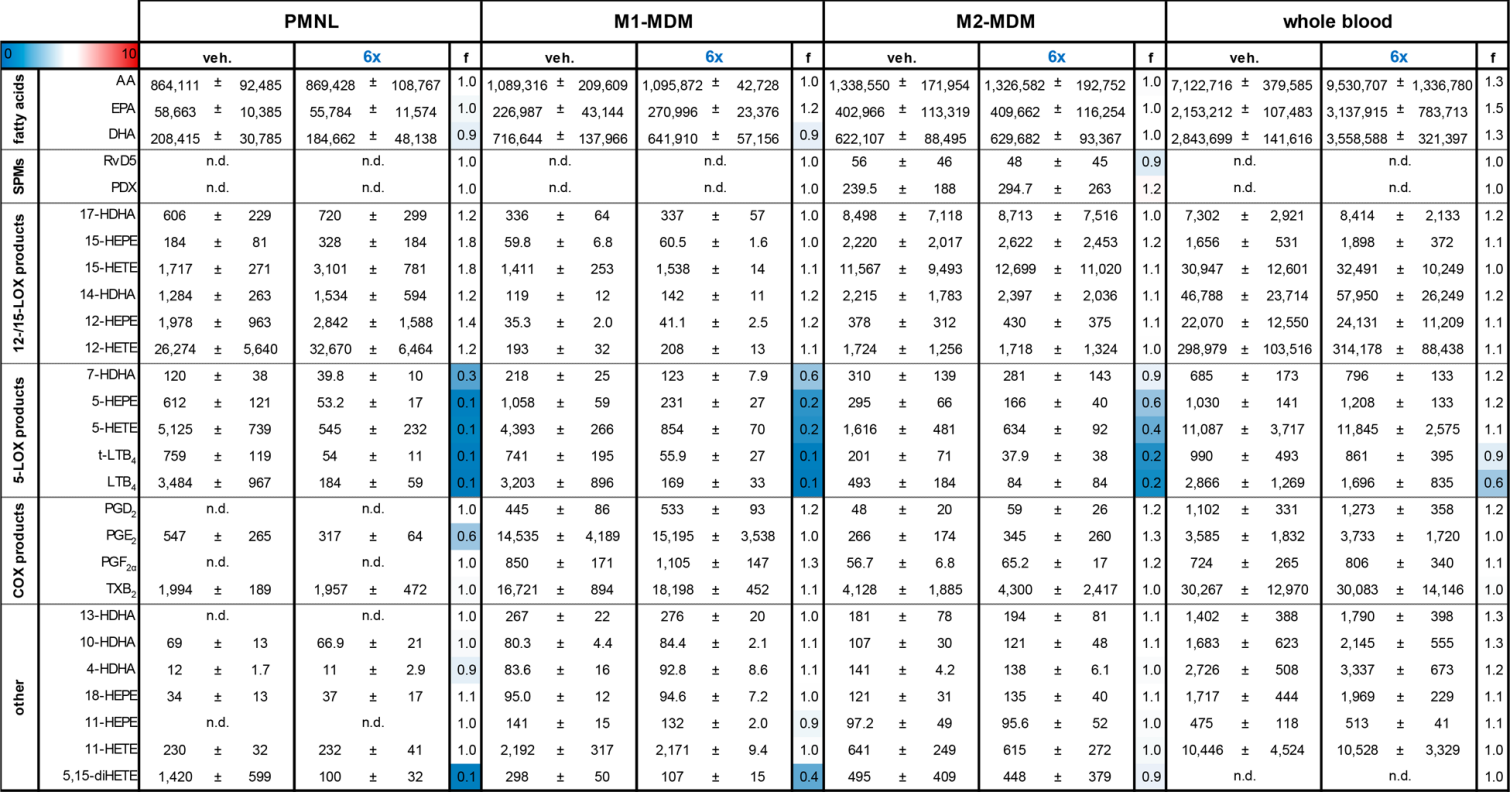
Modulation of Lipid Mediator Profiles in Activated 5-LO-Rich Immune Cells and Whole Blood by **6x**^a^

a Human neutrophils (PMNL), M1-MDM, and M2-MDM were preincubated with 3 μM **6x** or vehicle (0.1% DMSO) for 30 min before stimulation with 1% SACM for 90 min at 37 °C. Freshly drawn human whole blood was preincubated with 30 μM **6x** or vehicle (0.1% DMSO) for 30 min before stimulation with 1% SACM for 90 min at 37 °C. Formed LM were isolated from the supernatants by SPE and analyzed by UPLC-MS-MS. Data are given as mean ± SEM and as fold-change (‘f’) versus vehicle.

Considering the potential cytotoxicity, **6x** did not affect the membrane integrity after treatment of M1-MDMs for 90 min (Figure S2). These results indicate that **6x** indeed exerts its inhibitory effect on 5-LO product formation by selectively targeting FLAP.

### 2.4 Molecular Docking and Dynamics Simulations of **6x** with FLAP

**Figure 5:**
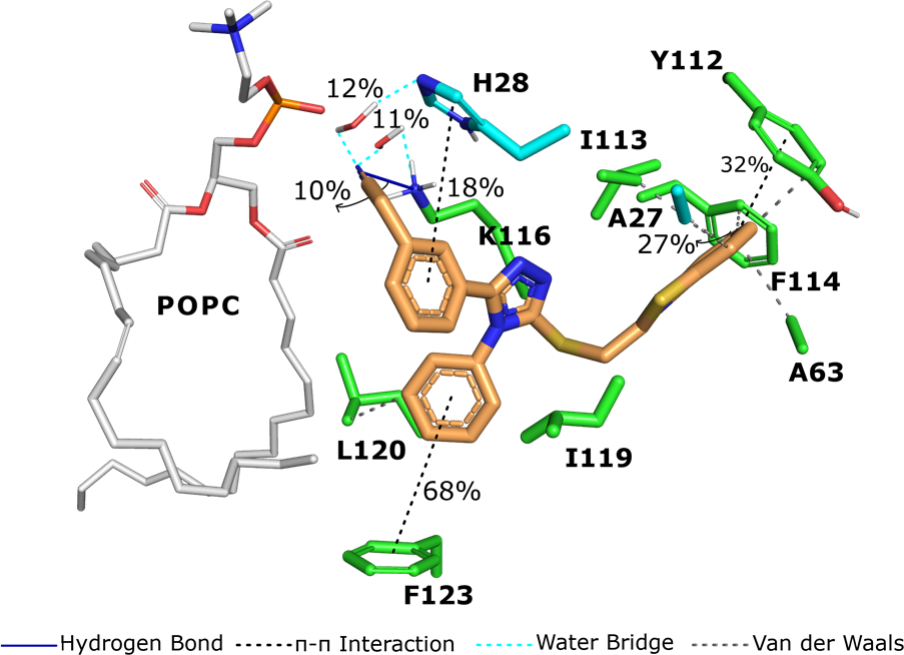
Protein–ligand interactions and their occupancy values obtained by 200 ns of molecular dynamic simulations conducted with FLAP-**6x** complex. Residues in green sticks represent the A chain, and cyan sticks represent the B chain. Compound **6x** is shown in orange sticks. POPC unit of the membrane is shown as white sticks. Interaction types and their representations are shown in the legend.

To provide further information on the interaction of **6x** with FLAP, we conducted docking studies in combination with molecular dynamic (MD) simulations using the recently reported FLAP crystal structure (2.35 Å, PDB code: 6VGC).^20^ The ligand binding site of FLAP is situated in the phosphate-exposed region of the protein, and ligands that bind FLAP largely embed themselves in the nonpolar center of the trimer between the α helices of the two adjacent monomers.^20^ In this respect, the docking**-**derived FLAP-**6x** complex (Figure S3) was submitted to 200 ns long MD simulations (four replicates, Figure S4A) after embedding the starting complex within the membrane (taken from OPM Database^34^). Considering the docking/MD calculations previously reported for FLAP-ligand complexes^21,27,45^ and shown in this study, the binding specificity of **6x** to FLAP is characterized by several interactions (Figure 5). Simulation analysis revealed that the existing interactions of **6x** from docking modes were generally conserved during MD runs (Figure S3B). Accordingly, the nitrile group of **6x** makes direct (10%) as well as water-mediated hydrogen bond interactions with the side chain of Lys116 (11%) and His28 (12%) at the phosphate-exposed binding region. In addition, the neighboring phenyl groups of **6x** engage in π–π stacking interactions with the imidazole ring of His28 (18%) and Phe123 (68%), which further stabilizes **6x** at the binding site. Moreover, the side chain benzothiazole group of **6x** tucks into a deep hydrophobic pocket with surrounding residues such as Ala27, Ala63, Tyr112, Ile113, Phe114, and Lue120 and makes π–π interactions with the aromatic residues of Tyr112 and Phe114 (32 and 27%, respectively). It seems that aromatic and hydrophobic interactions between **6x** and the nonpolar binding area of the FLAP binding site appear to be the main driving force for ligand binding, while the polar nitrile pendant presumably contributes little toward potency. Since potent FLAP inhibitors such as MK-591 are known to form strong polar interactions with Lys116 and His28 residues at the highly charged phosphate-exposed region of the binding site, the moderate activity of **6x** is likely due to the inability to bind this area effectively. Consequently, more favorable substitutions of the phenyl ring at **6x** could be a valuable tool for developing new analogs with greater potency, and further simulation analysis revealed that the existing interactions of **6x** from docking modes were generally conserved during MD runs (Figure S3 and S4).

## 3 Conclusions

**Figure 6:**
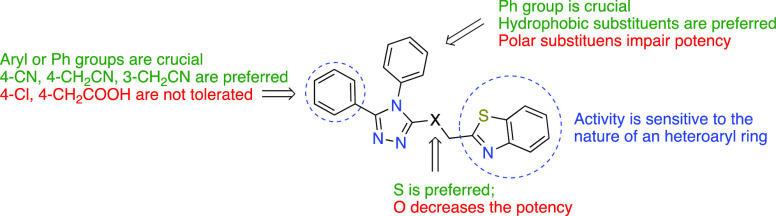
Brief SAR summary of 1,2,4-triazole derivatives for FLAP antagonism.

Through a methodical process of synthesis and biological assessment of **VS**-**1** analogs, we successfully established that the rapidly accessible 3,4,5-trisubstituted-1,2,4-triazole core is a new and essential framework for this novel class of FLAP antagonists, without any direct inhibitory effect on 5-LO activity. Our SAR results showed that the substitution pattern on either of the vicinal diaryl groups as well as the nature of the heterocyclic ring at the thioether side chain significantly influence the potency of compounds (Figure 6), and future explorations are needed to determine a more appropriate substitution pattern around these features to improve the potency of the compounds by increasing favorable binding interactions at the FLAP binding site. In addition, using human neutrophils and macrophages as experimental models, we established that **6x** displayed selective suppression of 5-LO product formation due to the antagonism of FLAP, without interfering with other branches in the AA cascade such as COX, 12/15-LO, and CYP450.

Taken together, **6x** represents a novel lead structure of LT biosynthesis inhibitors with unprecedented selectivity in human neutrophils and macrophages activated under pathophysiologically relevant conditions. Thus, based on its unique pharmacologic profile, the most potent analog **6x** was identified as a viable tool compound for future studies.

In conclusion, our results prove the ability of the 1,2,4-triazole core as an attractive new platform in the rational design of potential anti-LT drugs and provide valuable insight into the chemical features functional for the design of future members of this new class of FLAP antagonists.

## 4 Experimental Section

### 4.1 Chemistry

The starting materials, reagents, and solvents were obtained from BLD Pharm (BLD Pharmatech Ltd., Shanghai, China), ABCR (abcr GmbH, Karlsruhe, Germany), and Merck Chemicals (Merck KGaA, Darmstadt, Germany). The reaction progress was monitored by TLC using Merck silica gel Aluminum TLC plates, silica gel coated with fluorescent indicator F_254_, and visualized under UV. The melting points were determined by an SMP50 model automatic melting point apparatus (Stuart, Staffordshire, ST15 OSA, UK). The purification of the compounds was carried out on RediSep silica gel columns (12 and 24 g) using the Combiflash Rf Automatic Flash Chromatography System (Teledyne-Isco, Lincoln, NE, USA) or Buchi Pure C-815 Automatic Flash Chromatography System with UV and ELSD detectors using prepacked Buchi EcoFlex and FlashPure silica gel columns (12, 24, 40 g). The purity of the compounds was confirmed by thin-layer chromatography and UPLC/MS-TOF analyses. The ^1^H and ^13^C_APT_ NMR spectra of the synthesized compounds were taken in DMSO-*d*_6_ on Bruker Avance Neo 500 MHz and Bruker DPX-400 MHz High-Performance Digital FT-NMR Spectrometers. All chemical shift values (δ) were recorded as ppm, and coupling constants were reported in Hertz (Hz). HRMS spectra of the compounds were obtained on a Waters LCT Premier XE UPLC/MS-TOF system (Waters Corporation) using an Aquity BEH C18 column (2.1 × 100 mm 1.7 μM, flow rate: 0.3 mL/min) as the stationary phase, and CH_3_CN:H_2_O (1% → 90%) containing 0.1% formic acid as the mobile phase. Synthetic methods and experimental data for all intermediate compounds can be found in the Supporting Information.

#### 4.1.1 Synthetic procedure for compounds 6a-z

To the solution of the appropriate 1,2,4-triazole intermediate, **4a–r, 5** (1 equiv) in acetonitrile (15 mL) were added triethylamine (1.5 equiv) and the appropriate aryl halide (2-(chloromethyl)benzo[*d*]thiazole, 2-(chloromethyl)quinoline, 2-(chloromethyl)-5-(trifluoromethyl) furan, 2-(chloromethyl)-5-methylpyridine) (1 equiv), respectively. The mixture was stirred at room temperature for 24 h. The solvent was removed, and the crude product was dissolved with 5 mL of methanol and poured into 100 mL of water. The precipitate was filtered off to give a crude solid, which was purified by automated-flash chromatography on silica gel eluting with a gradient of 0% → 60% EtOAc in *n*-hexane.

##### 4.1.1.1 2-(((4-(4-Methoxyphenyl)-5-(thiophen-2-yl)-4*H*-1,2,4-triazol-3-yl)thio)methyl)benzo[*d*]thiazole (**6a, VS-1**)

Yield 91%; mp 191–193 °C. ^1^H NMR (400 MHz, DMSO) δ 8.08 (dd, *J* = 7.6, 1.2 Hz, 1H), 7.96 (dd, *J* = 7.9, 1.2 Hz, 1H), 7.66 (dd, *J* = 5.1, 1.2 Hz, 1H), 7.52 (dd, *J* = 8.3, 7.2, 1H), 7.49–7.36 (m, 3H), 7.19–7.09 (m, 2H), 7.03 (dd, *J* = 5.1, 3.7 Hz, 1H), 6.82 (dd, *J* = 3.7, 1.2 Hz, 1H), 4.88 (s, 2H), 3.86 (s, 3H). ^13^C NMR (101 MHz, DMSO) δ: 168.44, 161.13, 152.89, 151.23, 151.15, 135.77, 129.90, 129.40, 128.25, 128.20, 127.70, 126.77, 125.89, 125.82, 122.97, 122.75, 115.75, 56.08, 34.52. HRMS (*m*/*z*) [M + H]^+^ calcd for C_21_H_17_N_4_OS_3_: 437.0559, found: 437.0561.

##### 4.1.1.2 2-(((4-Methyl-5-(thiophen-2-yl)-4*H*-1,2,4-triazol-3-yl)thio)methyl)benzo[*d*] thiazole (**6b**)

Yield 80%; mp 93–95 °C. ^1^H NMR (500 MHz, DMSO) δ 8.07 (dd, *J* = 7.9, 1.3 Hz, 1H), 7.96–7.90 (m, 1H), 7.79 (dd, *J* = 5.2, 1.1 Hz, 1H), 7.62 (dd, *J* = 3.7, 1.1 Hz, 1H), 7.54–7.47 (m, 1H), 7.47–7.39 (m, 1H), 7.25 (dd, *J* = 5.1, 3.6 Hz, 1H), 4.88 (s, 2H), 3.70 (s, 3H). ^13^C NMR (126 MHz, DMSO) δ: 168.18, 152.89, 151.19, 149.98, 135.76, 129.45, 128.71, 128.38, 128.30, 126.79, 125.86, 122.97, 122.77, 35.41, 32.44. HRMS (*m*/*z*) [M + H]^+^ calcd for C_15_H_13_N_4_OS_3_: 345.0302, found: 345.0298.

##### 4.1.1.3 2-(((4-(3-Methoxyphenyl)-5-(thiophen-2-yl)-4*H*-1,2,4-triazol-3-yl)thio)methyl)benzo[*d*]thiazole (**6c**)

Yield 87%; mp 163–164 °C.^1^H NMR (400 MHz, DMSO) δ 8.06 (d, *J* = 0.7 Hz, 1H), 7.96–7.90 (m, 1H), 7.64 (d, *J* = 0.9 Hz, 1H), 7.53–7.46 (m, 2H), 7.46–7.40 (m, 1H), 7.23–7.17 (m, 1H), 7.12 (t, *J* = 2.3 Hz, 1H), 7.07–6.95 (m, 2H), 6.78 (dd, *J* = 3.7, 1.2 Hz, 1H), 4.86 (s, 2H), 3.74 (s, 3H). ^13^C NMR (101 MHz, DMSO) δ: 168.38, 160.74, 152.88, 150.78, 135.76, 134.67, 131.53, 129.46, 128.28, 128.07, 127.74, 126.77, 125.83, 122.97, 122.76, 120.45, 116.95, 114.25, 56.12, 34.69. HRMS (*m*/*z*) [M + H]^+^ calcd for C_21_H_17_N_4_OS_3_: 437.0565, found: 437.0567.

##### 4.1.1.4 2-(((4-(4-Chlorophenyl)-5-(thiophen-2-yl)-4*H*-1,2,4-triazol-3-yl)thio)methyl)benzo[*d*]thiazole (**6d**)

Yield 91%; mp 164–166 °C. ^1^H NMR (400 MHz, DMSO) δ 8.08 (d, *J* = 7.9 Hz, 1H), 7.96 (d, *J* = 8.1 Hz, 1H), 7.68 (d, *J* = 7.1 Hz, 2H), 7.63–7.39 (m, 4H), 7.04 (t, *J* = 4.4 Hz, 1H), 6.82 (d, *J* = 3.7 Hz, 1H), 4.87 (s, 2H). ^13^C NMR (101 MHz, DMSO) δ: 168.24, 152.87, 150.82, 150.72, 136.03, 135.79, 132.56, 130.74, 130.59, 129.65, 128.36, 128.06, 127.85, 126.79, 125.85, 122.98, 122.75, 34.84. HRMS (*m*/*z*) [M + H]^+^ calcd for C_20_H_14_ClN_4_S_3_: 441.0069, found: 441.0066.

##### 4.1.1.5 4-(3-((Benzo[*d*]thiazol-2-ylmethyl)thio)-5-(thiophen-2-yl)-4*H*-1,2,4-triazol-4-yl)benzoic acid (**6e**)

Yield 93%; mp 271–272 °C. ^1^H NMR (400 MHz, DMSO) δ 8.07 (t, *J* = 9.1 Hz, 3H), 7.94 (d, *J* = 8.1 Hz, 1H), 7.64 (d, *J* = 5.1 Hz, 1H), 7.60–7.36 (m, 4H), 6.99 (t, *J* = 4.4 Hz, 1H), 6.73 (d, *J* = 3.8 Hz, 1H), 4.86 (s, 2H), 3.87 (brs, 1H). ^13^C NMR (101 MHz, DMSO) δ: 168.29, 167.44, 152.87, 150.72, 150.61, 136.35, 135.94, 135.77, 131.41, 129.62, 128.56, 128.36, 127.99, 127.84, 126.78, 125.84, 122.98, 122.77, 34.80. HRMS (*m*/*z*) [M + H]^+^ calcd for C_21_H_15_N_4_O_2_S_3_: 451.0357, found: 451.0358.

##### 4.1.1.6 3-(3-((Benzo[*d*]thiazol-2-ylmethyl)thio)-5-(thiophen-2-yl)-4*H*-1,2,4-triazol-4-yl)benzoic acid (**6f**)

Yield 82%; mp 213–215 °C. ^1^H NMR (400 MHz, DMSO) δ 13.41 (br, 1H), 8.19 (d, *J* = 1.7 Hz, 1H), 8.08 (dd, *J* = 8.1, 1.3 Hz, 1H), 8.02 (t, *J* = 1.8 Hz, 1H), 7.95 (dd, *J* = 7.9, 1.2 Hz, 1H), 7.79–7.70 (m, 2H), 7.67 (dd, *J* = 5.0, 1.1 Hz, 1H), 7.52 (dd, *J* = 7.1, 1.4 Hz, 1H), 7.45 (dd, *J* = 7.2, 1.3 Hz, 1H), 7.02 (dd, *J* = 5.1, 3.7 Hz, 1H), 6.76 (dd, *J* = 3.7, 1.1 Hz, 1H), 4.87 (s, 2H). ^13^C NMR (101 MHz, DMSO) δ: 168.17, 166.50, 152.86, 150.76, 150.67, 135.75, 133.95, 132.82, 131.92, 131.12, 129.62, 129.18, 128.34, 128.01, 127.90, 126.77, 125.85, 122.98, 122.74, 34.83. HRMS (*m*/*z*) [M + H]^+^ calcd for C_21_H_15_N_4_O_2_S_3_: 451.0357, found: 451.0350.

##### 4.1.1.7 2-(4-(3-((Benzo[*d*]thiazol-2-ylmethyl)thio)-5-(thiophen-2-yl)-4*H*-1,2,4-triazol-4-yl)phenyl)acetic acid (**6g**)

Yield 84%; mp 213–215 °C. ^1^H NMR (400 MHz, DMSO) δ 13.08 (br, 1H), 8.08 (d, *J* = 7.9 Hz, 1H), 7.96 (d, *J* = 8.1 Hz, 1H), 7.66 (d, *J* = 5.1 Hz, 1H), 7.57–7.38 (m, 6H), 7.02 (t, *J* = 4.4 Hz, 1H), 6.75 (d, *J* = 3.7 Hz, 1H), 4.88 (s, 2H), 3.72 (s, 2H). ^13^C NMR (101 MHz, DMSO) δ: 172.68, 168.41, 152.89, 150.96, 150.85, 138.82, 135.78, 131.92, 131.71, 129.48, 128.25, 128.18, 128.09, 127.71, 126.76, 125.81, 122.98, 122.76, 40.95, 34.57. HRMS (*m*/*z*) [M + H]^+^ calcd for C_22_H_17_N_4_O_2_S_3_: 465.0514, found: 465.0516.

##### 4.1.1.8 3-(4-(3-((Benzo[*d*]thiazol-2-ylmethyl)thio)-5-(thiophen-2-yl)-4*H*-1,2,4-triazol-4-yl)phenyl)propanoic acid (**6h**)

Yield 86%; mp 178–180 °C. ^1^H NMR (400 MHz, DMSO) δ 12.22 (brs, 1H), 8.08 (d, *J* = 7.9 Hz, 1H), 7.96 (d, *J* = 8.1 Hz, 1H), 7.65 (d, *J* = 5.0 Hz, 1H), 7.52 (t, *J* = 7.6 Hz, 1H), 7.52–7.32 (m, 5H), 7.00 (t, *J* = 4.2 Hz, 1H), 6.72 (d, *J* = 3.8 Hz, 1H), 4.87 (s, 2H), 2.95 (t, *J* = 7.6 Hz, 2H), 2.63 (t, *J* = 7.6 Hz, 2H). ^13^C NMR (101 MHz, DMSO) δ: 174.09, 168.41, 152.88, 150.93, 144.43, 135.77, 131.46, 130.48, 129.44, 128.32, 128.25, 128.12, 127.68, 126.77, 125.82, 122.97, 122.76, 35.26, 34.57, 30.45. HRMS (*m*/*z*) [M + H]^+^ calcd for C_23_H_19_N_4_O_2_S_3_: 479.0670, found: 479.0674.

##### 4.1.1.9 2-(((4-(4-Nitrophenyl)-5-(thiophen-2-yl)-4*H*-1,2,4-triazol-3-yl)thio)methyl)benzo [*d*]thiazole (**6i**)

Yield 90%; mp 166–168 °C. ^1^H NMR (400 MHz, DMSO) δ 8.41–8.35 (m, 2H), 8.07–8.02 (m, 1H), 7.95–7.90 (m, 1H), 7.85–7.80 (m, 2H), 7.67 (dd, *J* = 5.1, 1.2 Hz, 1H), 7.50 (dd, *J* = 7.2, 1.4 Hz, 1H), 7.43 (dd, *J* = 7.2, 1.3 Hz, 1H), 7.00 (dd, *J* = 5.1, 3.7 Hz, 1H), 6.77 (dd, *J* = 3.7, 1.2 Hz, 1H), 4.85 (s, 2H). ^13^C NMR (101 MHz, DMSO) δ 168.24, 152.87, 150.82, 150.72, 136.03, 135.79, 132.56, 130.74, 130.59, 129.65, 128.36, 128.06, 127.85, 126.79, 125.85, 122.98, 122.75, 34.84. HRMS (*m*/*z*) [M + H]^+^ calcd for C_20_H_14_N_5_O_2_S_3_: 452.0310, found: 452.0305.

##### 4.1.1.10 2-(((4-(4-Methoxyphenyl)-5-(thiophen-2-yl)-4*H*-1,2,4-triazol-3-yl)thio)methyl)quinoline (**6j**)

Yield 92%; mp 193–194 °C. ^1^H NMR (400 MHz, DMSO) δ 8.33 (d, *J* = 8.5 Hz, 1H), 7.95 (dd, *J* = 8.1, 1.4 Hz, 1H), 7.91 (dd, *J* = 8.4, 1.1 Hz, 1H), 7.73 (dd, *J* = 6.9, 1.5 Hz, 1H), 7.69–7.56 (m, 3H), 7.44–7.36 (m, 2H), 7.14–7.07 (m, 2H), 7.00 (dd, *J* = 5.1, 3.7 Hz, 1H), 6.77 (dd, *J* = 3.7, 1.2 Hz, 1H), 4.66 (s, 2H), 3.83 (s, 3H). ^13^C NMR (101 MHz, DMSO) δ 161.03, 157.41, 152.11, 150.90, 147.41, 137.33, 130.31, 129.97, 129.25, 128.84, 128.39, 128.34, 128.23, 127.53, 127.30, 127.02, 126.13, 121.81, 115.67, 56.05, 39.31. HRMS (*m*/*z*) [M + H]^+^ calcd for C_23_H_19_N_4_OS_2_: 431.1000, found: 431.1002.

##### 4.1.1.11 2-(((4-(3-Methoxyphenyl)-5-(thiophen-2-yl)-4*H*-1,2,4-triazol-3-yl)thio)methyl)quinoline (**6k**)

Yield 84%; mp 191–193 °C. ^1^H NMR (400 MHz, DMSO) δ 8.33 (d, *J* = 8.5 Hz, 1H), 7.99–7.87 (m, 2H), 7.73 (dd, *J* = 6.8, 1.5 Hz, 1H), 7.66–7.55 (m, 3H), 7.48 (t, *J* = 8.1 Hz, 1H), 7.19 (dd, *J* = 8.3, 2.6 Hz, 1H), 7.11 (t, *J* = 2.3 Hz, 1H), 7.06–6.98 (m, 2H), 6.76 (dd, *J* = 3.7, 1.2 Hz, 1H), 4.67 (s, 2H), 3.74 (s, 3H). ^13^C NMR (101 MHz, DMSO) δ: 160.68, 157.41, 151.63, 150.54, 147.41, 137.33, 134.90, 131.44, 130.31, 129.31, 128.83, 128.34, 128.25, 127.58, 127.31, 127.02, 121.81, 120.55, 116.81, 114.37, 56.10, 39.50. HRMS (*m*/*z*) [M + H]^+^ calcd for C_23_H_19_N_4_OS_2_: 431.1000, found: 431.0997.

##### 4.1.1.12 4-(4-Methoxyphenyl)-3-(thiophen-2-yl)-5-(((5-(trifluoromethyl)furan-2-yl)methyl)thio)-4*H*-1,2,4-triazole (**6l**)

Yield 93%; mp 125–128 °C. ^1^H NMR (400 MHz, DMSO) δ 7.62 (dd, *J* = 5.1, 1.2 Hz, 1H), 7.36–7.30 (m, 2H), 7.13–7.09 (m, 3H), 7.00 (dd, *J* = 5.1, 3.7 Hz, 1H), 6.79 (dd, *J* = 3.7, 1.2 Hz, 1H), 6.53 (d, *J* = 3.5 Hz, 1H), 4.44 (s, 2H), 3.84 (s, 3H). ^13^C NMR (101 MHz, DMSO) δ 161.06, 154.77, 151.10, 151.00, 139.81 (q, ^2^*J* = 41.6), 129.84, 129.37, 128.30, 128.22, 127.66, 126.03, 119.44 (q, ^1^*J* = 265.1) 115.68, 114.69, 114.66, 114.63, 114.60, 110.52, 56.03, 29.20. HRMS (*m*/*z*) [M + H]^+^ calcd for C_19_H_15_F_3_N_3_O_2_S_2_: 438.0558, found: 438.0558.

##### 4.1.1.13 2-(((4-(4-Methoxyphenyl)-5-(thiophen-2-yl)-4*H*-1,2,4-triazol-3-yl)thio)methyl)-5-methylpyridine (**6m**)

Yield 92%; mp 196–198 °C. ^1^H NMR (400 MHz, DMSO) δ 8.30 (d, *J* = 2.3 Hz, 1H), 7.61 (dd, *J* = 5.1, 1.2 Hz, 1H), 7.56 (dd, *J* = 7.9, 2.3 Hz, 1H), 7.35 (t, *J* = 2.6 Hz, 3H), 7.14–7.09 (m, 2H), 7.00 (dd, *J* = 5.1, 3.7 Hz, 1H), 6.79 (dd, *J* = 3.7, 1.2 Hz, 1H), 4.43 (s, 2H), 3.84 (s, 3H), 2.26 (s, 3H). ^13^C NMR (101 MHz, DMSO) δ: 161.02, 153.58, 152.26, 150.79, 149.68, 137.76, 132.31, 129.93, 129.20, 128.41, 128.22, 127.47, 126.10, 123.20, 115.68, 56.06, 38.32, 18.07. HRMS (*m*/*z*) [M + H]^+^ calcd for C_20_H_19_N_4_OS_2_: 395.1000, found: 395.1007.

##### 4.1.1.14 2-(((4-(4-Methoxyphenyl)-5-(thiophen-2-yl)-4*H*-1,2,4-triazol-3-yl)oxy)methyl)benzo[*d*]thiazole (**6n**)

Yield 80%; mp 155–157 °C. ^1^H NMR (400 MHz, DMSO) δ 8.07 (dd, *J* = 32.6, 8.1 Hz, 2H), 7.66 (d, *J* = 5.1 Hz, 1H), 7.61–7.30 (m, 4H), 7.11 (d, *J* = 8.4 Hz, 2H), 7.01 (t, *J* = 4.5 Hz, 1H), 6.90–6.72 (m, 1H), 5.49 (s, 2H), 3.84 (s, 3H). ^13^C NMR (101 MHz, DMSO) δ: 167.09, 160.59, 153.46, 152.84, 141.65, 135.45, 130.55, 129.70, 128.51, 128.24, 128.08, 126.90, 125.99, 125.91, 123.28, 122.93, 115.42, 56.00, 47.61. HRMS (*m*/*z*) [M + H]^+^ calcd for C_21_H_17_N_4_O_2_S_2_: 421.0793, found: 421.0793.

##### 4.1.1.15 2-(((4-(4-Methoxyphenyl)-5-(thiophen-2-yl)-4*H*-1,2,4-triazol-3-yl)oxy)methyl)quinoline (**6o**)

Yield 83%; mp 160–162 °C. ^1^H NMR (400 MHz, DMSO) δ 8.40 (d, *J* = 8.5 Hz, 1H), 8.02–7.96 (m, 2H), 7.78 (dd, *J* = 6.8, 1.5 Hz, 1H), 7.65–7.58 (m, 2H), 7.52 (d, *J* = 8.5 Hz, 1H), 7.47–7.42 (m, 2H), 7.14–7.09 (m, 2H), 6.99 (dd, *J* = 5.1, 3.7 Hz, 1H), 6.74 (dd, *J* = 3.8, 1.2 Hz, 1H), 5.30 (s, 2H), 3.84 (s, 3H). ^13^C NMR (101 MHz, DMSO) δ: 160.52, 157.14, 153.99, 147.43, 141.23, 137.71, 130.65, 130.43, 129.36, 129.09, 128.43, 128.17, 127.58, 127.09, 126.20, 120.22, 115.34, 56.00, 51.45. HRMS (*m*/*z*) [M + H]^+^ calcd for C_23_H_19_N_4_O_2_S: 415.1229, found: 415.1222.

##### 4.1.1.16 2-(((4-(4-Methoxyphenyl)-5-methyl-4*H*-1,2,4-triazol-3-yl)thio)methyl)benzo[*d*]-thiazole (**6p**)

Yield 58%; mp 184–186 °C. ^1^H NMR (500 MHz, DMSO) δ 8.05 (d, *J* = 7.4 Hz, 1H), 7.96–7.91 (m, 1H), 7.53–7.49 (m, 1H), 7.47–7.40 (m, 1H), 7.33 (d, *J* = 8.9 Hz, 2H), 7.08 (d, *J* = 8.9 Hz, 2H), 4.79 (s, 2H), 3.82 (s, 3H), 2.18 (s, 3H). ^13^C NMR (126 MHz, DMSO) δ: 168.64, 160.48, 153.64, 152.88, 149.07, 135.74, 128.88, 126.73, 125.90, 125.78, 122.94, 122.69, 115.45, 56.01, 34.50, 11.31. HRMS (*m*/*z*) [M + H]^+^ calcd for C_18_H_17_N_4_OS_2_: 369.0844, found: 369.0842.

##### 4.1.1.17 2-(((4-(4-Methoxyphenyl)-5-phenyl-4*H*-1,2,4-triazol-3-yl)thio)methyl)benzo[*d*]-thiazole (**6q**)

Yield 92%; mp 178–180 °C. ^1^H NMR (400 MHz, DMSO-*d*_6_) δ 8.08 (d, *J* = 7.9 Hz, 1H), 7.96 (d, *J* = 8.0 Hz, 1H), 7.52 (t, *J* = 7.3 Hz, 1H), 7.45 (t, *J* = 7.7 Hz, 1H), 7.39–7.37 (m, 5H), 7.33 (dd, *J* = 9.4, 2.9 Hz, 2H), 7.09–7.02 (m, 2H), 4.90 (s, 2H), 3.82 (s, 3H). ^13^C NMR (101 MHz, DMSO) δ: 168.49, 160.55, 155.32, 152.93, 151.48, 135.78, 130.26, 129.44, 129.07, 128.31, 127.10, 126.76, 126.55, 125.80, 122.96, 122.73, 115.52, 56.00, 34.39. HRMS (*m*/*z*) [M + H]^+^ calcd for C_23_H_19_N_4_OS_2_: 432.1079, found: 432.1079.

##### 4.1.1.18 2-(((5-(4-Chlorophenyl)-4-(4-methoxyphenyl)-4*H*-1,2,4-triazol-3-yl)thio)methyl)benzo[*d*]-thiazole (**6r**)

Yield 91%; mp 176–177 °C. ^1^H NMR (500 MHz, DMSO) δ 8.06 (dd, *J* = 8.1, 1.3 Hz, 1H), 7.98–7.92 (m, 1H), 7.51 (dd, *J* = 7.2, 1.3 Hz, 1H), 7.46–7.37 (m, 5H), 7.35–7.30 (m, 2H), 7.08–7.03 (m, 2H), 4.90 (s, 2H), 3.82 (s, 3H). ^13^C NMR (126 MHz, DMSO) δ: 168.28, 160.69, 154.45, 152.96, 151.77, 135.82, 135.18, 130.04, 129.38, 129.22, 126.73, 126.37, 126.01, 125.79, 122.97, 122.67, 115.64, 56.03, 34.49. HRMS (*m*/*z*) [M + H]^+^ calcd for C_23_H_18_ClN_4_OS_2_: 465.0611, found: 465.0606.

##### 4.1.1.19 4-(5-((Benzo[*d*]thiazol-2-ylmethyl)thio)-4-(4-methoxyphenyl)-4*H*-1,2,4-triazol-3-yl)benzonitrile (**6s**)

Yield 80%; mp 198–200 °C. ^1^H NMR (400 MHz, DMSO) δ 8.08 (d, *J* = 8.0 Hz, 1H), 7.96 (d, *J* = 8.1 Hz, 1H), 7.86 (d, *J* = 8.2 Hz, 2H), 7.54 (dd, *J* = 20.9, 8.0 Hz, 3H), 7.46 (d, *J* = 7.6 Hz, 1H), 7.38 (d, *J* = 8.7 Hz, 2H), 7.08 (d, *J* = 8.8 Hz, 2H), 4.93 (s, 2H), 3.83 (s, 3H). ^13^C NMR (101 MHz, DMSO) δ: 168.43, 160.74, 153.94, 152.91, 152.66, 135.75, 133.08, 131.34, 129.38, 128.84, 126.78, 126.10, 125.83, 122.97, 122.74, 118.69, 115.71, 112.71, 56.04, 34.26. HRMS (*m*/*z*) [M + H]^+^ calcd for C_24_H_18_N_5_OS_2_: 456.0953, found: 456.0955.

##### 4.1.1.20 2-(4-(5-((Benzo[*d*]thiazol-2-ylmethyl)thio)-4-(4-methoxyphenyl)-4*H*-1,2,4-triazol-3-yl)phenyl)acetonitrile (**6t**)

Yield 84%; mp 172–174 °C. ^1^H NMR (400 MHz, DMSO) δ 8.08 (d, *J* = 8.0 Hz, 1H), 7.96 (d, *J* = 8.1 Hz, 1H), 7.52 (t, *J* = 7.6 Hz, 1H), 7.44 (dd, *J* = 16.3, 8.0 Hz, 3H), 7.38–7.30 (m, 4H), 7.06 (d, *J* = 8.3 Hz, 2H), 4.91 (s, 2H), 4.05 (s, 2H), 3.82 (s, 3H). ^13^C NMR (101 MHz, DMSO) δ: 168.51, 160.57, 154.92, 152.92, 151.62, 135.77, 133.52, 130.38, 129.42, 129.28, 128.89, 128.82, 128.77, 126.76, 126.41, 125.81, 122.96, 122.74, 119.32, 115.55, 56.00, 34.36, 22.68. HRMS (*m*/*z*) [M + H]^+^ calcd for C_25_H_20_N_5_OS_2_: 470.1109, found: 470.1105.

##### 4.1.1.21 2-(4-(5-((Benzo[*d*]thiazol-2-ylmethyl)thio)-4-(4-methoxyphenyl)-4*H*-1,2,4-triazol-3-yl)phenyl)acetic acid (**6u**)

The mixture of compound **6t** (1 equiv) and KOH (1 equiv) in methyl alcohol–water (3:1) was stirred for 5 h, after which 150 mL of cold water was added dropwise to the mixture. The precipitate was filtered off to give crude solid, which was purified by automated-flash chromatography on silica gel eluting with a gradient of 0 → 60% EtOAc in *n*-hexane. Yield 80%; mp 223–225 °C. ^1^H NMR (400 MHz, DMSO-*d*_6_) δ 12.40 (brs, 1H), 8.08 (d, *J* = 7.9 Hz, 1H), 7.96 (d, *J* = 8.1 Hz, 1H), 7.52 (t, *J* = 7.6 Hz, 1H), 7.45 (t, *J* = 7.5 Hz, 1H), 7.37–7.30 (m, 4H), 7.25 (d, *J* = 7.9 Hz, 2H), 7.06 (d, *J* = 8.6 Hz, 2H), 4.90 (s, 2H), 3.82 (s, 3H), 3.58 (s, 2H). ^13^C NMR (101 MHz, DMSO) δ: 172.72, 172.69, 168.52, 168.48, 160.55, 155.23, 152.93, 151.42, 137.33, 135.78, 130.15, 129.47, 128.16, 126.75, 126.57, 125.80, 125.42, 122.96, 122.73, 115.54, 55.99, 34.40. HRMS (*m*/*z*) [M + H]^+^ calcd for C_25_H_21_N_4_O_3_S_2_: 489.1055, found: 489.1053.

##### 4.1.1.22 2-(4-(5-((Benzo[*d*]thiazol-2-ylmethyl)thio)-4-(4-chlorophenyl)-4*H*-1,2,4-triazol-3-yl)phenyl)acetonitrile (**6w**)

Yield 80%; mp 183–185 °C. ^1^H NMR (400 MHz, DMSO-*d*_6_) δ 8.07 (d, *J* = 7.9 Hz, 1H), 7.95 (d, *J* = 8.0 Hz, 1H), 7.59 (d, *J* = 8.6 Hz, 2H), 7.52 (t, *J* = 7.7 Hz, 1H), 7.48–7.33 (m, 7H), 4.90 (s, 2H), 4.05 (s, 2H). ^13^C NMR (101 MHz, DMSO) δ: 168.25, 154.82, 152.90, 151.03, 135.80, 135.30, 133.73, 132.96, 130.50, 130.06, 129.11, 128.90, 126.77, 126.13, 125.83, 122.98, 122.72, 119.27, 34.75, 22.70. HRMS (*m*/*z*) [M + H]^+^ calcd for C_24_H_17_ClN_5_S_2_: 474.0614, found: 474.0615.

##### 4.1.1.23 2-(4-(5-((Benzo[*d*]thiazol-2-ylmethyl)thio)-4-phenyl-4*H*-1,2,4-triazol-3-yl)phenyl)acetonitrile (**6v**)

Yield 81%; mp 163–165 °C. ^1^H NMR (400 MHz, DMSO-*d*_6_) δ 8.08 (d, *J* = 8.0 Hz, 1H), 7.96 (d, *J* = 8.0 Hz, 1H), 7.62–7.47 (m, 4H), 7.50–7.27 (m, 7H), 4.91 (s, 2H), 4.05 (s, 2H). ^13^C NMR (101 MHz, DMSO) δ: 168.42, 154.79, 152.90, 151.16, 135.76, 134.04, 133.61, 130.67, 130.50, 128.96, 128.82, 128.10, 126.77, 126.28, 125.82, 122.97, 122.75, 119.31, 34.46, 22.66. HRMS (*m*/*z*) [M + H]^+^ calcd for C_24_H_18_N_5_S_2_: 440.1004, found: 440.1021.

##### 4.1.1.24 2-(3-(5-((Benzo[*d*]thiazol-2-ylmethyl)thio)-4-phenyl-4*H*-1,2,4-triazol-3-yl)phenyl)acetonitrile (**6x**)

Yield 77%; mp 190–192 °C. ^1^H NMR (400 MHz, DMSO-*d*_6_) δ 8.08 (d, *J* = 7.9 Hz, 1H), 7.96 (d, *J* = 8.0 Hz, 1H), 7.57–7.51 (m, 5H), 7.48–7.29 (m, 5H), 7.15 (d, *J* = 7.5 Hz, 1H), 4.92 (s, 2H), 4.04 (s, 2H). ^13^C NMR (101 MHz, DMSO) δ: 168.39, 154.72, 152.90, 151.30, 135.76, 133.98, 132.49, 130.74, 130.51, 130.08, 129.61, 128.14, 128.07, 127.55, 127.27, 126.77, 125.83, 122.97, 122.75, 119.21, 34.47, 22.68. HRMS (*m*/*z*) [M + H]^+^ calcd for C_24_H_18_N_5_S_2_: 440.1004, found: 440.1013.

##### 4.1.1.25 2-(3-(5-((Benzo[*d*]thiazol-2-ylmethyl)thio)-4-(4-chlorophenyl)-4*H*-1,2,4-triazol-3-yl)phenyl)acetonitrile (**6y**)

Yield 71%; mp 179–181 °C. ^1^H NMR (400 MHz, DMSO-*d*_6_) δ 8.11–8.05 (m, 1H), 7.95 (d, *J* = 8.0 Hz, 1H), 7.60 (d, *J* = 8.7 Hz, 2H), 7.52 (td, *J* = 7.6, 1.4 Hz, 1H), 7.48–7.43 (m, 3H), 7.38 (d, *J* = 6.7 Hz, 4H), 4.90 (s, 2H), 4.06 (s, 2H). ^13^C NMR (101 MHz, DMSO) δ: 168.25, 154.82, 152.90, 151.02, 135.80, 135.30, 133.73, 132.96, 130.50, 130.06, 129.11, 128.90, 126.78, 126.13, 125.83, 122.98, 122.72, 119.27, 34.75, 22.69. HRMS (*m*/*z*) [M + H]^+^ calcd for C_24_H_17_ClN_5_S_2_: 474.0614, found: 474.0602.

##### 4.1.1.26 4-(5-((Benzo[*d*]thiazol-2-ylmethyl)thio)-4-phenyl-4*H*-1,2,4-triazol-3-yl)benzonitrile (**6z**)

Yield 85%; mp 183–185 °C. ^1^H NMR (400 MHz, DMSO-*d*_6_) δ 8.07 (d, *J* = 7.9 Hz, 1H), 7.96 (d, *J* = 8.0 Hz, 1H), 7.85 (d, *J* = 7.8 Hz, 2H), 7.63–7.48 (m, 6H), 7.45 (d, *J* = 6.9 Hz, 3H), 4.95 (s, 2H). ^13^C NMR (101 MHz, DMSO) δ: 168.33, 153.82, 152.90, 152.20, 135.75, 133.76, 133.07, 131.21, 130.96, 130.66, 128.93, 128.03, 126.78, 125.83, 122.98, 122.74, 118.65, 112.80, 34.38. HRMS (*m*/*z*) [M + H]^+^ calcd for C_23_H_16_N_5_S_2_: 426.0847, found: 426.0866.

### 4.2 Biology

#### 4.2.1 Isolation and Culture of Human Cells

Leukocyte concentrates derived from freshly withdrawn blood (16 I.E. heparin/mL blood) of healthy adult male and female volunteers (18–65 years) were provided by the Department of Transfusion Medicine at the University Hospital of Jena, Germany. The experimental procedures were approved by the local ethical committee (approval no. 5050-01/17) and were performed in accordance with the guidelines and regulations. Written informed consent was obtained from all volunteers. According to a previously published procedure,^46^ neutrophils and peripheral blood mononuclear cells (PBMC) were isolated by density gradient centrifugation using lymphocyte separation medium (C-44010, Promocell, Heidelberg, Germany) after sedimentation of erythrocytes by dextran. Differentiation of monocytes to macrophages and their polarization to M1- and M2-like macrophage phenotypes was carried out as recently described.^47^ Briefly, monocytes were incubated with either 20 ng/mL GM-CSF or M-CSF (Cell Guidance Systems Ltd., Cambridge, UK) for 6 days in RPMI 1640 (Thermo Fisher Scientific, Schwerte, Germany) containing heat-inactivated fetal calf serum (FCS, 10% v/v), penicillin (100 U/mL), streptomycin (100 μg/mL), and l-glutamine (2 mmol/L). This yielded M0_GM-CSF_ and M0_M-CSF_ monocyte-derived macrophages (MDM). Afterward, LPS (100 ng/mL) and IFN-γ (20 ng/mL; Peprotech, Hamburg, Germany) were added to M0_GM-CSF_ for 24 h to obtain M1-MDM, whereas IL-4 (20 ng/mL; Peprotech) was added to M0_M-CSF_ to generate M2-MDM within 48 h.

#### 4.2.2 Determination of FLAP-Dependent 5-LO Product Formation in Intact Neutrophils for SAR

Human neutrophils (5 × 10^6^/mL in PBS containing 1 mM CaCl_2_ and 0.1% glucose) were preincubated with 0.1% DMSO (vehicle) or with the compounds at 37 °C for 15 min. After addition of 2.5 μM A23187 (Cayman Chemical/Biomol GmbH, Hamburg, Germany), the reaction was incubated for 10 min at 37 °C and then stopped by addition of 1 mL of methanol, and 30 μL of 1 N HCl plus 200 ng of PGB_1_ and 500 μL of PBS were added. Samples were then prepared by solid phase extraction on C18-columns (100 mg, UCT, Bristol, PA), and 5-LO products (LTB_4_ and its trans-isomers, and 5-H(p)ETE) were analyzed in the presence of internal standard PGB_1_ by RP-HPLC and UV detection as reported elsewhere.^48^

Cell and whole blood incubations were for LM metabololipidomics analysis.

To study if **6x** can modulate LM formation, M1- or M2-MDM (2 × 10^6^/mL) or neutrophils (5× 10^6^/mL) were preincubated with vehicle (DMSO 0.1%) or 3 μM **6x** for 30 min and then stimulated with 1% SACM in PBS containing 1 mM CaCl_2_ for 90 min at 37 °C and 5% CO_2_. For the whole blood studies, freshly withdrawn whole blood in Li-heparin Monovettes (Sarstedt) from healthy adult donors that had not received any anti-inflammatory treatment the last 10 days was provided by the Institute of Transfusion Medicine, Jena University Hospital. The blood was preincubated with vehicle (DMSO, 0.1%) or 30 μM **6x** for 30 min and then stimulated with 1% SACM for 90 min at 37 °C and 5% CO_2._ Afterward, the reaction was stopped by adding 2 mL of ice-cold methanol containing deuterated LM standards (200 nM d8–5S-HETE, d4-LTB_4_, d5-LXA_4_, d5-RvD2, d4-PGE_2,_ and 10 μM d8-AA; Cayman Chemical/Biomol GmbH), samples were then processed for LM analysis using UPLC-MS-MS as described below.

#### 4.2.3 LM Metabololipidomics by UPLC-MS-MS

Samples obtained from MDM, neutrophils, and whole blood containing deuterated LM standards were kept at −20 °C for at least 60 min to allow protein precipitation. The extraction of LM was performed as recently published.^42^ In brief, after centrifugation (1200 × *g*; 4 °C; 10 min), acidified H_2_O (9 mL; final pH = 3.5) was added, and samples were extracted on solid phase cartridges (Sep-Pak Vac 6 cm^3^ 500 mg/6 mL C18; Waters, Milford, MA, USA). Samples were loaded on the cartridges after equilibration with methanol followed by H_2_O. After being washed with H_2_O and *n*-hexane, samples were eluted with methyl formate (6 mL). The solvent was fully evaporated using an evaporation system (TurboVap LV, Biotage, Uppsala, Sweden) and the residue was resuspended in 150 μL methanol/water (1:1, v/v) for UPLC-MS-MS analysis. LM were analyzed with an Acquity UPLC system (Waters, Milford, MA, USA) and a QTRAP 5500 Mass Spectrometer (ABSciex, Darmstadt, Germany) equipped with a Turbo V Source and electrospray ionization. LM were eluted using an ACQUITY UPLC BEH C18 column (1.7 μm, 2.1 mm × 100 mm; Waters, Eschborn, Germany) heated at 50 °C with a flow rate of 0.3 mL/min and a mobile phase consisting of methanol–water–acetic acid at a ratio of 42:58:0.01 (v/v/v) that was ramped to 86:14:0.01 (v/v/v) over 12.5 min and then to 98:2:0.01 (v/v/v) for 3 min.^42^ The QTRAP 5500 was run in negative ionization mode using scheduled multiple reaction monitoring (MRM) coupled with information-dependent acquisition. The scheduled MRM window was 60 s, optimized LM parameters were adopted,^42^ with a curtain gas pressure of 35 psi. The retention time and at least six diagnostic ions for each LM were confirmed by means of an external standard for each and every LM (Cayman Chemical/Biomol GmbH). Quantification was achieved by calibration curves for each LM; linear calibration curves were obtained and gave *r*^2^ values of 0.998 or higher. The limit of detection for each targeted LM was determined as described.^42^ For UPLC-MS-MS analysis, the quantification limit was 3 pg/sample, and this value was taken to express the fold increase for samples where the LM was not detectable (n.d.).

#### 4.2.4 Determination of 5-LOX Product Formation in Cell-free Assays

Human recombinant 5-LOX was expressed in *E. coli* BL21 (DE3) transformed with the pT35-LO plasmid and purified using affinity chromatography on an ATP-agarose column as previously published.^49^ 5-LOX (0.5 μg) was preincubated with test compounds at different concentrations or vehicle (0.1% DMSO) in 1 mL of PBS pH 7.4 containing EDTA (1 mM). After 15 min, samples were prewarmed for 30 s at 37 °C before CaCl_2_ (2 mM) and AA (20 μM) were added to initiate 5-LO product formation. After 10 min at 37 °C, 1 mL of ice-cold methanol containing PGB_1_ (200 ng) as standard and 530 μL PBS containing 0.06 M HCl were added and 5-LO products were extracted via solid-phase extraction.^50^ The resulting methanol-eluate was analyzed for all-trans isomers of LTB_4_ and 5-H(p)ETE by RP-HPLC utilizing a C-18 Radial-PAK column (Waters, Eschborn, Germany) as previously reported.^50^

#### 4.2.5 Cell Viability Assays

For analysis of the effects of test compounds on cell viability, cells were incubated with 3-(4,5-dimethylthiazol-2-yl)-2,5-diphenyltetrazolium bromide (MTT, 5 mg/mL, 20 μL; Sigma-Aldrich, Munich, Germany) for 2–3 h at 37 °C (5% CO_2_) in the darkness. The formazan product was solubilized with sodium dodecyl sulfate (SDS, 10% in 20 mM HCl) and the absorbance was monitored at 570 nm (Multiskan Spectrum microplate reader, Thermo Fisher Scientific, Schwerte, Germany).

### 4.3 Computational Studies

#### 4.3.1 Molecular Docking

All publicly released FLAP structures (PDB codes: 2Q7M, 2Q7R,^19^ 6VGC, 6VGI^20^) were considered for selection of the most appropriate crystal to be used in the modeling studies. 6VGC was selected based on the crystal resolution, generating the lowest RMSD variance in the binding poses of each cocrystallized ligand. The designed compounds were drawn by using Maestro interface.^35^ Chemical states and atom types at pH 7.0 ± 2.0 were assigned with OPLS4 force field by utilizing *LigPrep* for the ligand structures,^51^ and *Protein Preparation Wizard* for the protein structures.^52^ van der Waals radius scaling factor and partial charge cutoffs were left with the default values, 1.0 and 0.25. Docking simulations were issued with *Glide* in standard precision (SP) mode.^53,54^ The highest-ranking pose was selected for the generation of MD simulations.

#### 4.3.2 Molecular Dynamics

Four copies (200 ns) were conducted after generating the system with *System Builder* utility. The SPC model was used for water molecules, and POPC atoms were used for generating the lipid bilayer. The membrane was positioned by obtaining the coordinates from the OPM database.^34^ Coulombic interaction cut off was applied as 9.0 Å. The simulation system was neutralized with Na^+^ ions. The atom types were issued with the OPLS4 force field. Later, the simulations were run with *Desmond.*(55) The simulations started with a multistep relaxation protocol; (i) for the first step of the minimization, Brownian Dynamics was applied with NVT ensemble by the heating system to 10 K with small timesteps and applying restraints on solute heavy atoms for 100 ps. (ii) Subsequently, relaxation continued at 100 K with the H_2_O Barrier and Brownian NPT ensemble, membrane restrained in the *z* axis and also protein restrained. (iii) Next, the same approach was applied, but this time with the NPgT ensemble. (iv) Later, the whole restraints were removed, and the simulation was run with NPT ensemble at 300 K for 200 ns. Simulations were analyzed by utilizing *Simulation Interaction Diagram* interface of Maestro^35^ and Gromacs^56^ scripts.

## Supporting Information Available

Representative NMR spectra and Graphical presentations of Bioactivity Assays for the compounds tested can be found in Supporting Information (PDF)
