# Supplementary material for: Substituted 1,2,4-Triazoles as Novel and Selective Inhibitors of Leukotriene Biosynthesis Targeting 5-Lipoxygenase-Activating Protein
Source: ACS Omega. 2023 Aug 18;8(34):31293–304. doi: 10.1021/acsomega.3c03682 (PMC10468765; doi:10.1021/acsomega.3c03682)
Supplement: Supplementary file 1 — ao3c03682_si_001.pdf [file ao3c03682_si_001.pdf]

SUPPORTING INFORMATION

Substituted 1,2,4-triazoles as novel and selective inhibitors of leukotriene biosynthesis targeting 5-lipoxygenase-activating protein (FLAP)

Abdurrahman Olğaç,[¥,§] İrfan Çapan,[‡] Philipp Dahlke,[†] Paul M. Jordan,[†] Oliver Werz,[†,*] Erden Banoglu[¥,*]

[¥]Department of Pharmaceutical Chemistry, Faculty of Pharmacy, Gazi University, Yenimahalle, 06560 Ankara, Turkey

[§]Department of Drug Discovery, Evias Pharmaceutical R&D Ltd., 06830 Ankara, Turkey

[‡]Department of Material and Material Processing Technologies Technical Sciences Vocational College, Gazi University, 06374 Ankara, Turkey

[†]Department of Pharmaceutical/Medicinal Chemistry, Institute of Pharmacy, Friedrich Schiller University Jena, Philosophenweg 14, D-7743 Jena, Germany

Table of Contents

Figure S1. Activity of isolated recombinant 5-LO is not affected by 6x. Purified 5-LO enzyme was preincubated with 0.3, 1, 3 and 10 µM 6x or 3 µM zileuton as positive control before stimulation with 20 µM exogenous AA for 5-LOX product formation, analyzed by HPLC. Data are given as mean + SEM and single values of n = 3 independent experiments, shown as percentage of vehicle control (= 100%). 6

Figure S2. Modulation of lipid mediator profiles in activated 5-LOX-rich immune cells by 6x. M1-MDM were incubated with 6x or vehicle (DMSO 0.1%) for 90 min and LDH release was measured in the supernatant with representing 100% integrity and lysis control (triton) representing 0% integrity. Data are shown as means ± S.E.M. of single data points. n = 3 individual experiments. 7

Figure S3. Docking derived binding mode of 6x visualized in FLAP active site (A) and comparison with the most populated binding pose derived from the MD simulation, docking pose is shown semi-transparent (B). The figures were generated with PyMOL [1]. Docking derived 2D interaction plot of 6x with FLAP (C). 8

Figure S4. (A) RMSD figures each simulation of compound 6x simulated within the AA binding pocket of FLAP for 200 ns of MD. (B) Timeline representation of contacts during MD simulation of 6x with FLAP. 9

Synthesis of intermediates 3a-r 10

Synthesis of intermediates 4a-r 10

Synthesis of intermediate 4-(4-methoxyphenyl)-5-(thiophen-2-yl)-4H-1,2,4-triazol-3-ol (5) 10

Table S1. Yield, melting point and HRMS analysis data of intermediate compounds 3a-3r. 11

Table S2. Yield, melting point and HRMS analysis data of intermediate compounds 4a-4r, 5. 12

Figure S5. ^{1}H-NMR Spectrum of 6a 13

Figure S6. $^{13}C_{APT}$-NMR Spectrum of 6a 13

Figure S7. ^{1}H-NMR Spectrum of 6b 14

Figure S8. $^{13}C_{APT}$-NMR Spectrum of 6b 14

Figure S9. ^{1}H-NMR Spectrum of 6c 15

Figure S10. $^{13}C_{APT}$-NMR Spectrum of 6c 15

Figure S11. ^{1}H-NMR Spectrum of 6d ... 16
Figure S12. $^{13}C_{APT}$-NMR Spectrum of 6d ... 16
Figure S13. ^{1}H-NMR Spectrum of 6e ... 17
Figure S14. $^{13}C_{APT}$-NMR Spectrum of 6e ... 17
Figure S15. ^{1}H-NMR Spectrum of 6f ... 18
Figure S16. $^{13}C_{APT}$-NMR Spectrum of 6f ... 18
Figure S17. ^{1}H-NMR Spectrum of 6g ... 19
Figure S18. $^{13}C_{APT}$-NMR Spectrum of 6g ... 19
Figure S19. ^{1}H-NMR Spectrum of 6h ... 20
Figure S20. $^{13}C_{APT}$-NMR Spectrum of 6h ... 20
Figure S21. ^{1}H-NMR Spectrum of 6i ... 21
Figure S22. $^{13}C_{APT}$-NMR Spectrum of 6i ... 21
Figure S23. ^{1}H-NMR Spectrum of 6j ... 22
Figure S24. $^{13}C_{APT}$-NMR Spectrum of 6j ... 22
Figure S25. ^{1}H-NMR Spectrum of 6k ... 23
Figure S26. $^{13}C_{APT}$-NMR Spectrum of 6k ... 23
Figure S27. ^{1}H-NMR Spectrum of 6l ... 24
Figure S28. $^{13}C_{APT}$-NMR Spectrum of 6l ... 24
Figure S29. ^{1}H-NMR Spectrum of 6m ... 25
Figure S30. $^{13}C_{APT}$-NMR Spectrum of 6m ... 25
Figure S31. ^{1}H-NMR Spectrum of 6n ... 26
Figure S32. $^{13}C_{APT}$-NMR Spectrum of 6n ... 26
Figure S33. ^{1}H-NMR Spectrum of 6o ... 27
Figure S34. $^{13}C_{APT}$-NMR Spectrum of 6o ... 27
Figure S35. ^{1}H-NMR Spectrum of 6p ... 28
Figure S36. $^{13}C_{APT}$-NMR Spectrum of 6p ... 28
Figure S37. ^{1}H-NMR Spectrum of 6q ... 29
Figure S38. $^{13}C_{APT}$-NMR Spectrum of 6q ... 29
Figure S39. ^{1}H-NMR Spectrum of 6r ... 30
Figure S40. $^{13}C_{APT}$-NMR Spectrum of 6r ... 30
Figure S41. ^{1}H-NMR Spectrum of 6s ... 31
Figure S42. $^{13}C_{APT}$-NMR Spectrum of 6s ... 31

Figure S43. ^{1}H-NMR Spectrum of 6t 32
Figure S44. $^{13}C_{APT}$-NMR Spectrum of 6t 32
Figure S45. ^{1}H-NMR Spectrum of 6u 33
Figure S46. $^{13}C_{APT}$-NMR Spectrum of 6u 33
Figure S47. ^{1}H-NMR Spectrum of 6v 34
Figure S48. $^{13}C_{APT}$-NMR Spectrum of 6v 34
Figure S49. ^{1}H-NMR Spectrum of 6w 35
Figure S50. $^{13}C_{APT}$-NMR Spectrum of 6w 35
Figure S51. ^{1}H-NMR Spectrum of 6x 36
Figure S52. $^{13}C_{APT}$-NMR Spectrum of 6x 36
Figure S53. ^{1}H-NMR Spectrum of 6y 37
Figure S54. $^{13}C_{APT}$-NMR Spectrum of 6y 37
Figure S55. ^{1}H-NMR Spectrum of 6z 38
Figure S56. $^{13}C_{APT}$-NMR Spectrum of 6z 38
Figure S57. HRMS Spectrum of 6a 39
Figure S58. HRMS Spectrum of 6b 39
Figure S59. HRMS Spectrum of 6c 39
Figure S60. HRMS Spectrum of 6d 39
Figure S61. HRMS Spectrum of 6e 39
Figure S62. HRMS Spectrum of 6f 40
Figure S63. HRMS Spectrum of 6g 40
Figure S64. HRMS Spectrum of 6h 40
Figure S65. HRMS Spectrum of 6i 40
Figure S66. HRMS Spectrum of 6j 40
Figure S67. HRMS Spectrum of 6k 41
Figure S68. HRMS Spectrum of 6l 41
Figure S69. HRMS Spectrum of 6m 41
Figure S70. HRMS Spectrum of 6n 41
Figure S71. HRMS Spectrum of 6o 41
Figure S72. HRMS Spectrum of 6p 42
Figure S74. HRMS Spectrum of 6r 42
Figure S75. HRMS Spectrum of 6s 42

Figure S76. HRMS Spectrum of 6t 42
Figure S77. HRMS Spectrum of 6u 43
Figure S78. HRMS Spectrum of 6v 43
Figure S79. HRMS Spectrum of 6w 43
Figure S80. HRMS Spectrum of 6x 43
Figure S81. HRMS Spectrum of 6y 43
Figure S82. HRMS Spectrum of 6z 44
Figure S83. IC_{50} Curves of Compounds 6a, 6c, 6d, 6j-l, 6n, 6o, 6q, 6s, 6t, 6v-z 44

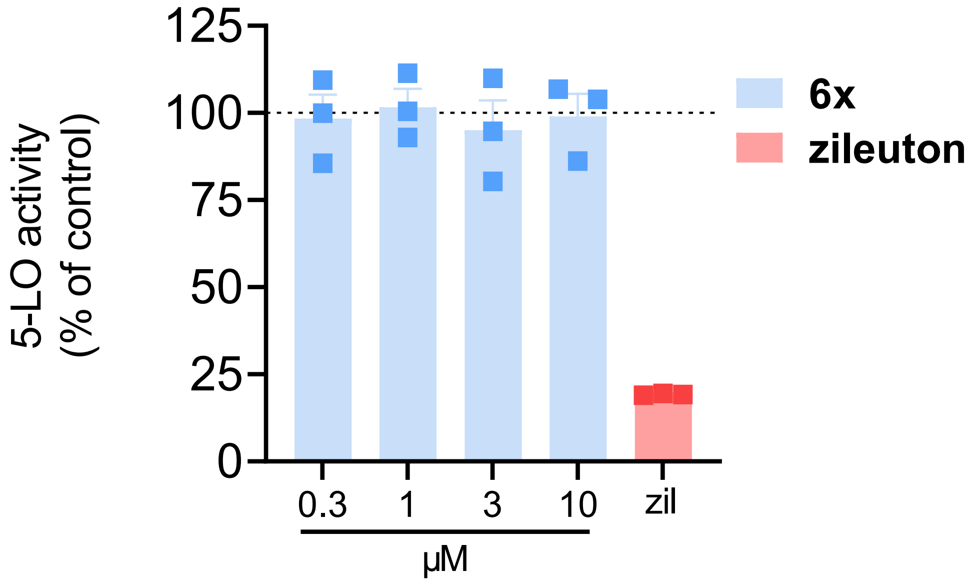

Figure S1. Activity of isolated recombinant 5-LO is not affected by 6x. Purified 5-LO enzyme was preincubated with 0.3, 1, 3 and 10 µM 6x or 3 µM zileuton as positive control before stimulation with 20 µM exogenous AA for 5-LOX product formation, analyzed by HPLC. Data are given as mean + SEM and single values of n = 3 independent experiments, shown as percentage of vehicle control (= 100%).

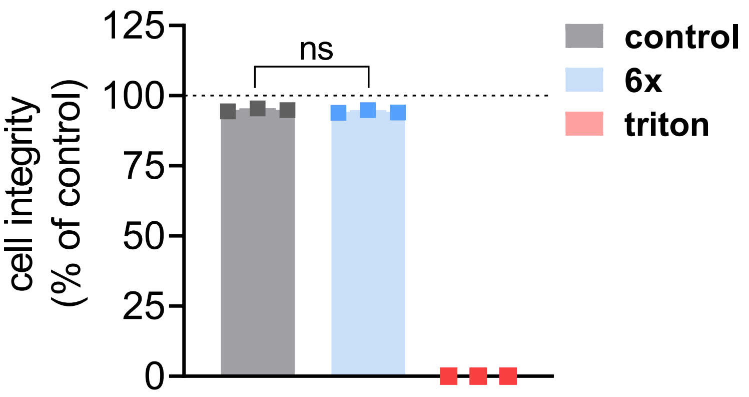

Figure S2. Modulation of lipid mediator profiles in activated 5-LOX-rich immune cells by **6x**. M1-MDM were incubated with 6x or vehicle (DMSO 0.1%) for 90 min and LDH release was measured in the supernatant with representing 100% integrity and lysis control (triton) representing 0% integrity. Data are shown as means ± S.E.M. of single data points. n = 3 individual experiments.

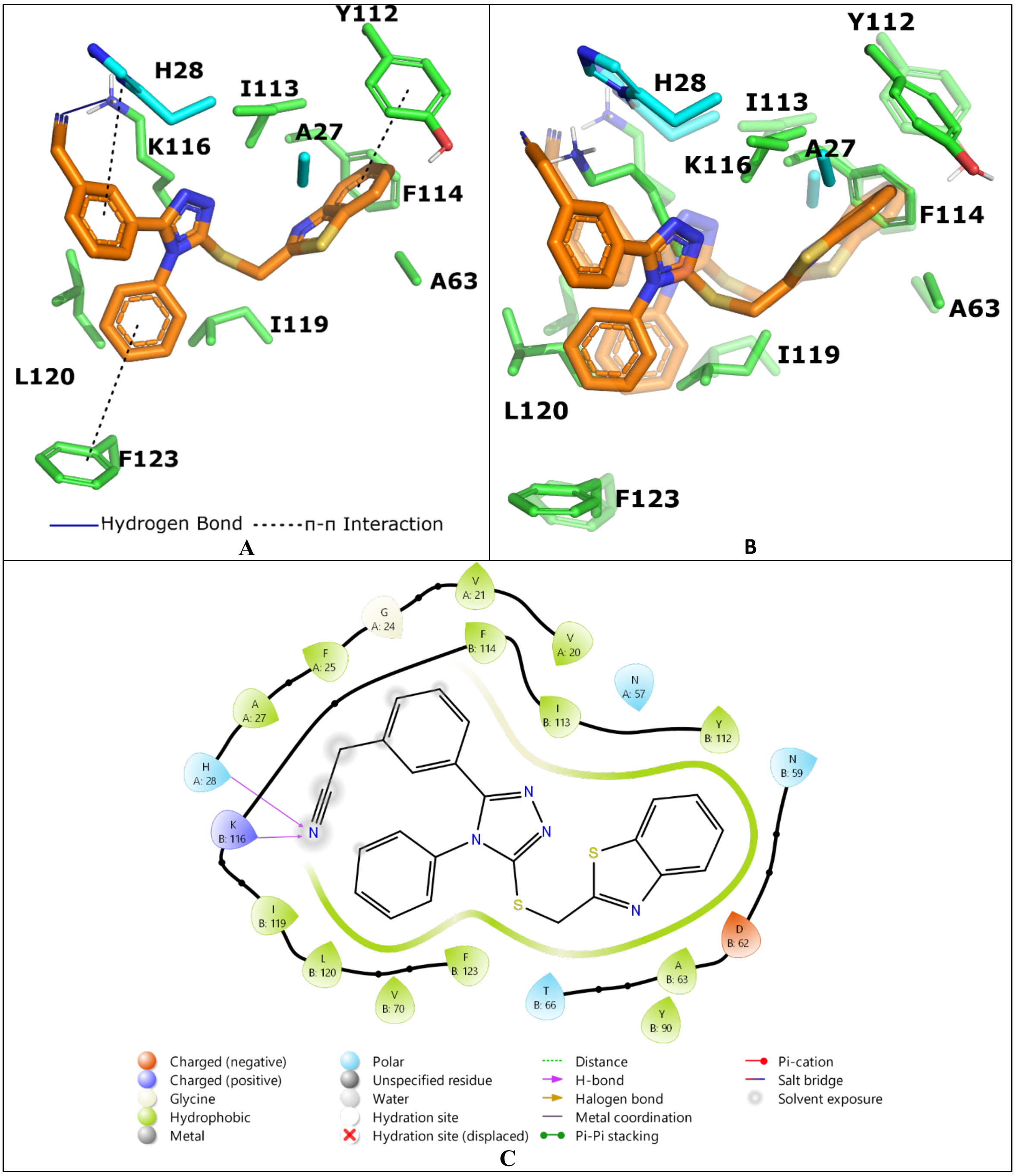

Figure S3. Docking derived binding mode of **6x** visualized in FLAP active site (A) and comparison with the most populated binding pose derived from the MD simulation, docking pose is shown semi-transparent (B). The figures were generated with PyMOL [1]. Docking derived 2D interaction plot of 6x with FLAP (C).

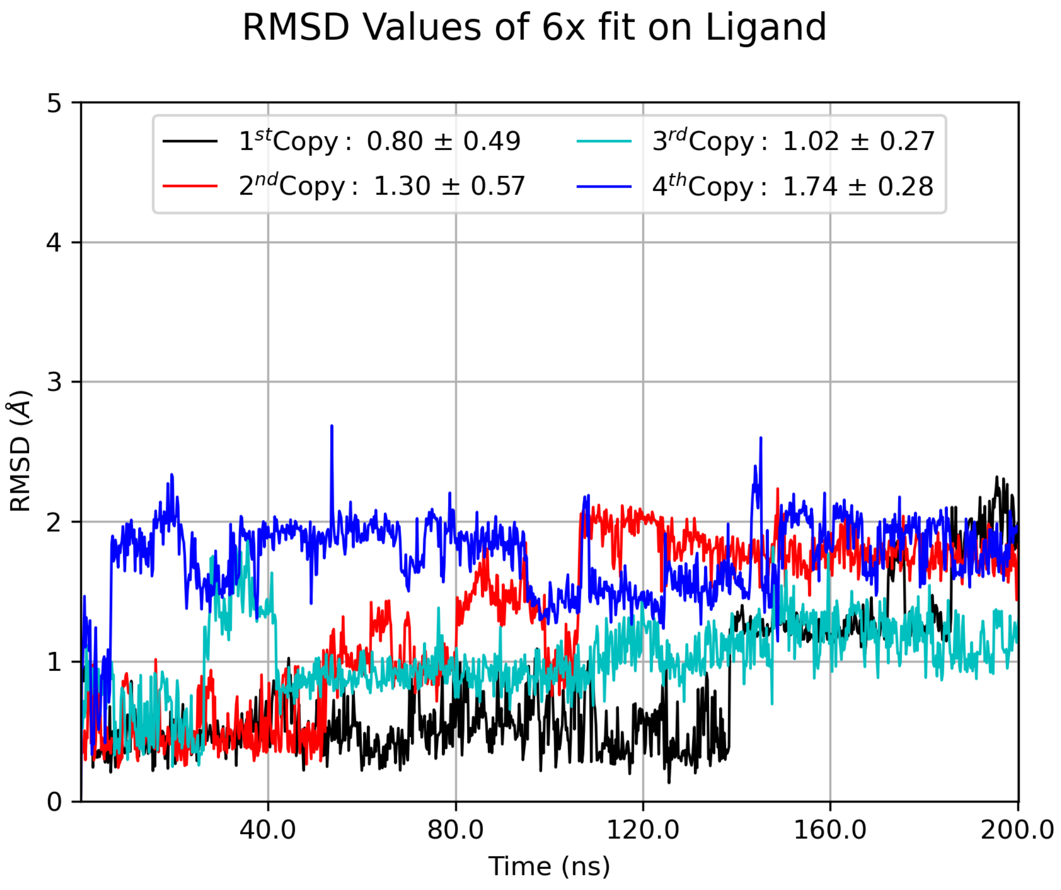

A

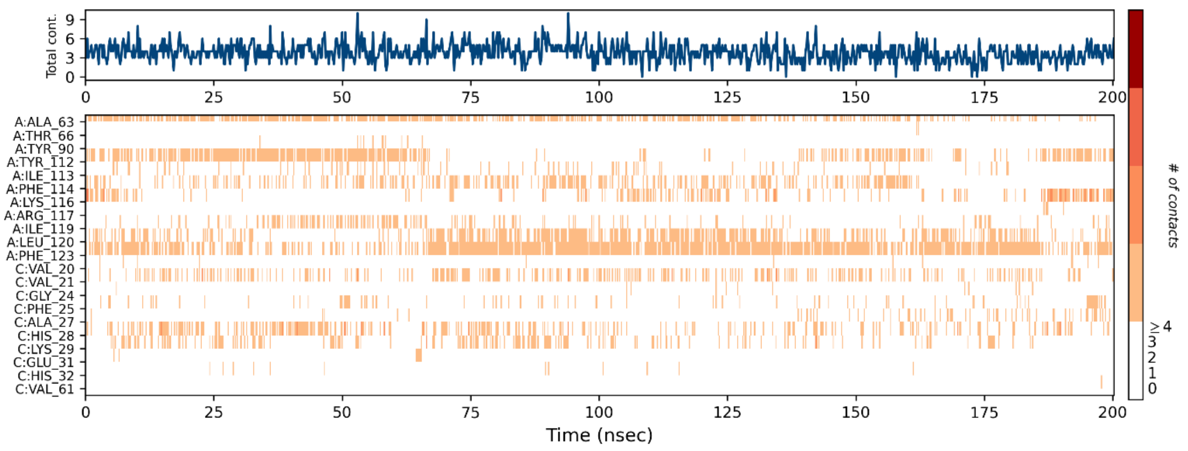

B

Figure S4. (A) RMSD figures each simulation of compound **6x** simulated within the AA binding pocket of FLAP for 200 ns of MD. **(B)** Timeline representation of contacts during MD simulation of **6x** with FLAP.

Synthesis of intermediates 3a-r

Step 1: To a solution of the appropriate ester derivative (1 eq) in ethyl alcohol (10 ml) was added hydrazine hydrate (50-60% solution in water) (5 eq) and then the mixture was refluxed for 5 h. After evaporating the reaction medium, the crude product was dissolved with 5 ml of methanol and poured into 100 ml of water, acidified with 2-3 drops of conc. HCl. The precipitated white solid was filtered off and dried. It was used in the next step without the need for further purification. Step 2: To a solution of appropriate acetohydrazide derivative (1 eq) in ethyl alcohol (15 ml) was added the appropriate isothiocyanate (1 eq) and the mixture refluxed for 4 h. The solvent was evaporated, the crude solid obtained was washed with cold ethyl alcohol and then with water and dried in a vacuum oven. The synthesized compounds containing the hydrazine-1-carbothioamide functional group were used in the next reaction step without any additional purification method.

Synthesis of intermediates 4a-r

The solution of the appropriate thiosemicarbazide derivative (**3a-r**) (1 eq) in either 2N NaOH (10 ml) or in saturated $NaHCO_3$ solution (10 ml) was refluxed for 3h or 24 h, respectively. After cooling the reaction mixture to rt, it was poured onto 100 ml of water. The pH was adjusted to 3-4 with conc. HCl, the precipitate was filtered off, washed with water, and recrystallized from ethanol.

Synthesis of intermediate 4-(4-methoxyphenyl)-5-(thiophen-2-yl)-4H-1,2,4-triazol-3-ol (5)

The solution of compound **4a** (1 eq) refluxed in 4N NaOH (10 ml) for 24 hours and hydrogen peroxide solution (50% aq) (10 ml) was added and then stirred at reflux temperature for another 24 hours. The clear homogeneous mixture was poured into 100 ml of water, adjusted to pH 4-5 with conc. HCl. The precipitated solid was filtered, dried, and recrystallized from ethanol.

Table S1. Yield, melting point and HRMS analysis data of intermediate compounds 3a-3r.

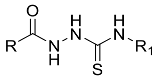

#	R	R_1	Yield (%)	mp (°C)	Molecule Formula	HRMS (m/z) $[M+H]^+$	
						calcd	found
**3a**	4-Cl-Ph	4-OMe-Ph	95	181-182	$C_{15}H_{14}ClN_3O_2S$	336.0574	336.0560
**3b**	thiophen-2-yl	4-OMe-Ph	97	164-165	$C_{13}H_{13}N_3O_2S_2$	308.0527	308.0525
**3c**	thiophen-2-yl	3-OMe-Ph	92	170-172	$C_{13}H_{13}N_3O_2S_2$	308.0527	308.0520
**3d**	thiophen-2-yl	4-COOH-Ph	93	198-200	$C_{13}H_{11}N_3O_3S_2$	322.0320	322.0315
**3e**	thiophen-2-yl	3-COOH-Ph	85	195-197	$C_{13}H_{11}N_3O_3S_2$	322.0320	322.0305
**3f**	thiophen-2-yl	4-NO_2-Ph	90	192-193	$C_{12}H_{10}N_4O_3S_2$	323.0273	323.0283
**3g**	thiophen-2-yl	4-Cl-Ph	88	193-195	$C_{12}H_{10}ClN_3OS_2$	312.0032	312.0038
**3h**	thiophen-2-yl	4-CH_2COOH-Ph	84	177-179	$C_{14}H_{13}N_3O_3S_2$	336.0477	336.0480
**3i**	thiophen-2-yl	4-CH_2CH_2COOH-Ph	82	182-184	$C_{15}H_{15}N_3O_3S_2$	350.0633	350.0625
**3j**	4-CN-Ph	4-OMe-Ph	89	165-167	$C_{16}H_{14}N_4O_2S$	327.0916	327.0910
**3k**	4-CN-Ph	Ph	91	154-156	$C_{15}H_{12}N_4OS$	297.0810	297.0808
**3l**	4-CH_2CN-Ph	4-OMe-Ph	87	169-171	$C_{17}H_{16}N_4O_2S$	341.1072	341.1072
**3m**	4-CH_2CN-Ph	Ph	86	184-186	$C_{16}H_{14}N_4OS$	311.0967	311.0956
**3n**	3-CH_2CN-Ph	Ph	83	192-194	$C_{16}H_{14}N_4OS$	311.0967	311.0972
**3o**	4-CH_2CN-Ph	4-Cl-Ph	80	188-190	$C_{16}H_{13}ClN_4OS$	345.0577	345.0580
**3p**	Ph	4-OMe-Ph	93	182-184	$C_{15}H_{15}N_3O_2S$	302.0963	302.0966
**3q**	Me	4-OMe-Ph	90	152-154	$C_{10}H_{13}N_3O_2S$	240.0807	240.0808
**3r**	thiophen-2-yl	Me	95	158-160	$C_7H_9N_3OS_2$	216.0265	216.0260

Table S2. Yield, melting point and HRMS analysis data of intermediate compounds 4a-4r, 5.

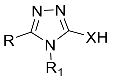

#	R	R_1	X	Yield (%)	mp (°C)	Molecule Formula	HRMS (m/z) $[M+H]^+$	
							calcd	found
**4a**	4-Cl-Ph	4-OMe-Ph	S	92	241-243	$C_{15}H_{12}ClN_3OS$	318.0468	318.0452
**4b**	thiophen-2-yl	4-OMe-Ph	S	96	235-236	$C_{13}H_{11}N_3OS_2$	290.0422	290.0424
**4c**	thiophen-2-yl	3-OMe-Ph	S	92	183-184	$C_{13}H_{11}N_3OS_2$	290.0422	290.0424
**4d**	thiophen-2-yl	4-COOH-Ph	S	94	276-278	$C_{13}H_9N_3O_2S_2$	304.0214	304.0206
**4e**	thiophen-2-yl	3-COOH-Ph	S	88	292-294	$C_{13}H_9N_3O_2S_2$	304.0214	304.0210
**4f**	thiophen-2-yl	4-NO_2-Ph	S	90	230-231	$C_{12}H_8N_4O_2S_2$	305.0167	305.0167
**4g**	thiophen-2-yl	4-Cl-Ph	S	91	233-235	$C_{12}H_8ClN_3S_2$	293.9926	293.9920
**4h**	thiophen-2-yl	4-CH_2COOH-Ph	S	89	240-242	$C_{14}H_{11}N_3O_2S_2$	318.0371	318.0360
**4i**	thiophen-2-yl	4-CH_2CH_2COOH-Ph	S	85	236-238	$C_{15}H_{13}N_3O_2S_2$	332.0527	332.0539
**4j**	4-CN-Ph	4-OMe-Ph	S	83	245-247	$C_{16}H_{12}N_4OS$	309.0810	309.0808
**4k**	4-CN-Ph	Ph	S	88	248-249	$C_{15}H_{10}N_4S$	279.0704	279.0708
**4l**	4-CH_2CN-Ph	4-OMe-Ph	S	80	240-242	$C_{17}H_{14}N_4OS$	323.0967	323.0963
**4m**	4-CH_2CN-Ph	Ph	S	91	244-246	$C_{16}H_{12}N_4S$	293.0861	293.0861
**4n**	3-CH_2CN-Ph	Ph	S	84	232-234	$C_{16}H_{12}N_4S$	293.0861	293.0880
**4o**	4-CH_2CN-Ph	4-Cl-Ph	S	80	237-239	$C_{16}H_{11}ClN_4S$	327.0471	327.0472
**4p**	Ph	4-OMe-Ph	S	94	227-229	$C_{15}H_{13}N_3OS$	284.0858	284.0860
**4q**	Me	4-OMe-Ph	S	92	203-204	$C_{10}H_{11}N_3OS$	222.0701	222.0698
**4r**	thiophen-2-yl	Me	S	96	226-228	$C_{13}H_{11}N_3O_2S$	198.0160	198.0165
**5**	thiophen-2-yl	4-OMe-Ph	O	78	217-219	$C_7H_7N_3S_2$	274.0650	274.0647

Figure S5. ^{1}H-NMR Spectrum of 6a

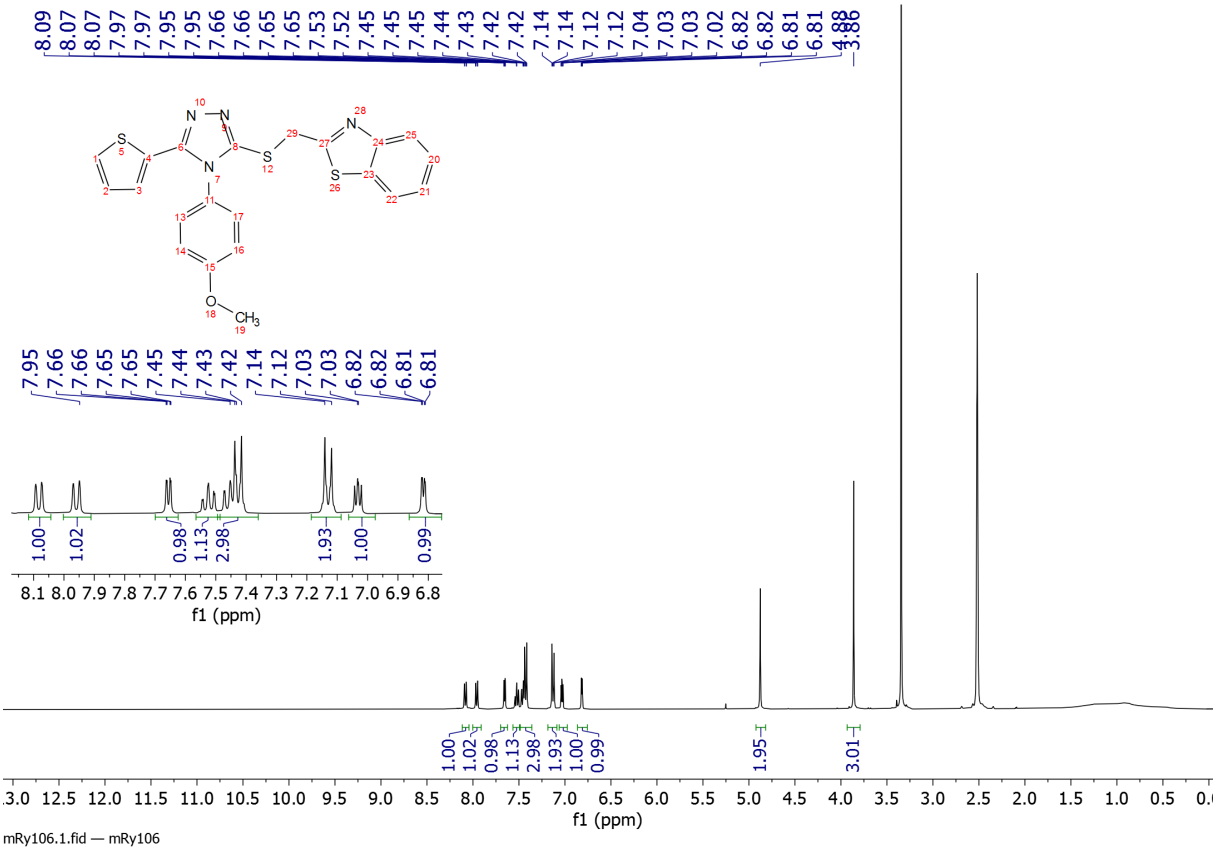

mRy106.1.fid — mRy106

Figure S6. $^{13}C_{APT}$-NMR Spectrum of 6a

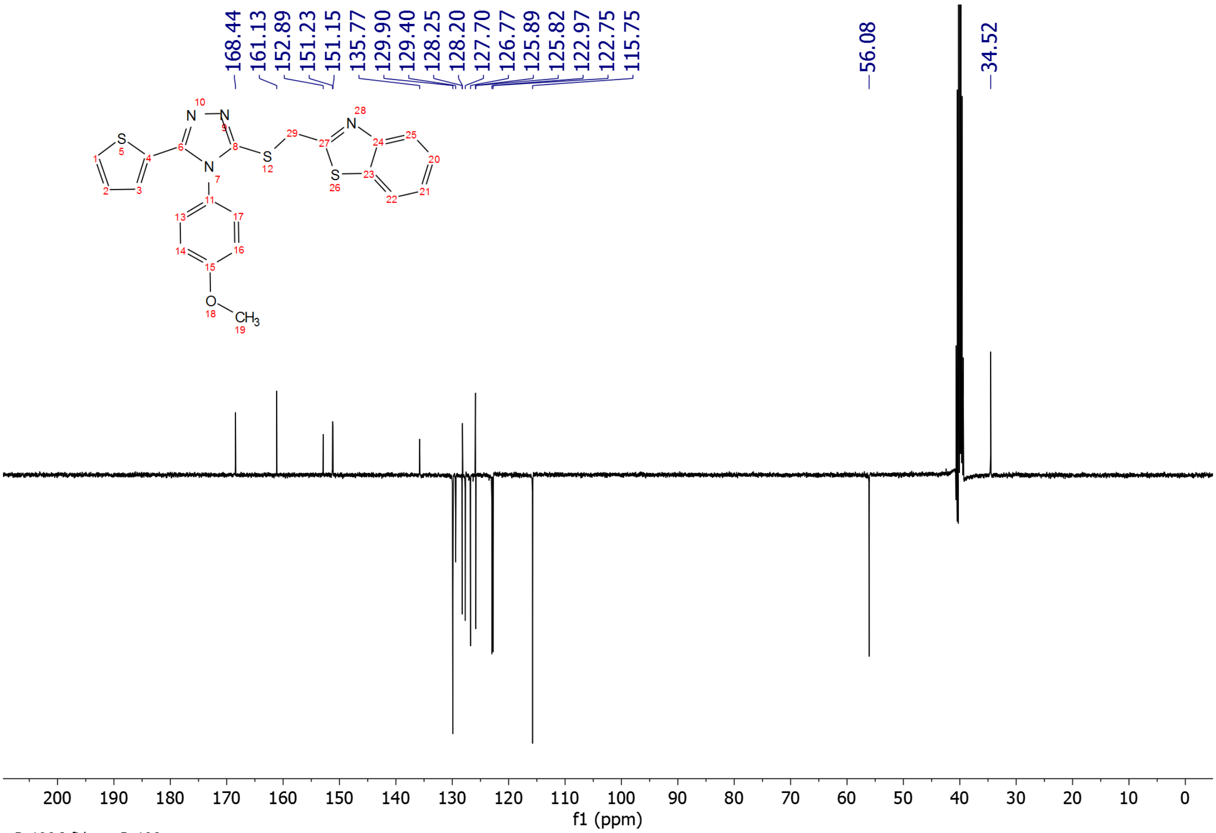

mRy106.2.fid — mRy106

Figure S7. ^{1}H-NMR Spectrum of 6b

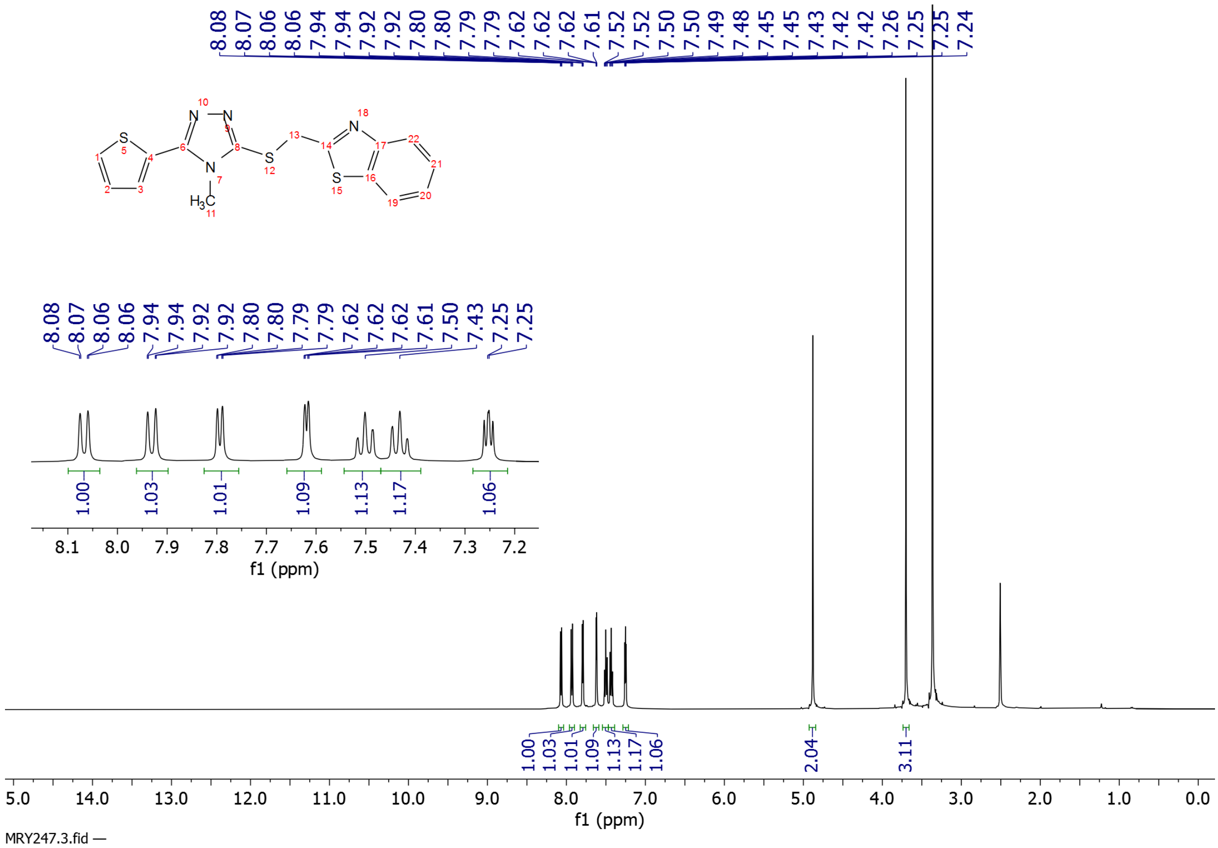

Figure S8. $^{13}C_{APT}$-NMR Spectrum of 6b

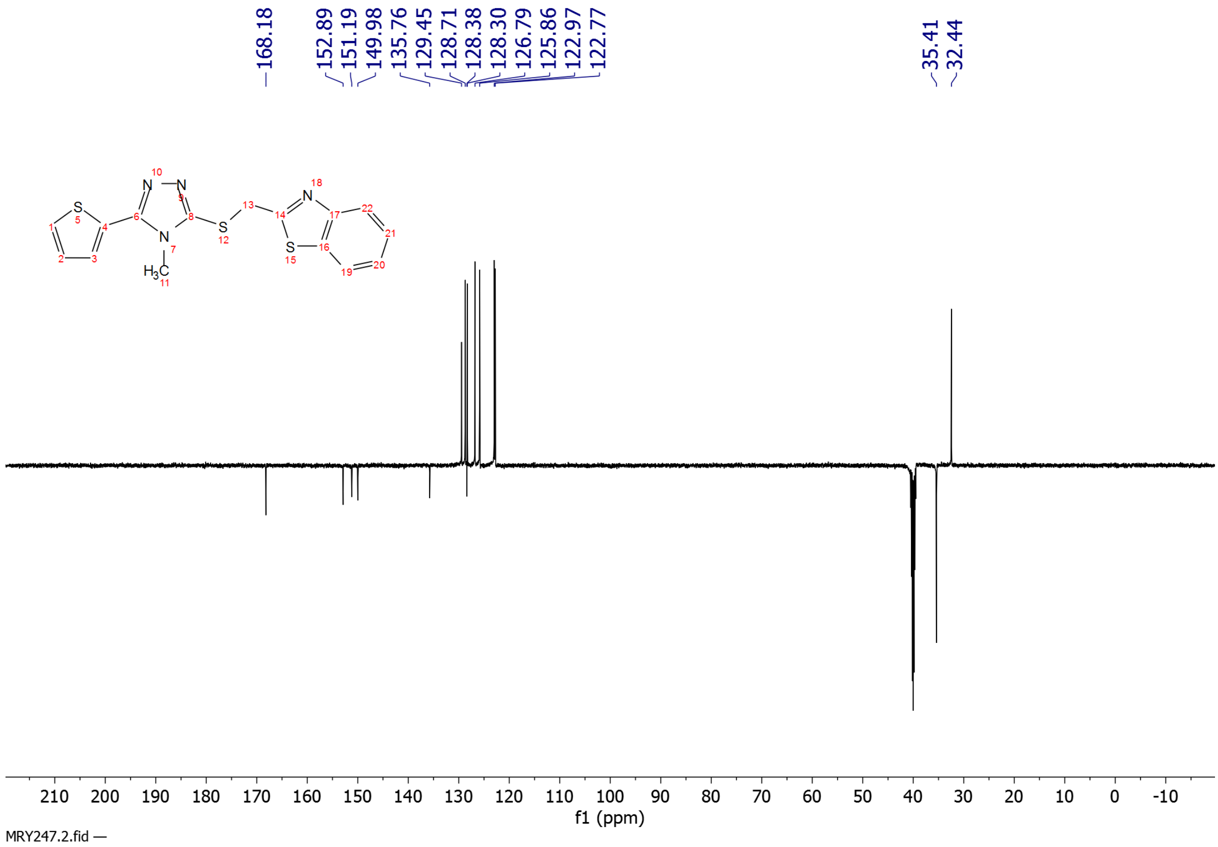

Figure S9. ^{1}H-NMR Spectrum of 6c

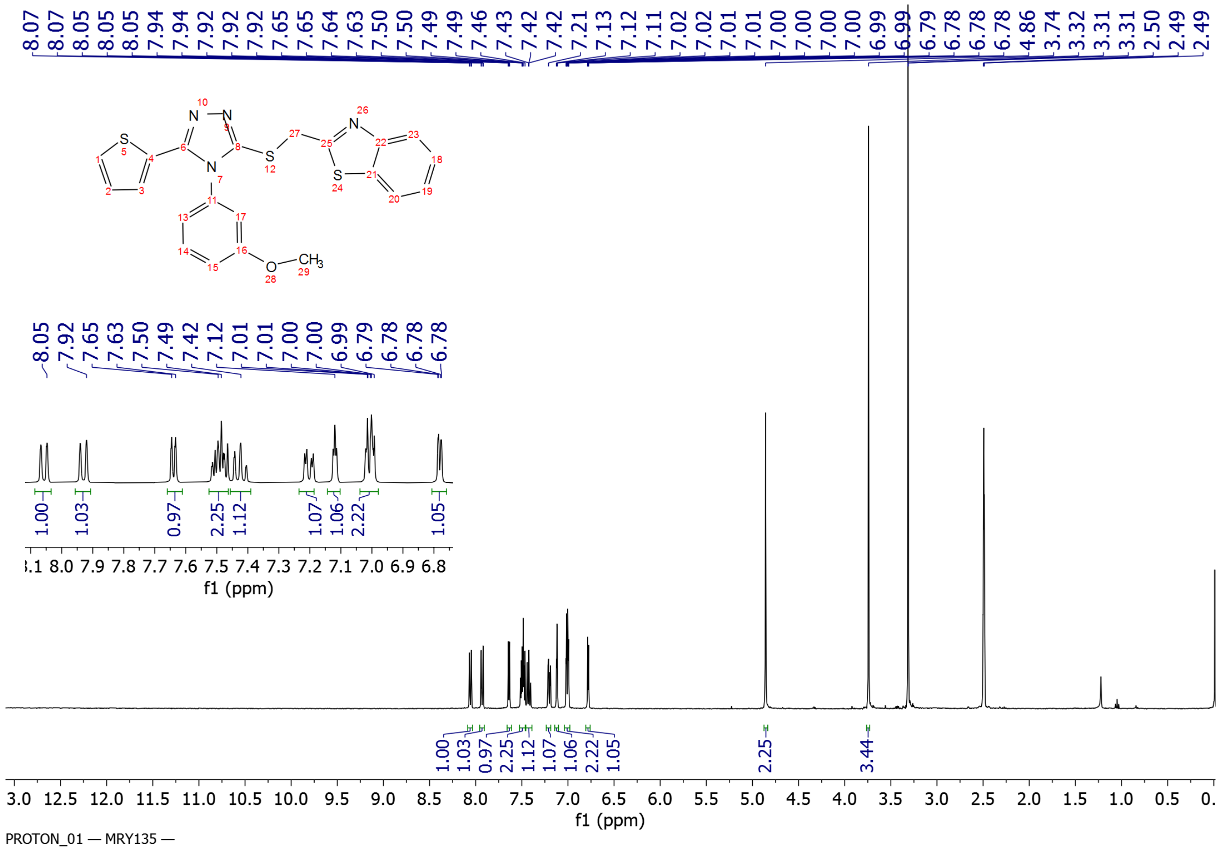

Figure S10. $^{13}C_{APT}$-NMR Spectrum of 6c

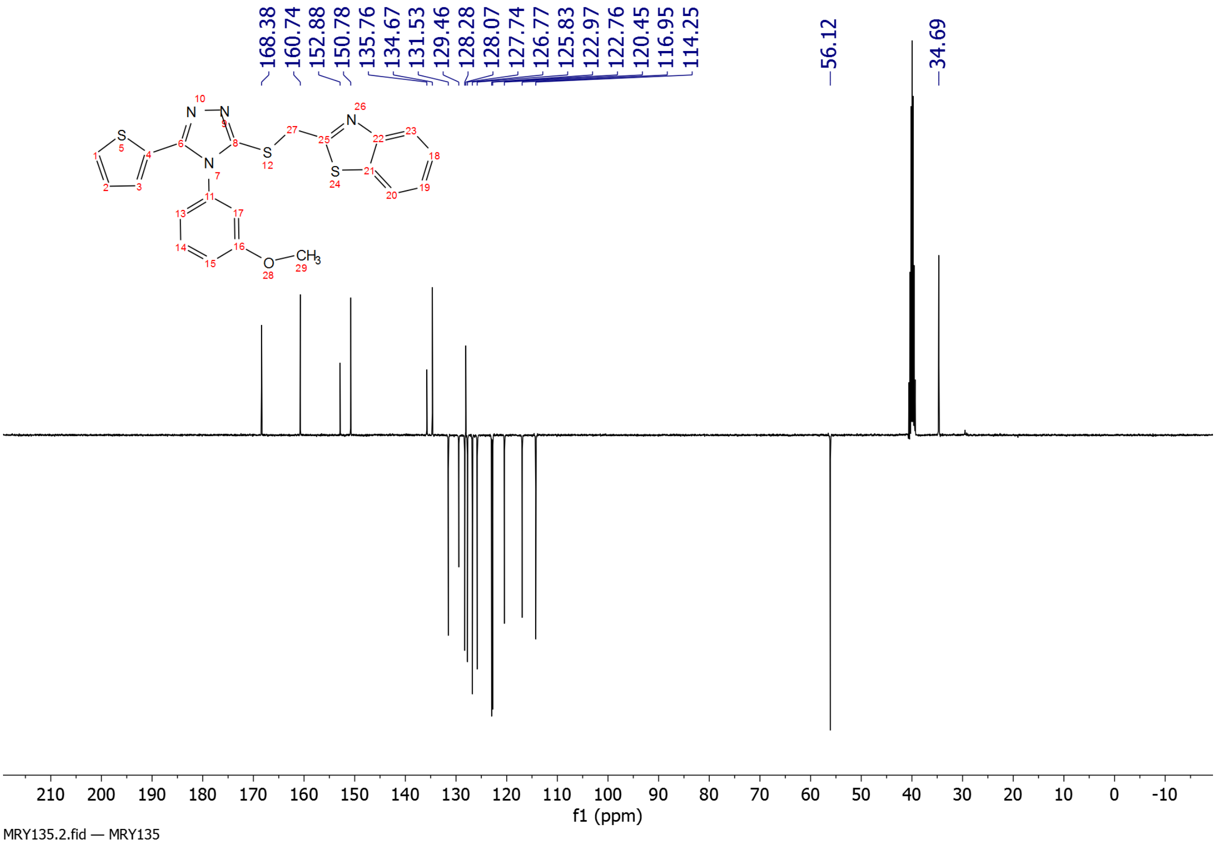

Figure S11. ^{1}H-NMR Spectrum of 6d

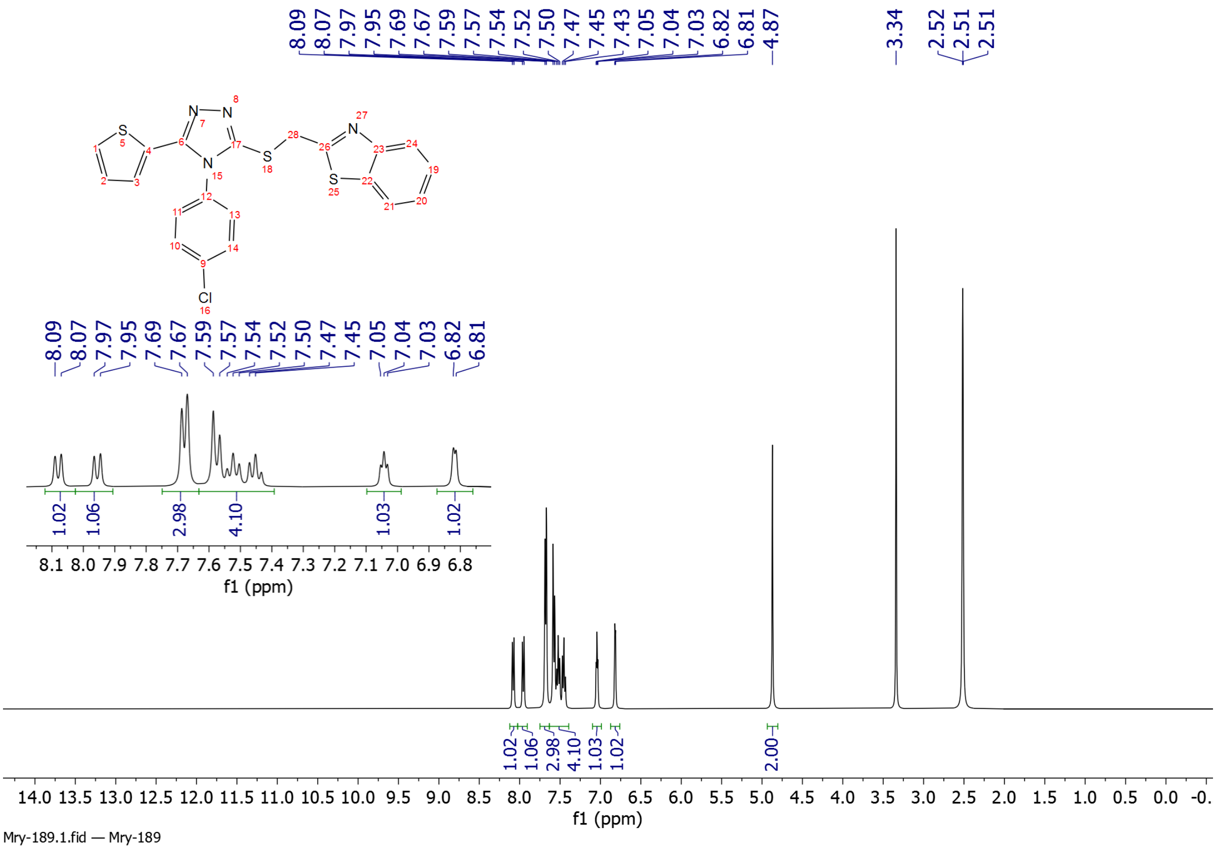

Mry-189.1.fid — Mry-189

Figure S12. $^{13}C_{APT}$-NMR Spectrum of 6d

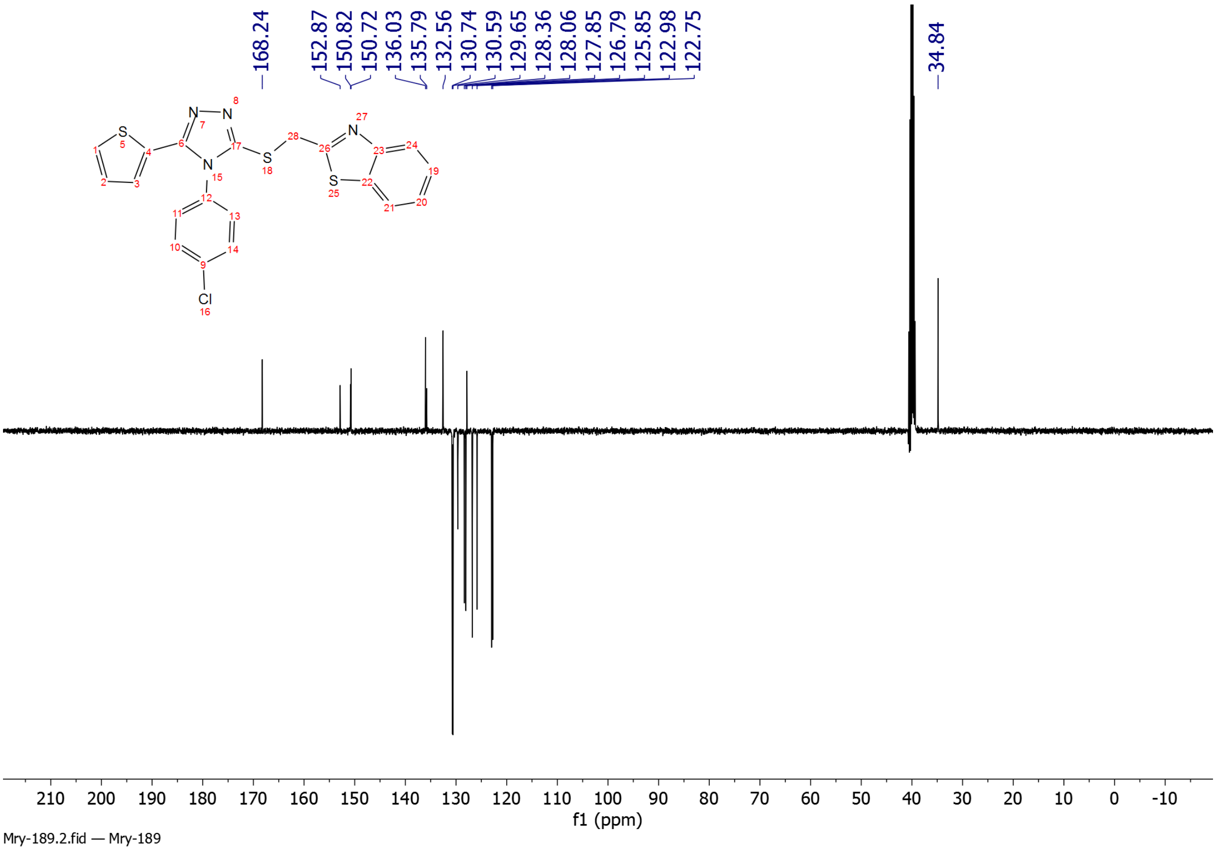

Mry-189.2.fid — Mry-189

Figure S13. ^{1}H-NMR Spectrum of 6e

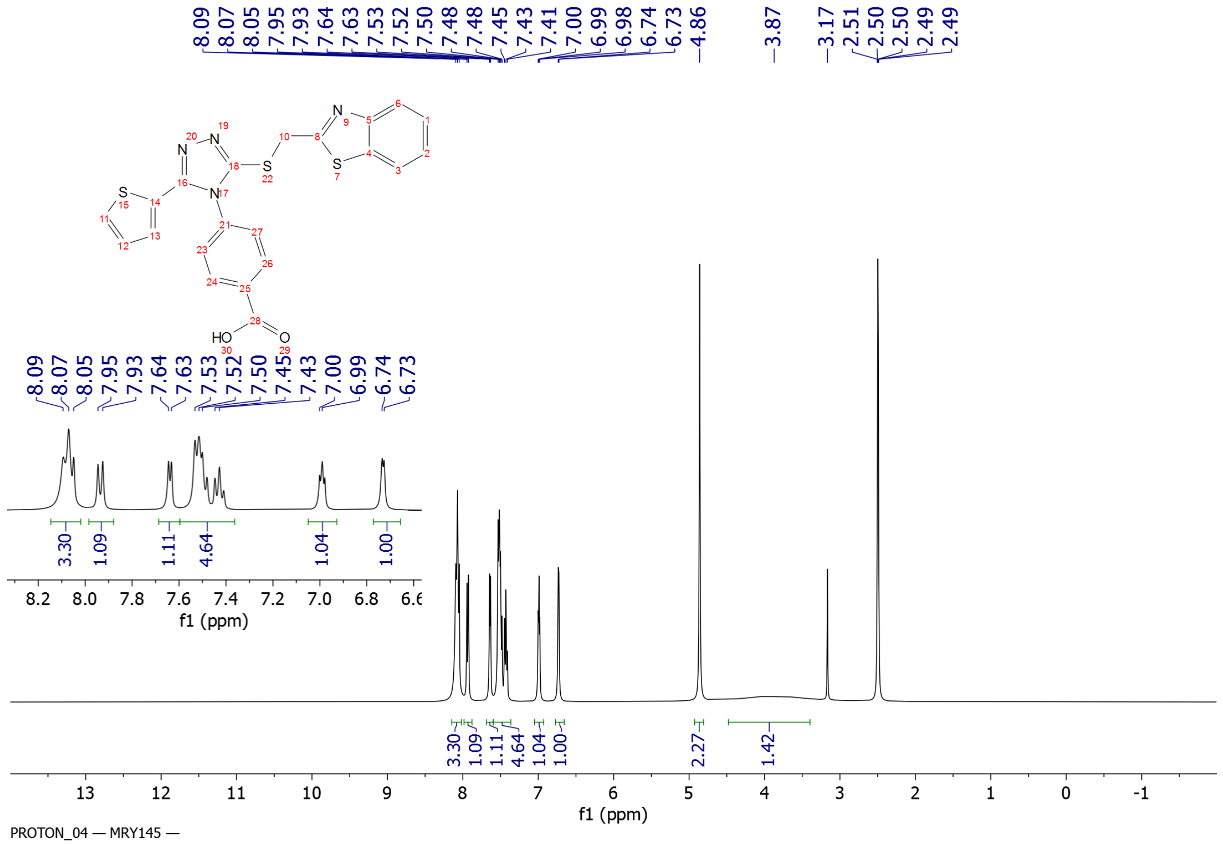

Figure S14. $^{13}C_{APT}$-NMR Spectrum of 6e

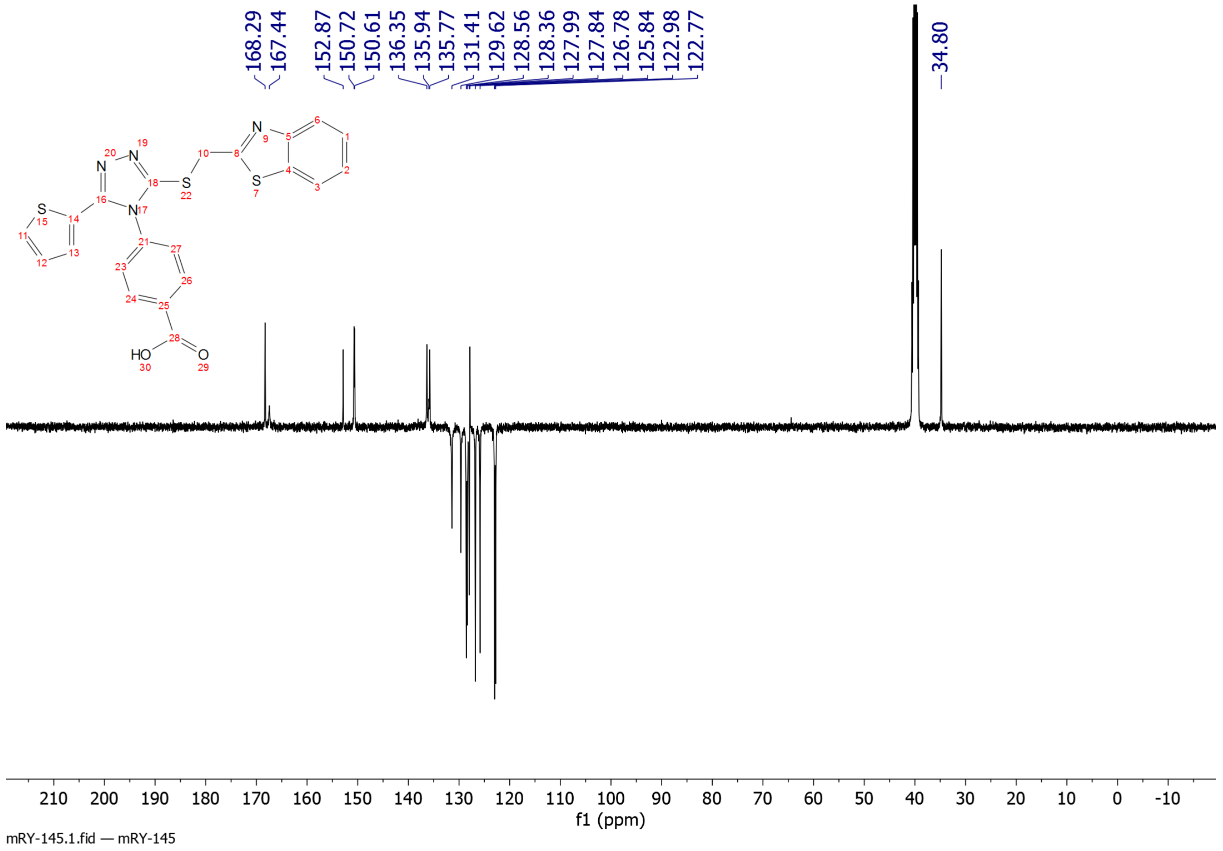

Figure S15. ^{1}H-NMR Spectrum of 6f

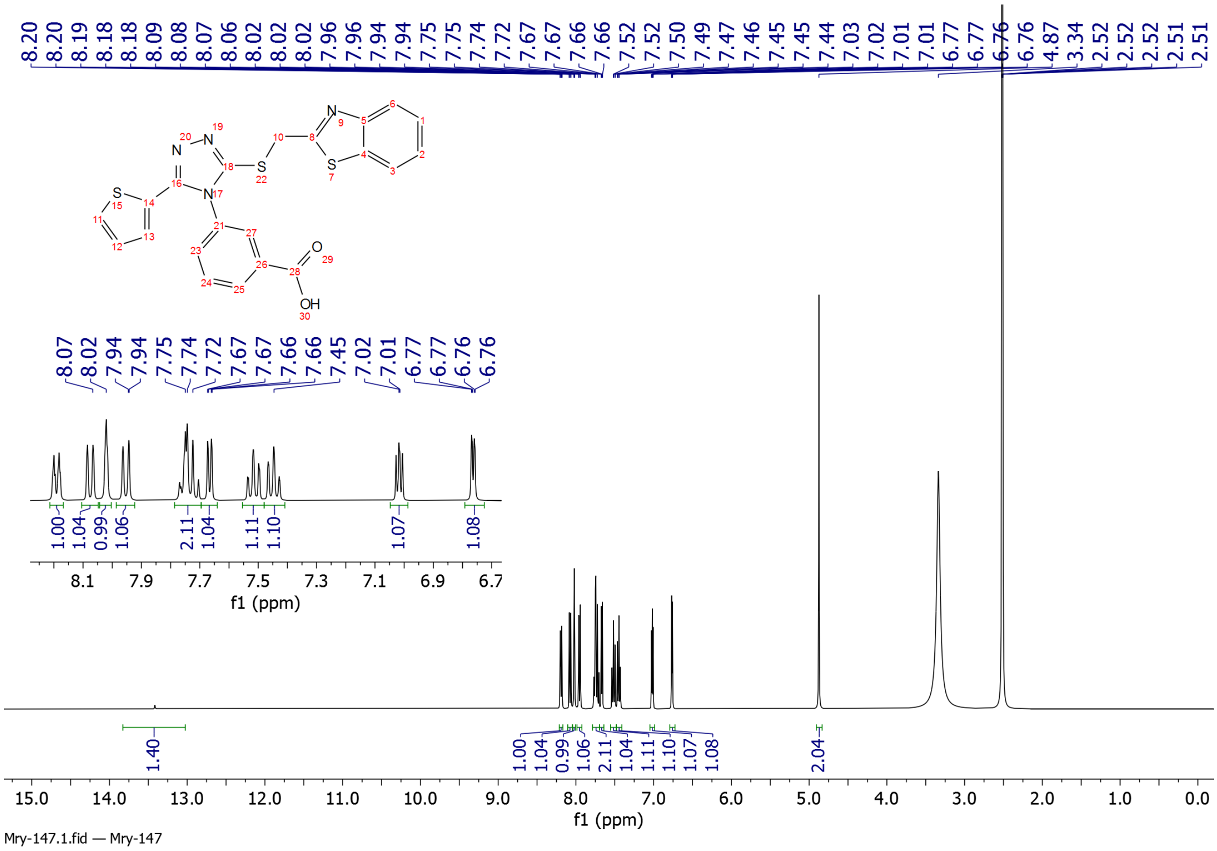

Mry-147.1.fid — Mry-147

Figure S16. ^{13}C$_{APT}$-NMR Spectrum of 6f

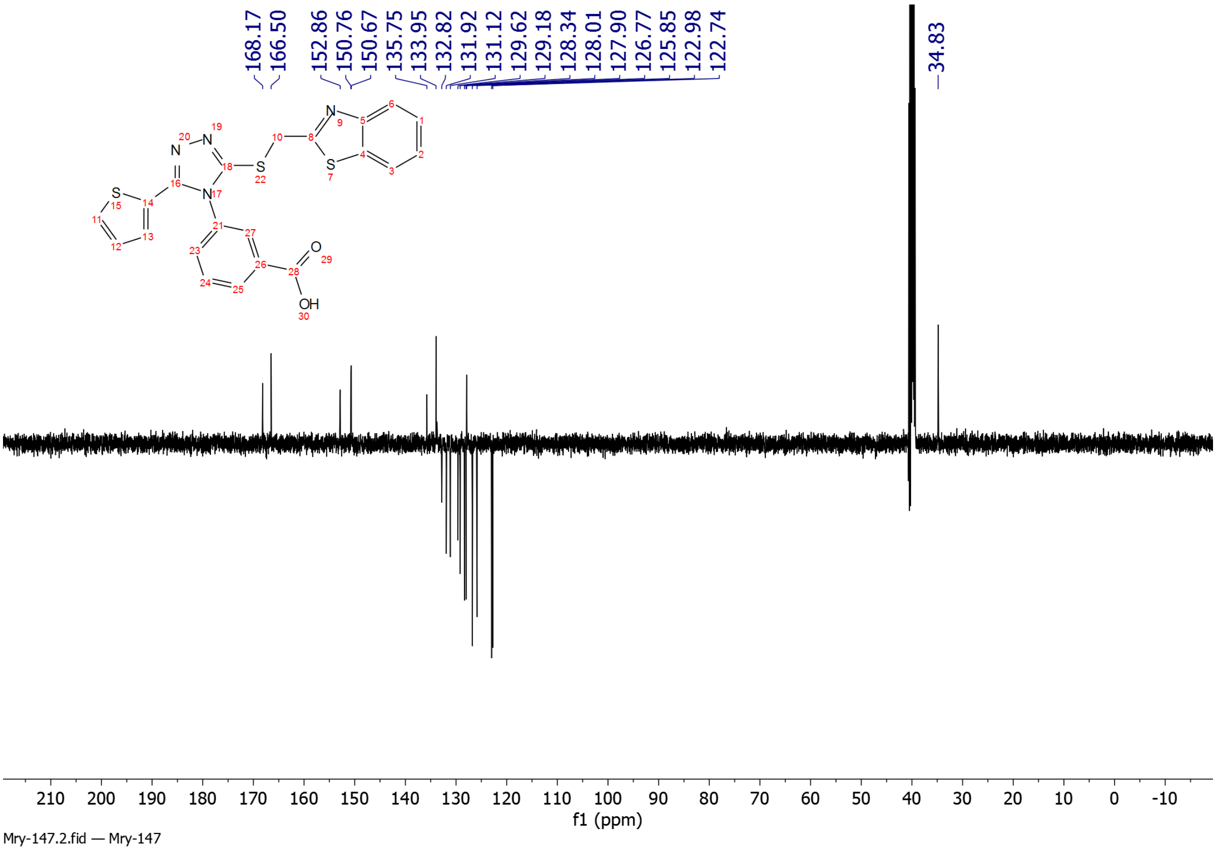

Mry-147.2.fid — Mry-147

Figure S17. ^{1}H-NMR Spectrum of 6g

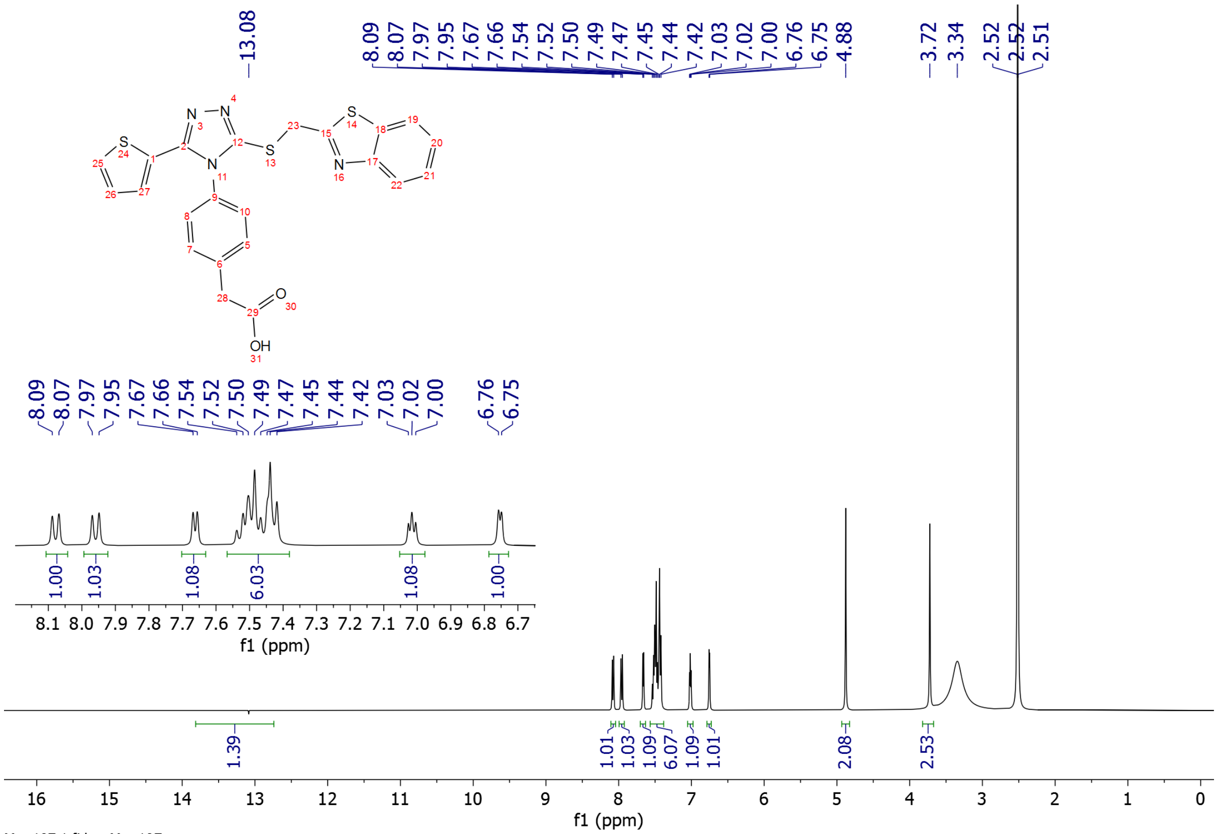

Mry-197.1.fid — Mry-197

Figure S18. $^{13}C_{APT}$-NMR Spectrum of 6g

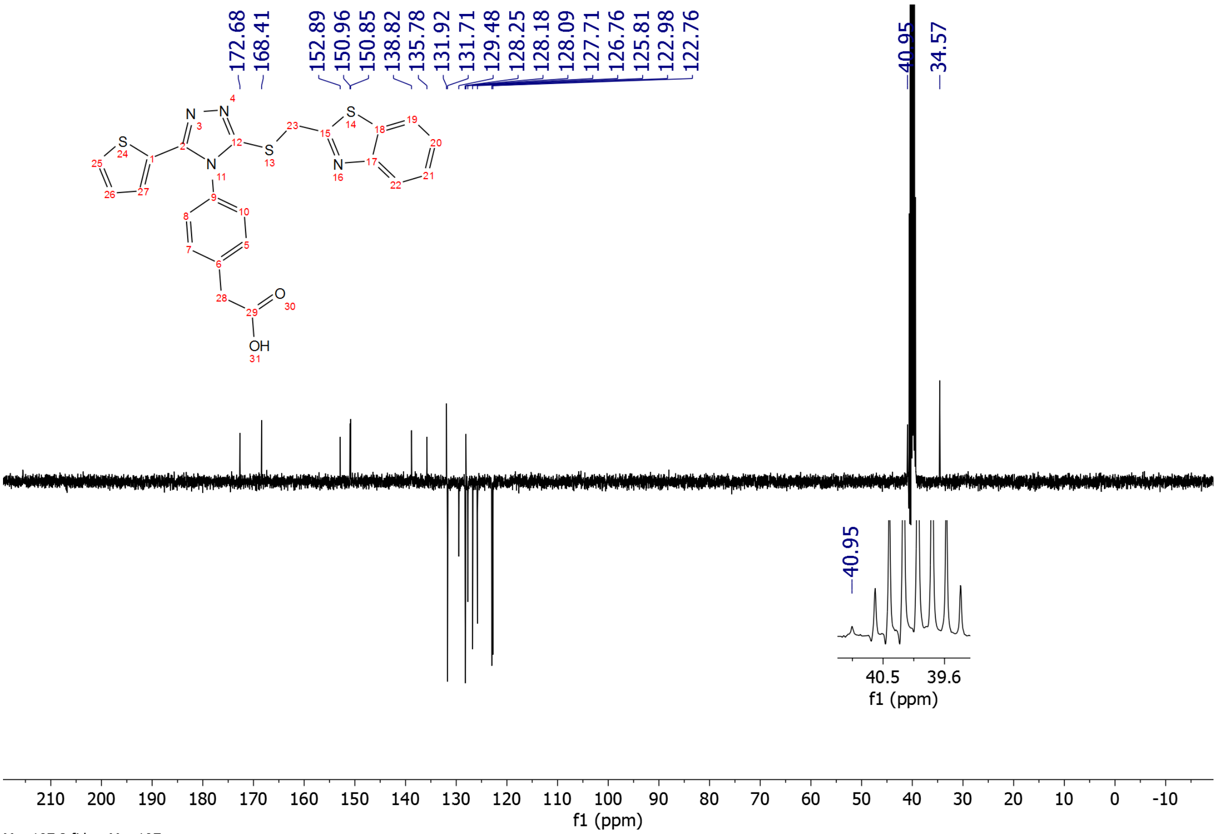

Mry-197.2.fid — Mry-197

Figure S19. ^{1}H-NMR Spectrum of 6h

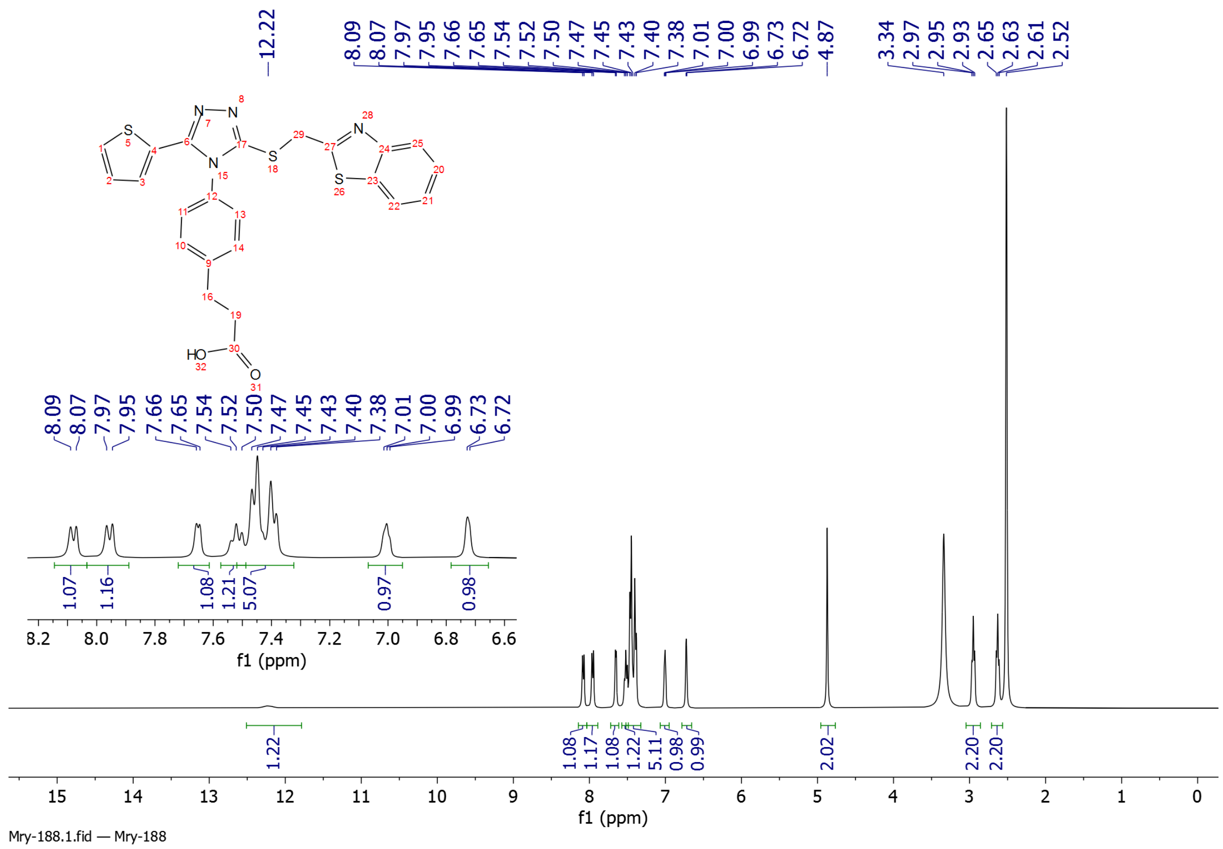

Figure S20. $^{13}C_{APT}$-NMR Spectrum of 6h

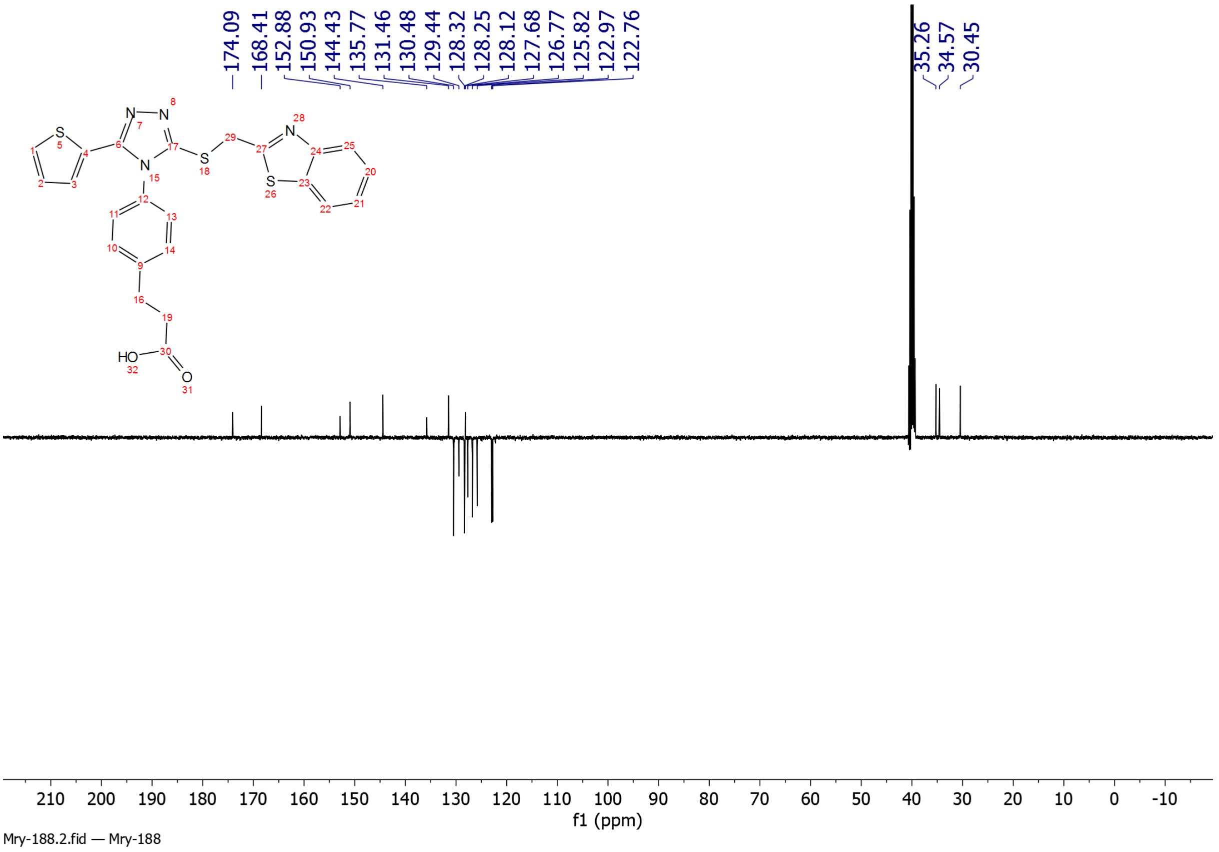

Figure S21. ^{1}H-NMR Spectrum of 6i

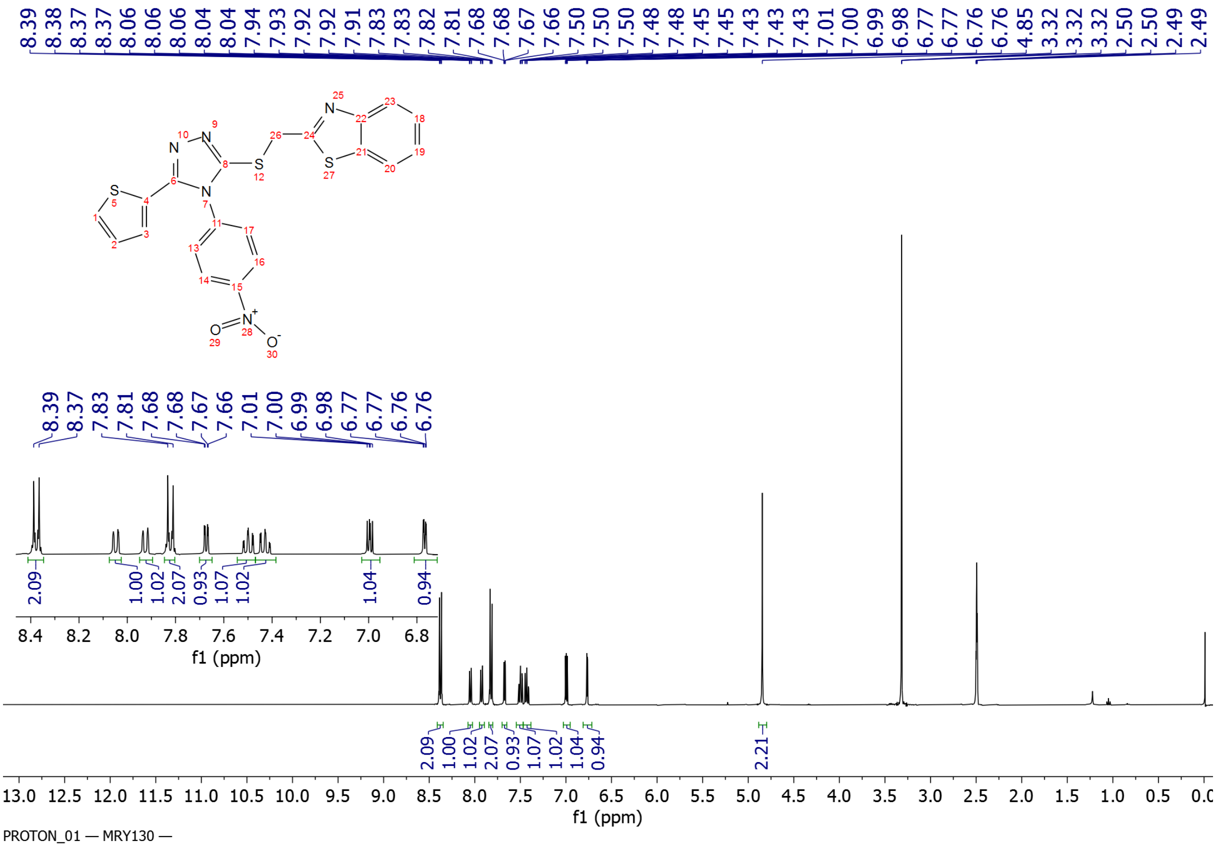

Figure S22. $^{13}C_{APT}$-NMR Spectrum of 6i

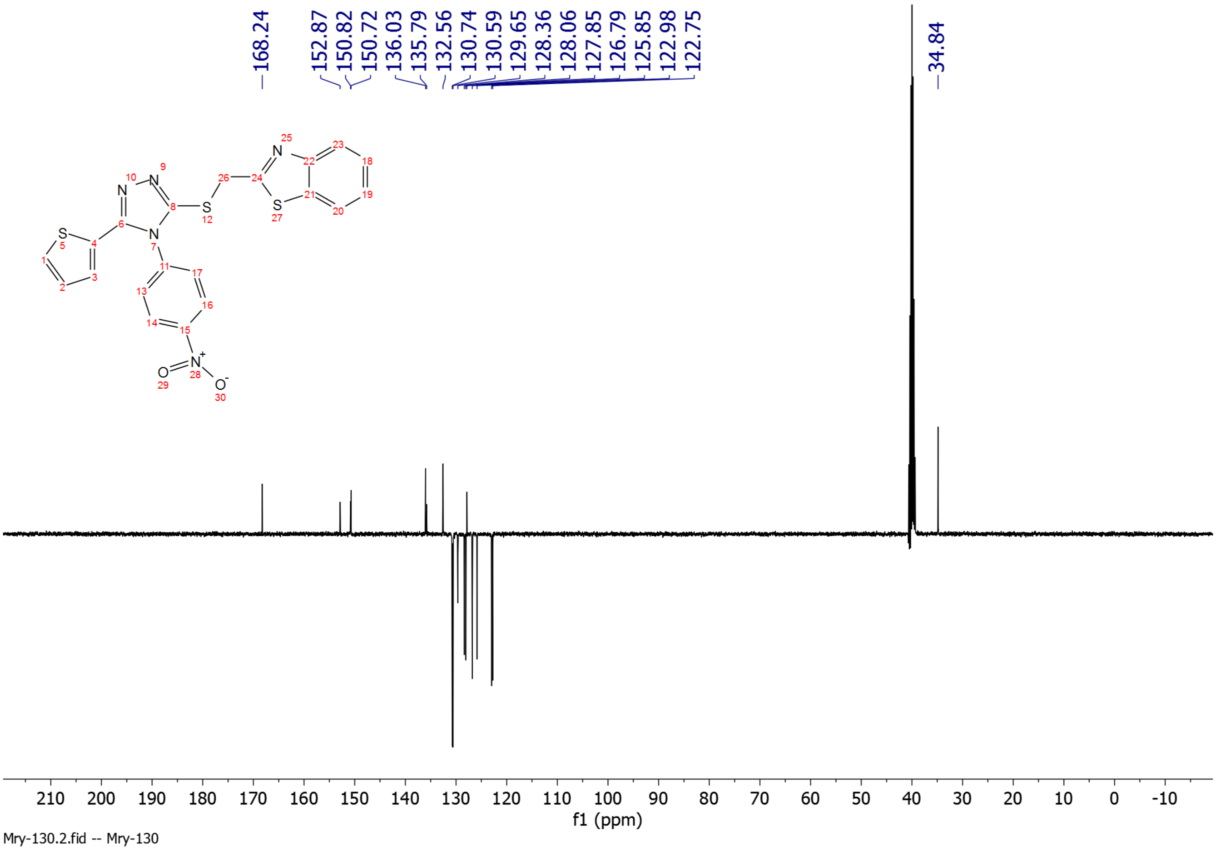

Figure S23. ^{1}H-NMR Spectrum of 6j

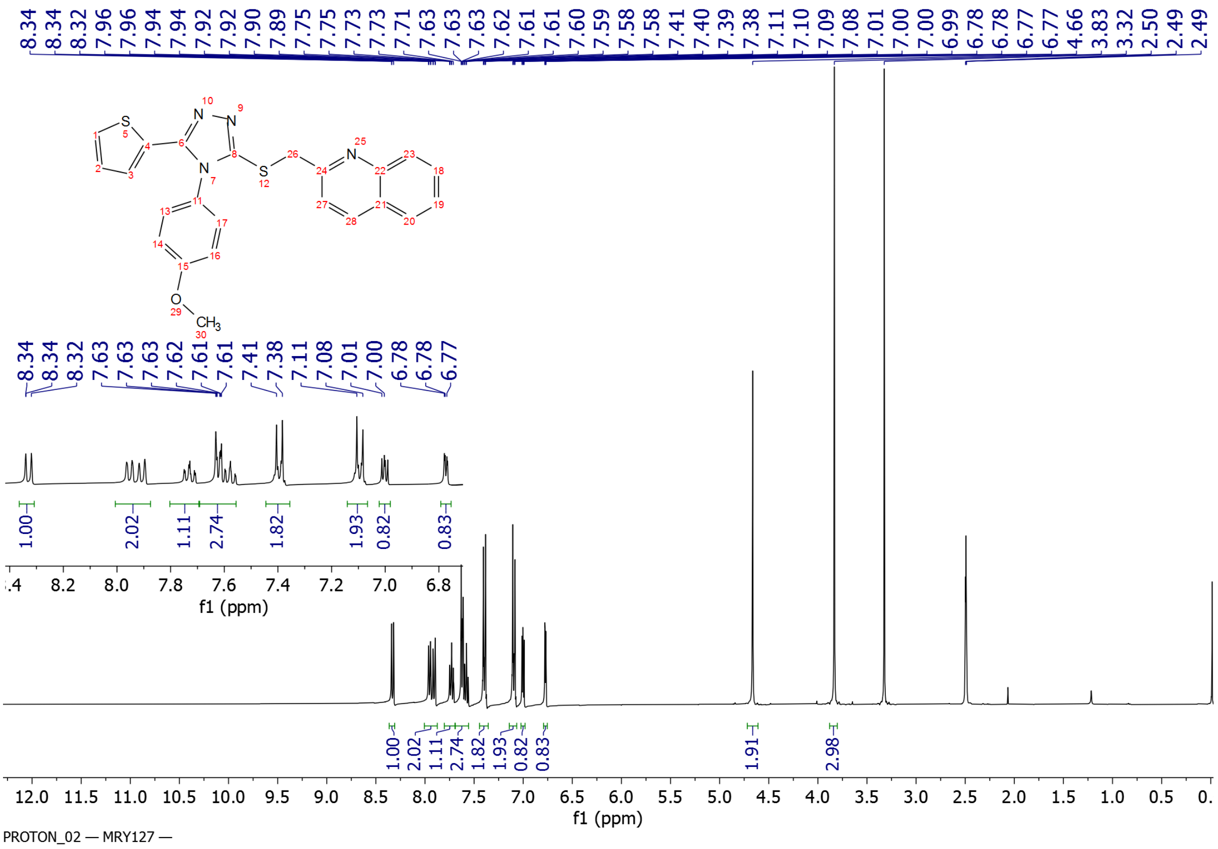

Figure S24. $^{13}C_{APT}$-NMR Spectrum of 6j

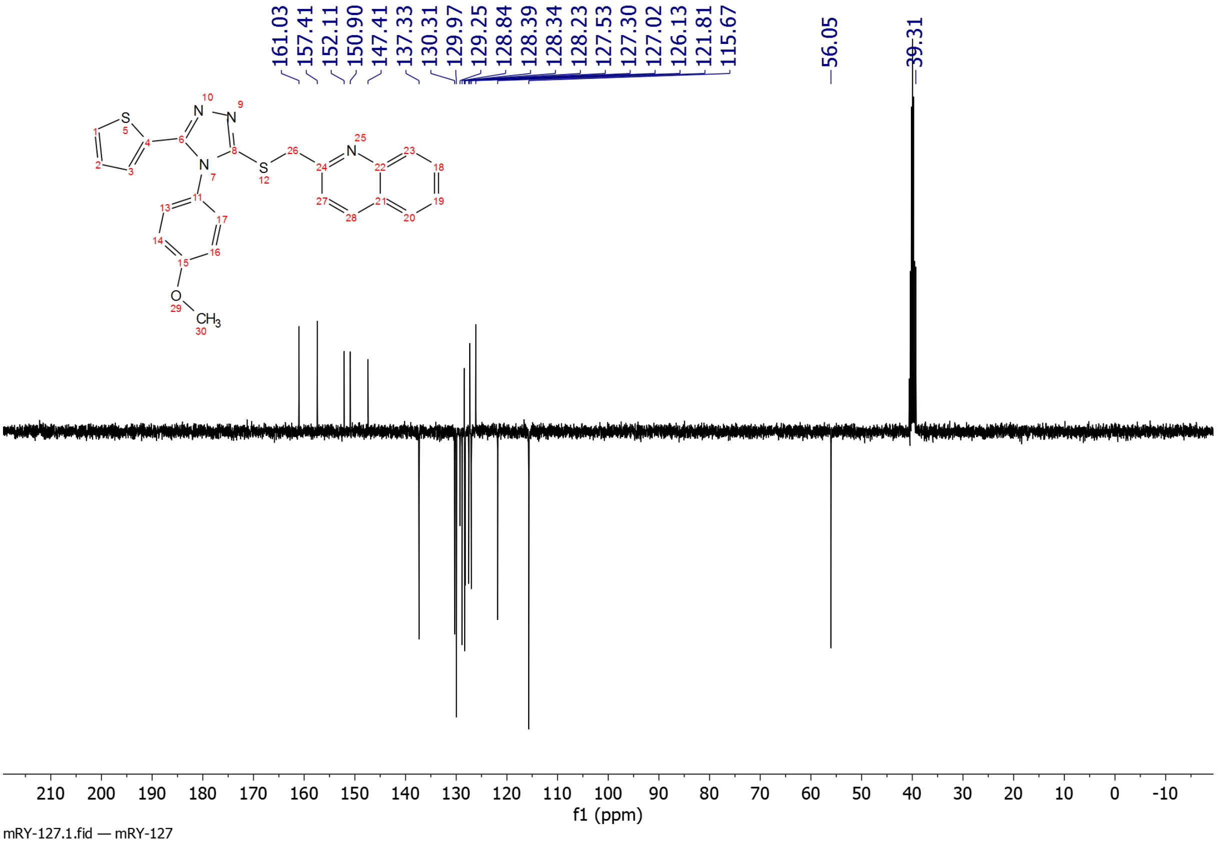

Figure S25. ^{1}H-NMR Spectrum of 6k

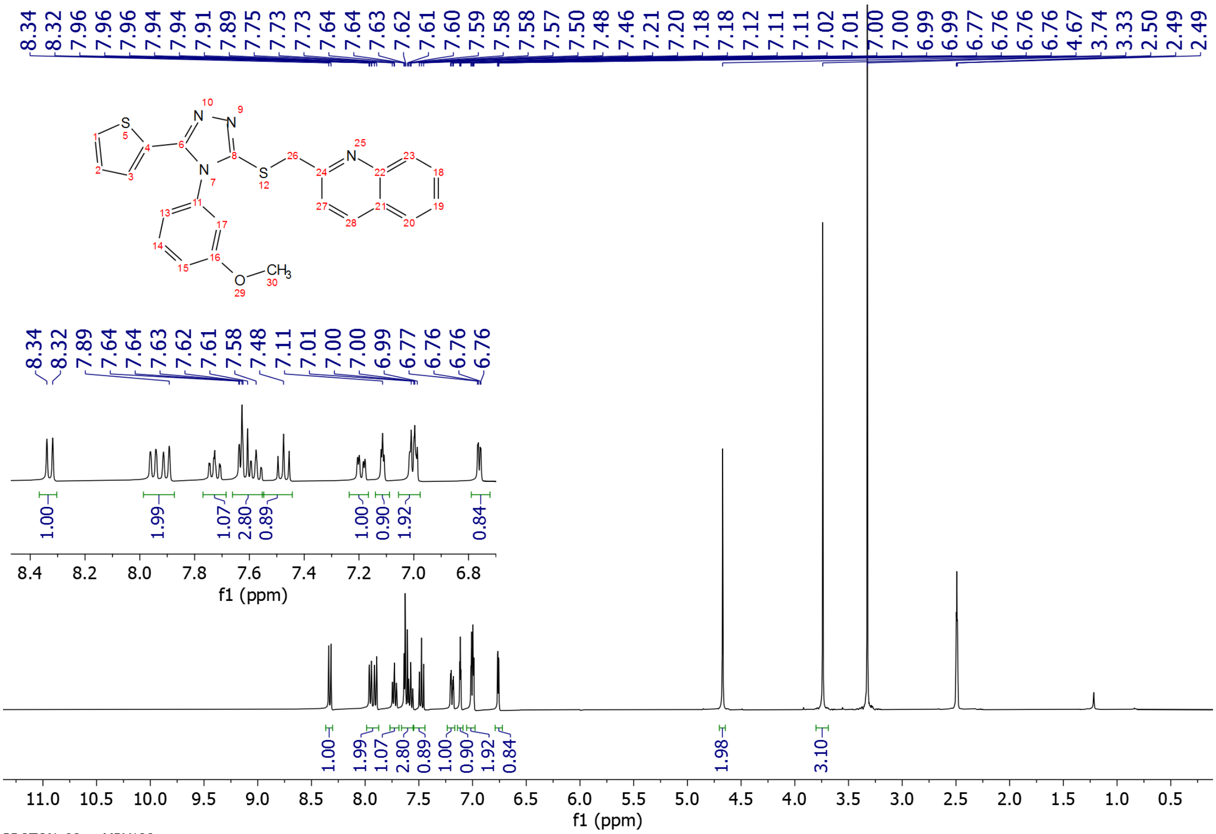

PROTON_02 — MRY128 —

Figure S26. $^{13}C_{APT}$-NMR Spectrum of 6k

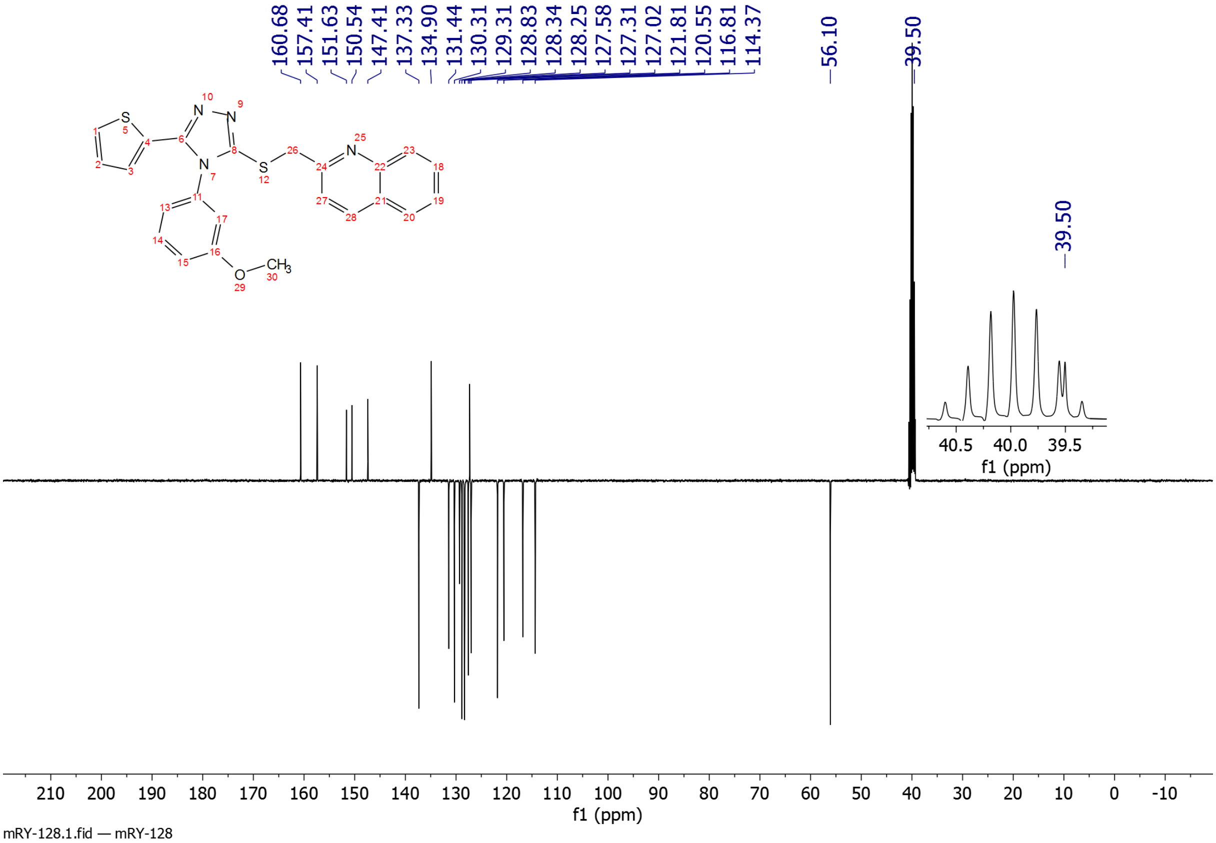

mRY-128.1.fid — mRY-128

Figure S27. ¹H-NMR Spectrum of 6l

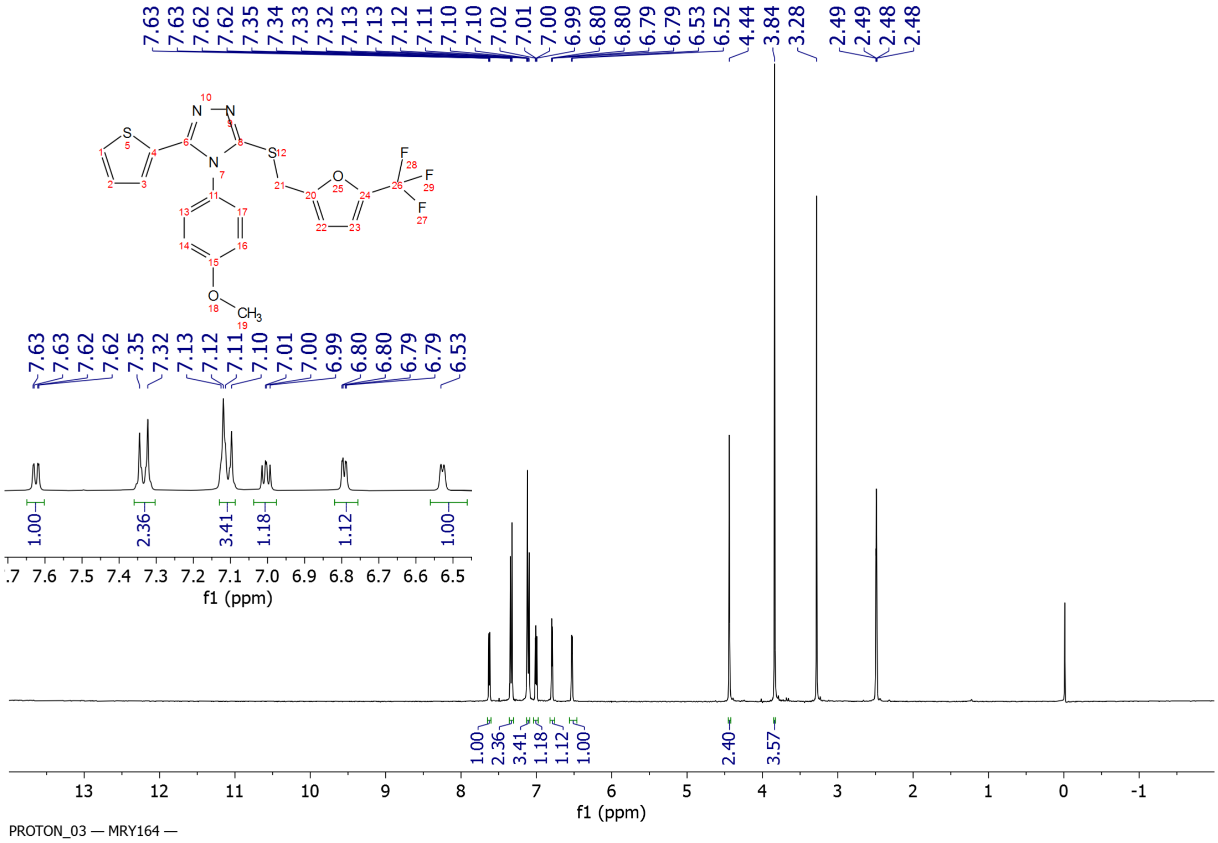

Figure S28. $^{13}C_{APT}$-NMR Spectrum of 6l

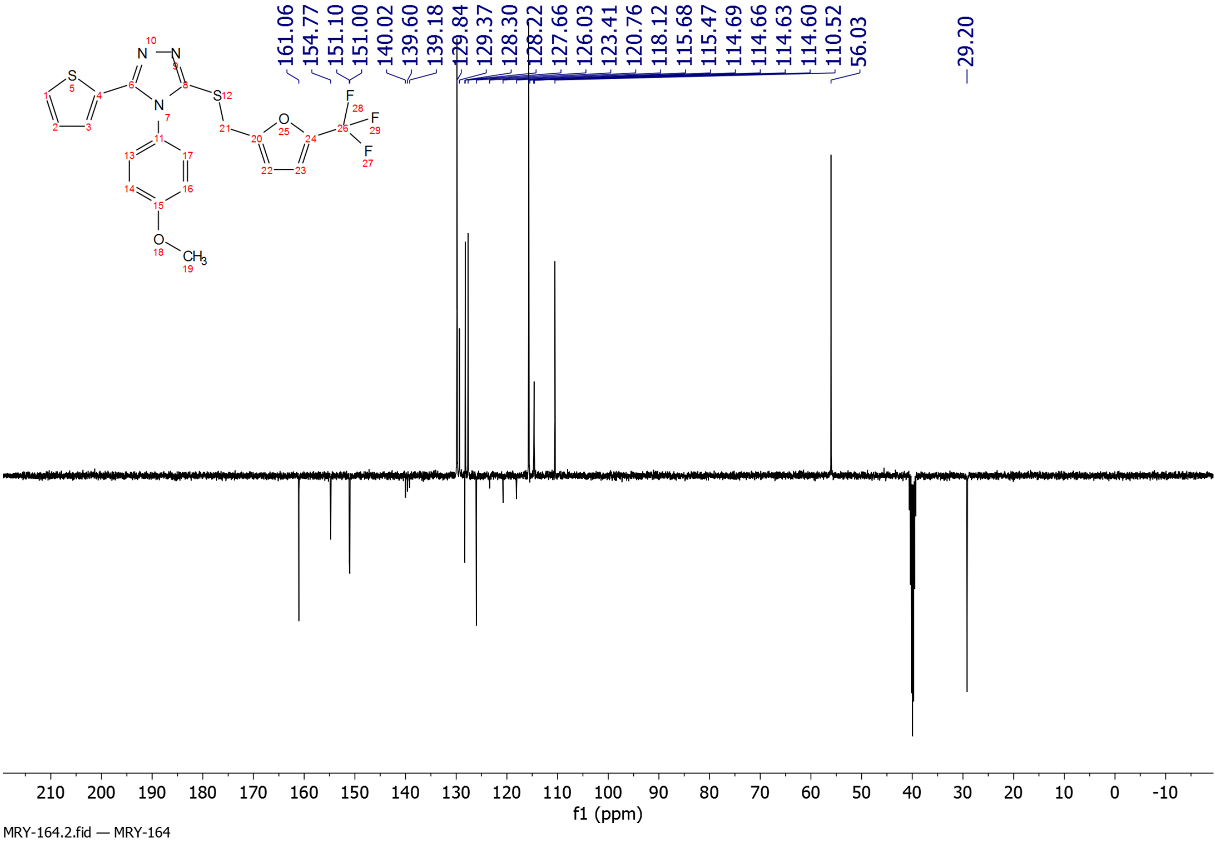

Figure S29. ^{1}H-NMR Spectrum of 6m

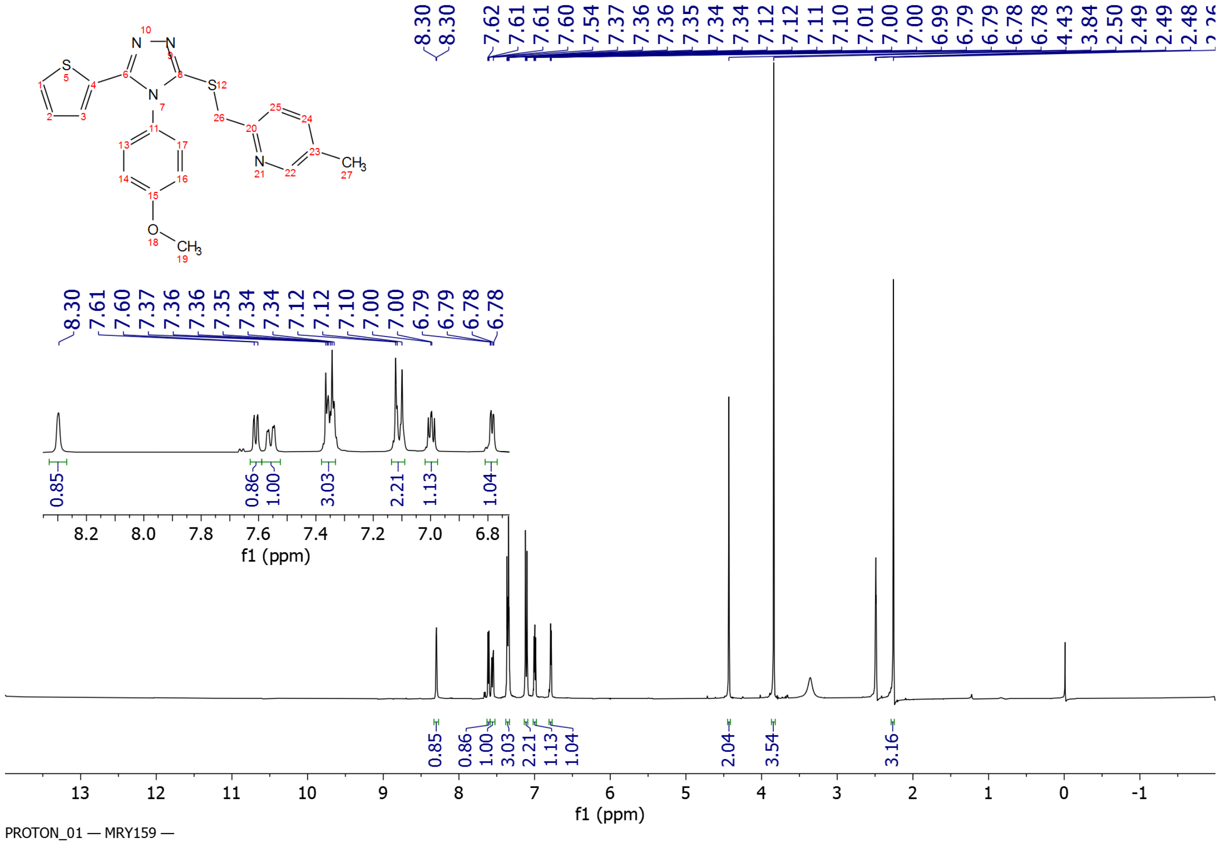

Figure S30. $^{13}C_{APT}$-NMR Spectrum of 6m

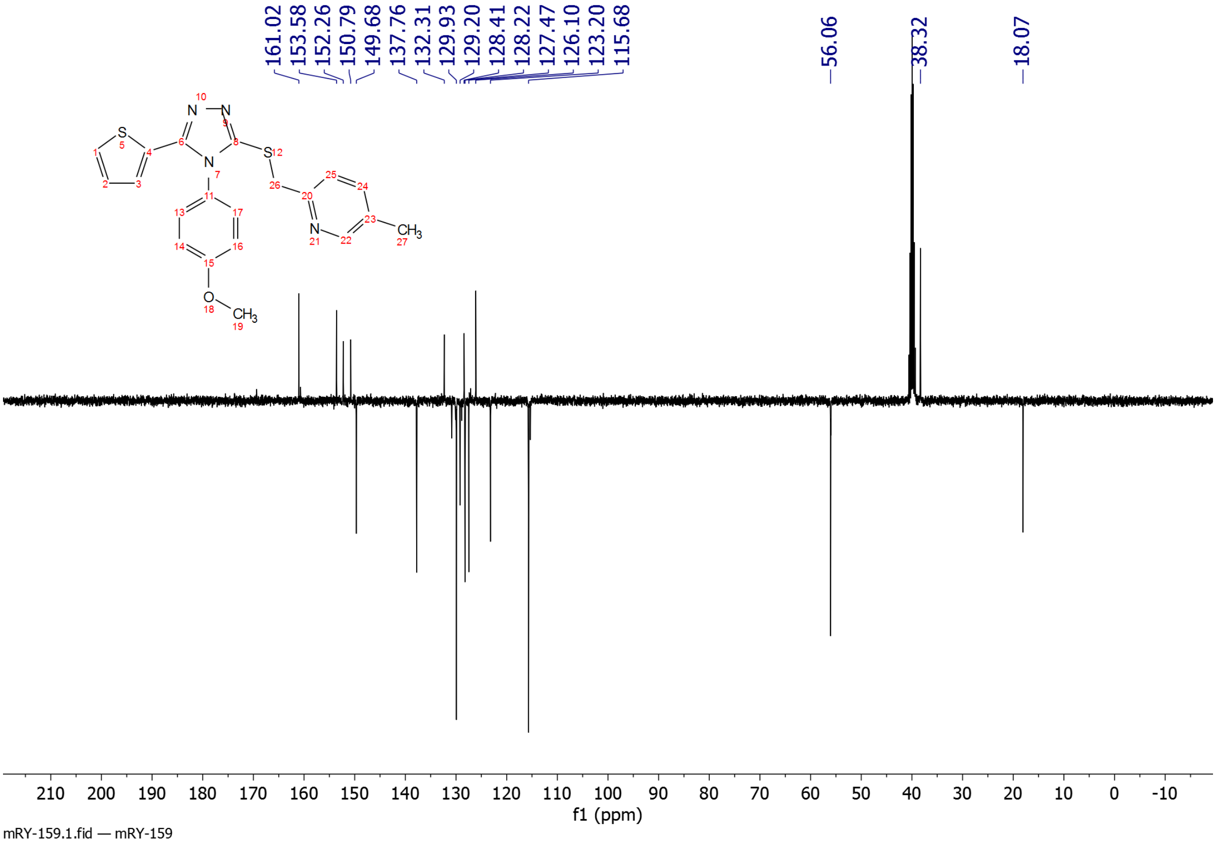

Figure S31. ^{1}H-NMR Spectrum of 6n

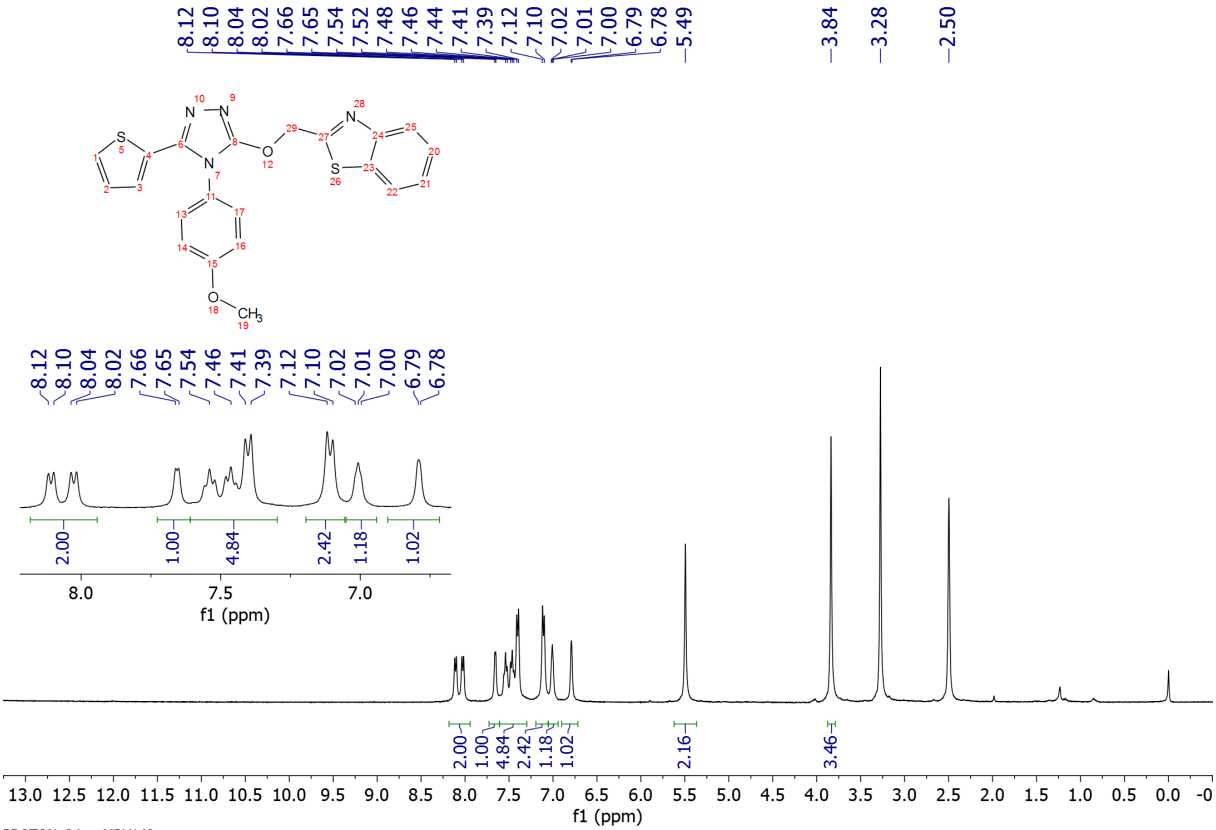

PROTON_04 — MRY149 —

Figure S32. $^{13}C_{APT}$-NMR Spectrum of 6n

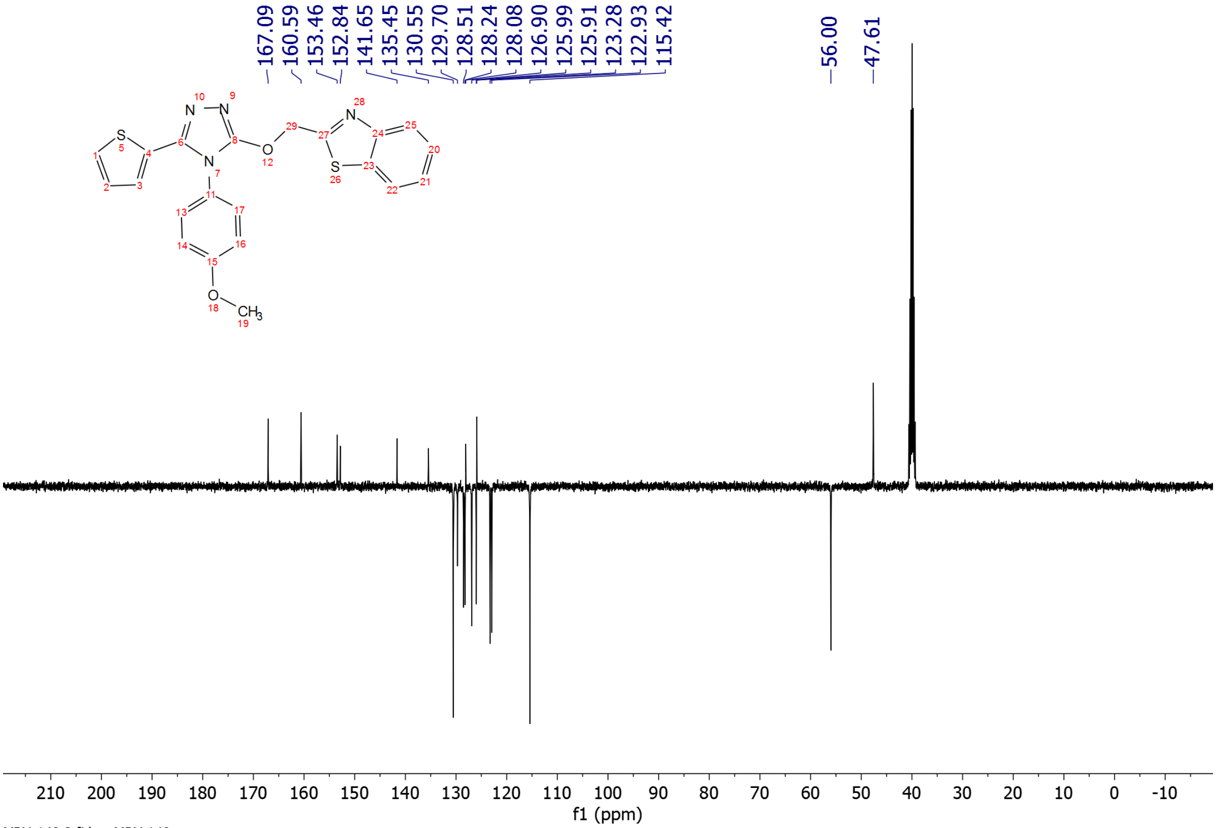

MRY-149.2.fid — MRY-149

Figure S33. ^{1}H-NMR Spectrum of 6o

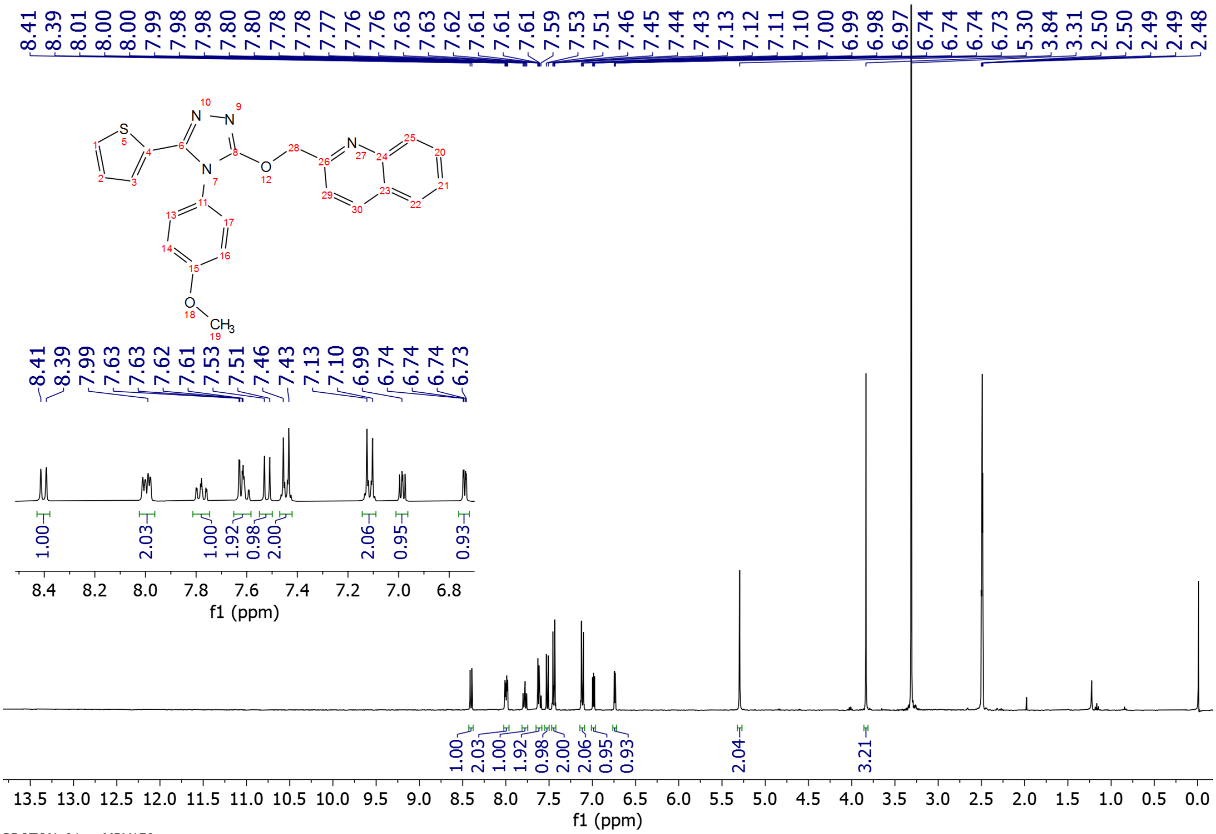

PROTON_01 — MRY150 —

Figure S34. $^{13}C_{APT}$-NMR Spectrum of 6o

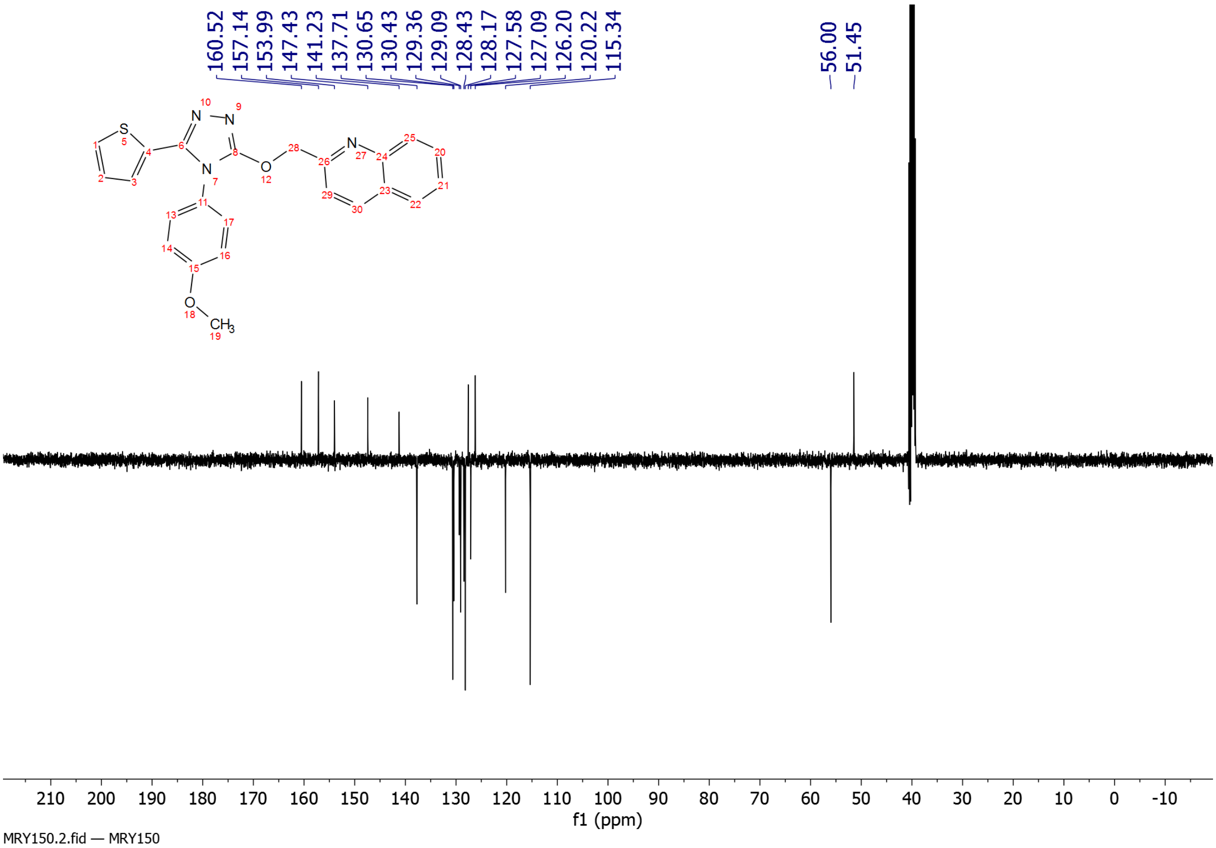

MRY150.2.fid — MRY150

Figure S35. ^{1}H-NMR Spectrum of 6p

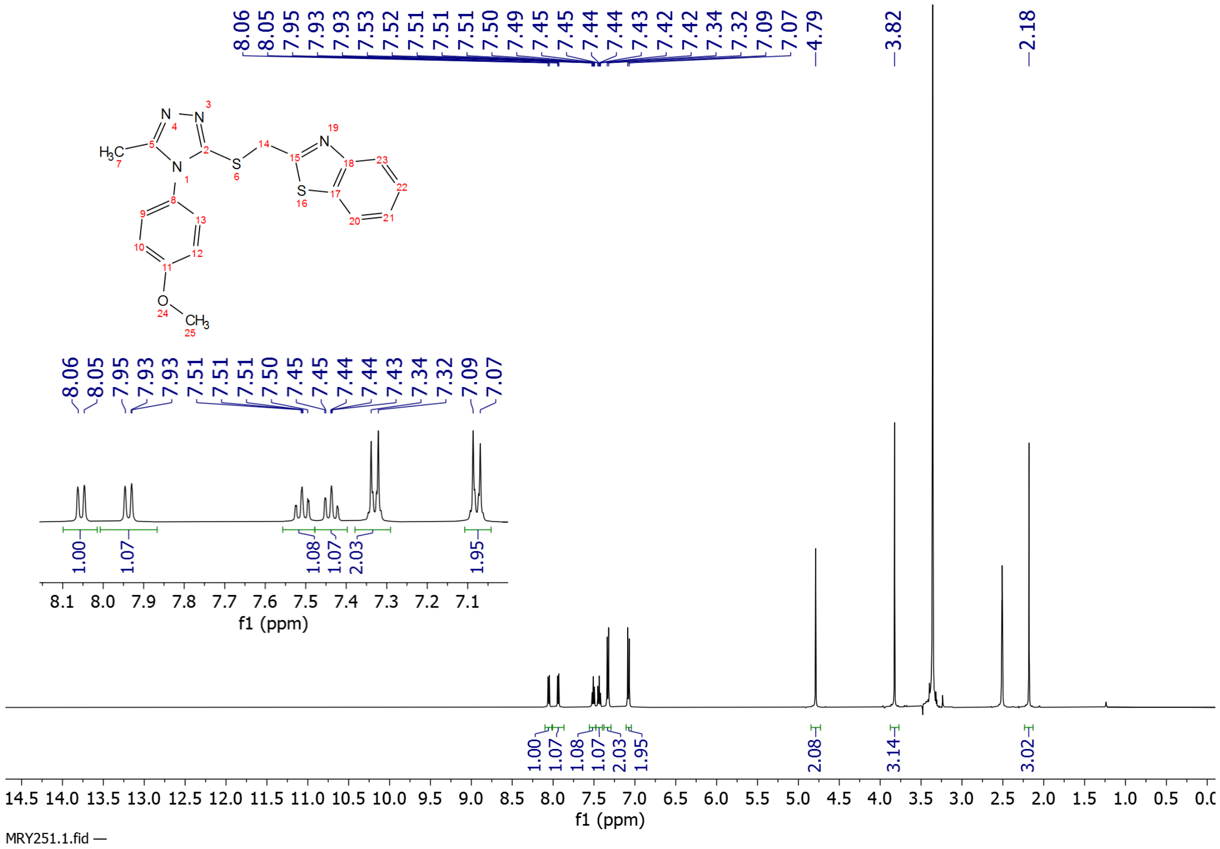

Figure S36. ^{13}C$_{APT}$-NMR Spectrum of 6p

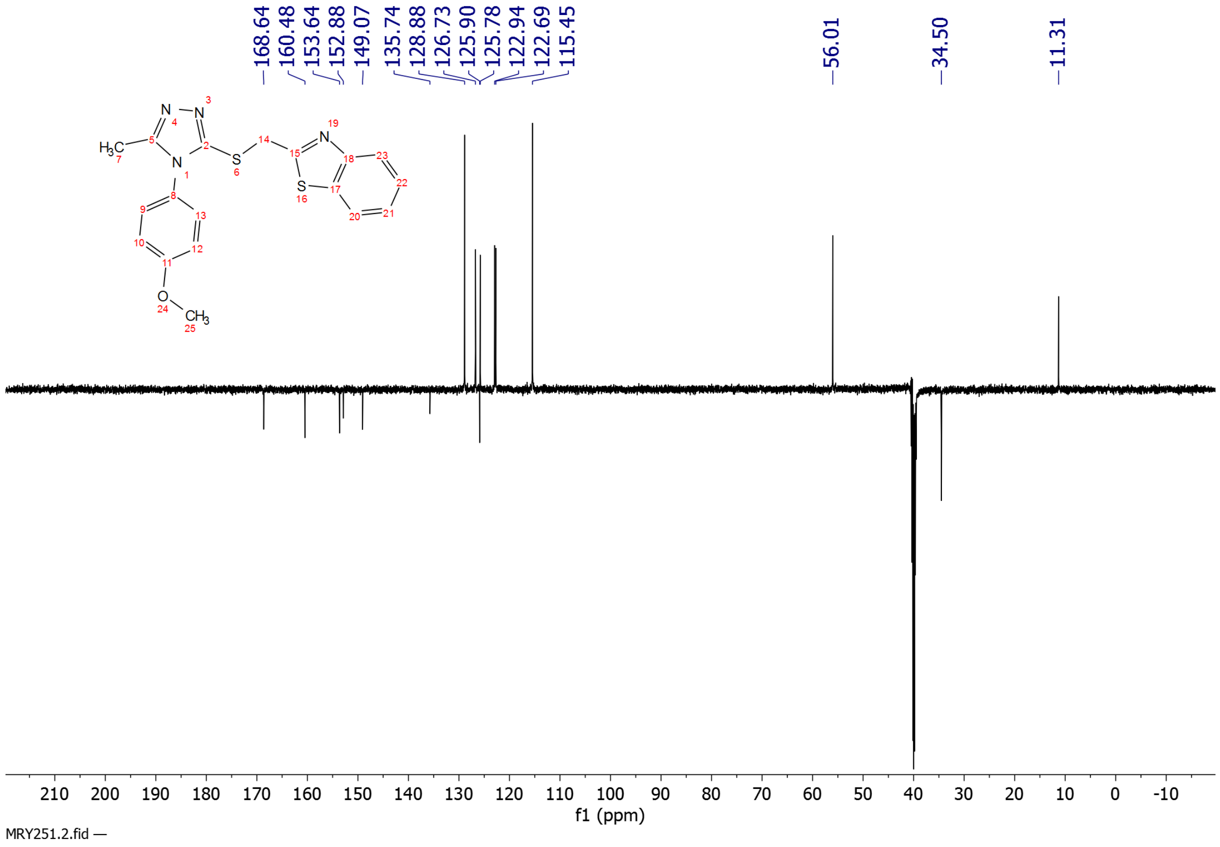

Figure S37. ^{1}H-NMR Spectrum of 6q

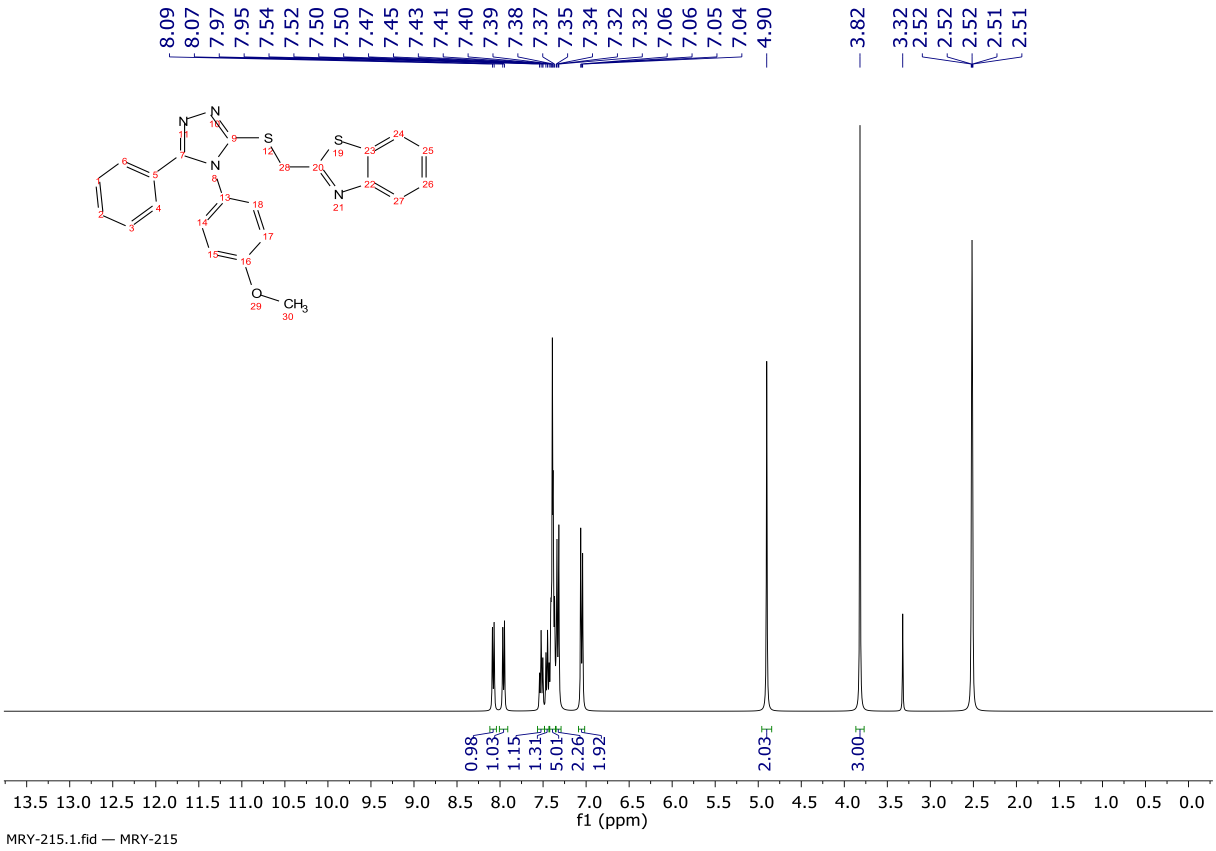

Figure S38. $^{13}C_{APT}$-NMR Spectrum of 6q

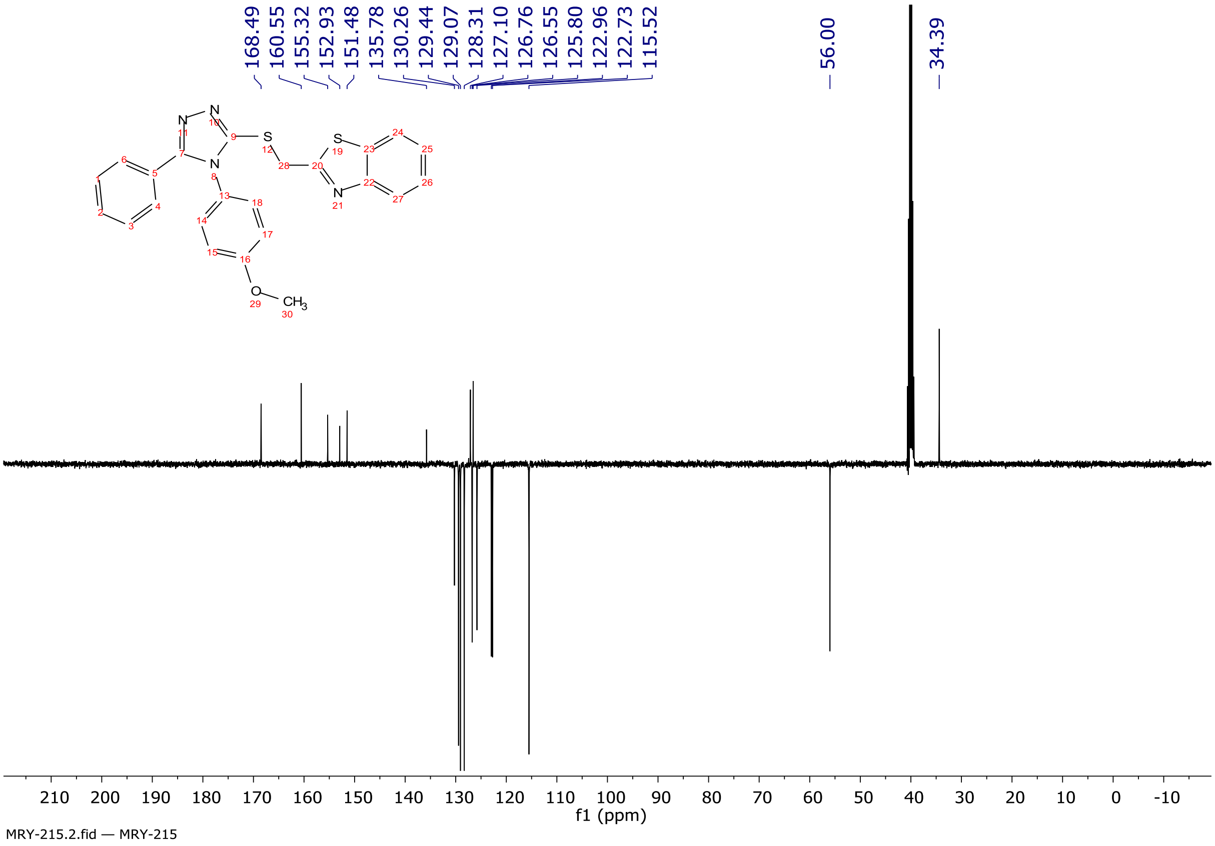

Figure S39. ^{1}H-NMR Spectrum of 6r

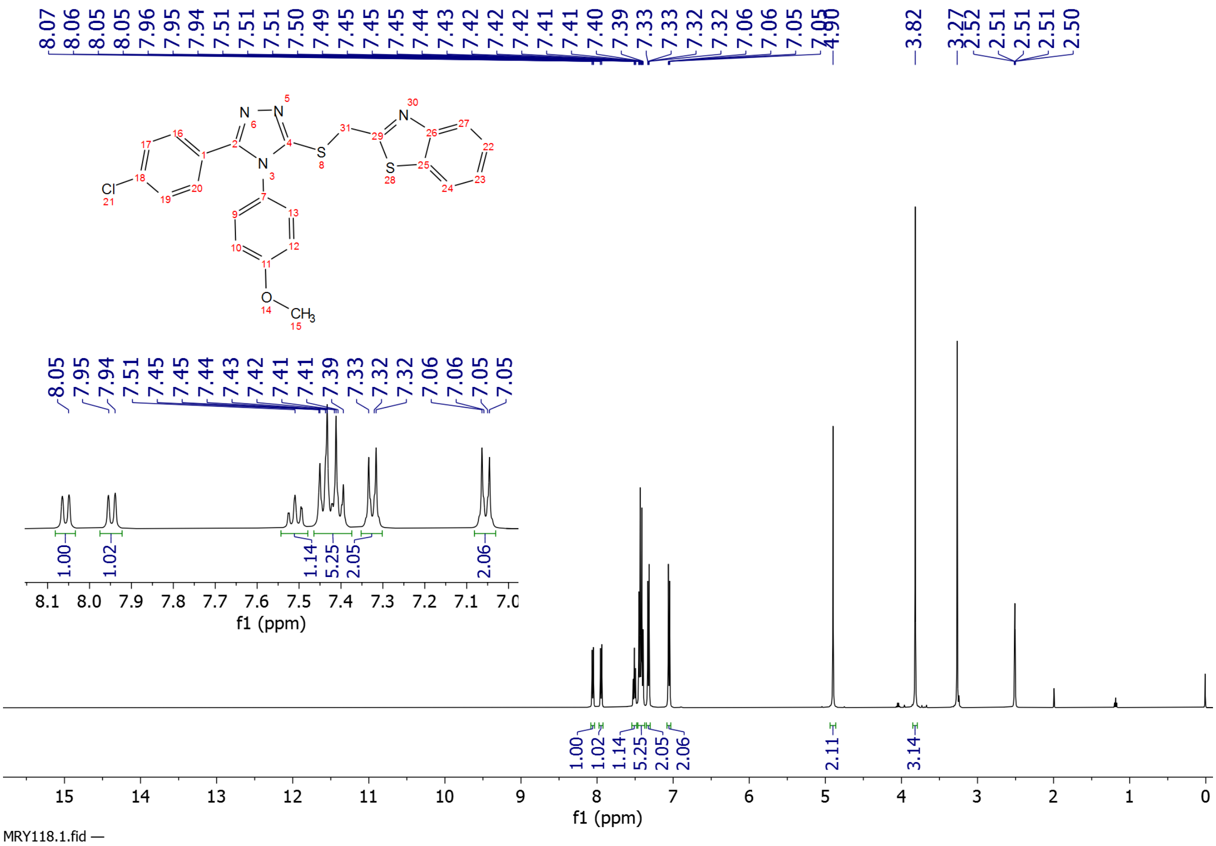

Figure S40. $^{13}C_{APT}$-NMR Spectrum of 6r

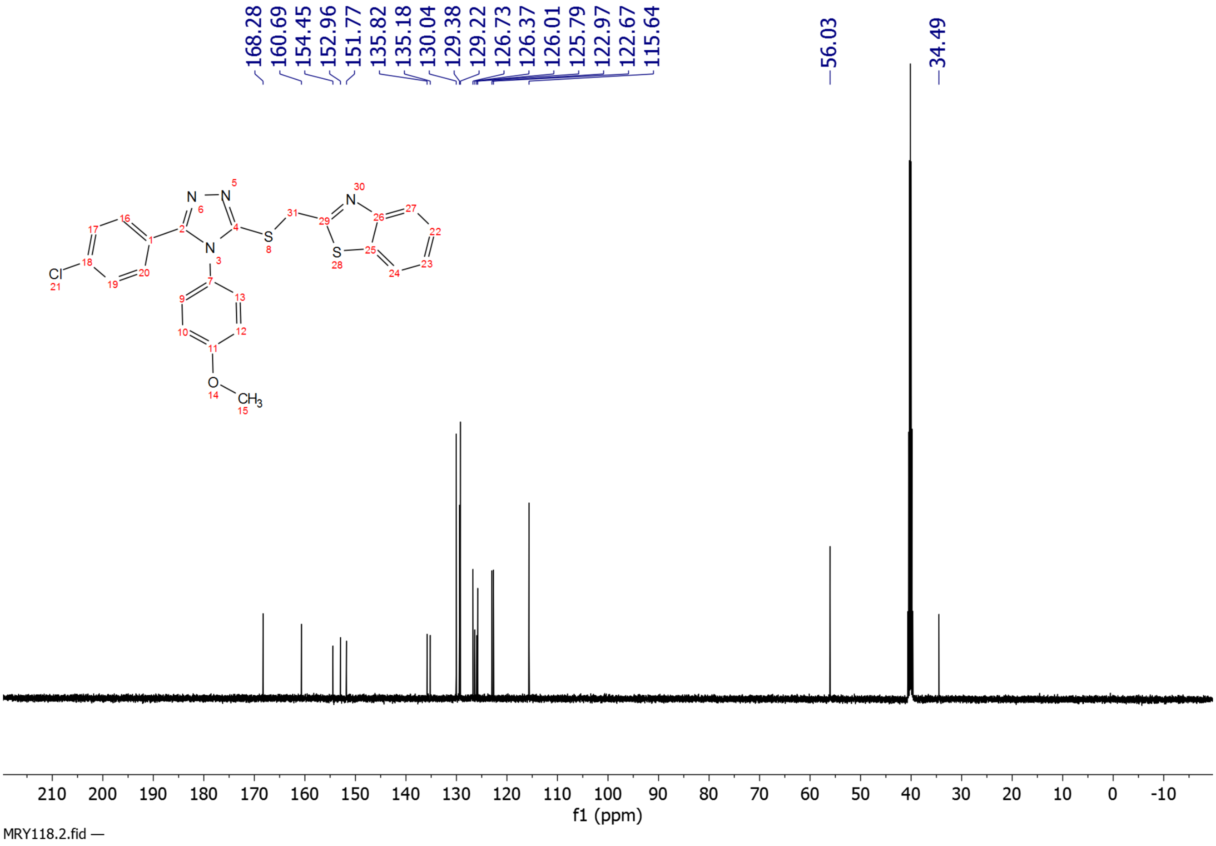

Figure S41. ^{1}H-NMR Spectrum of 6s

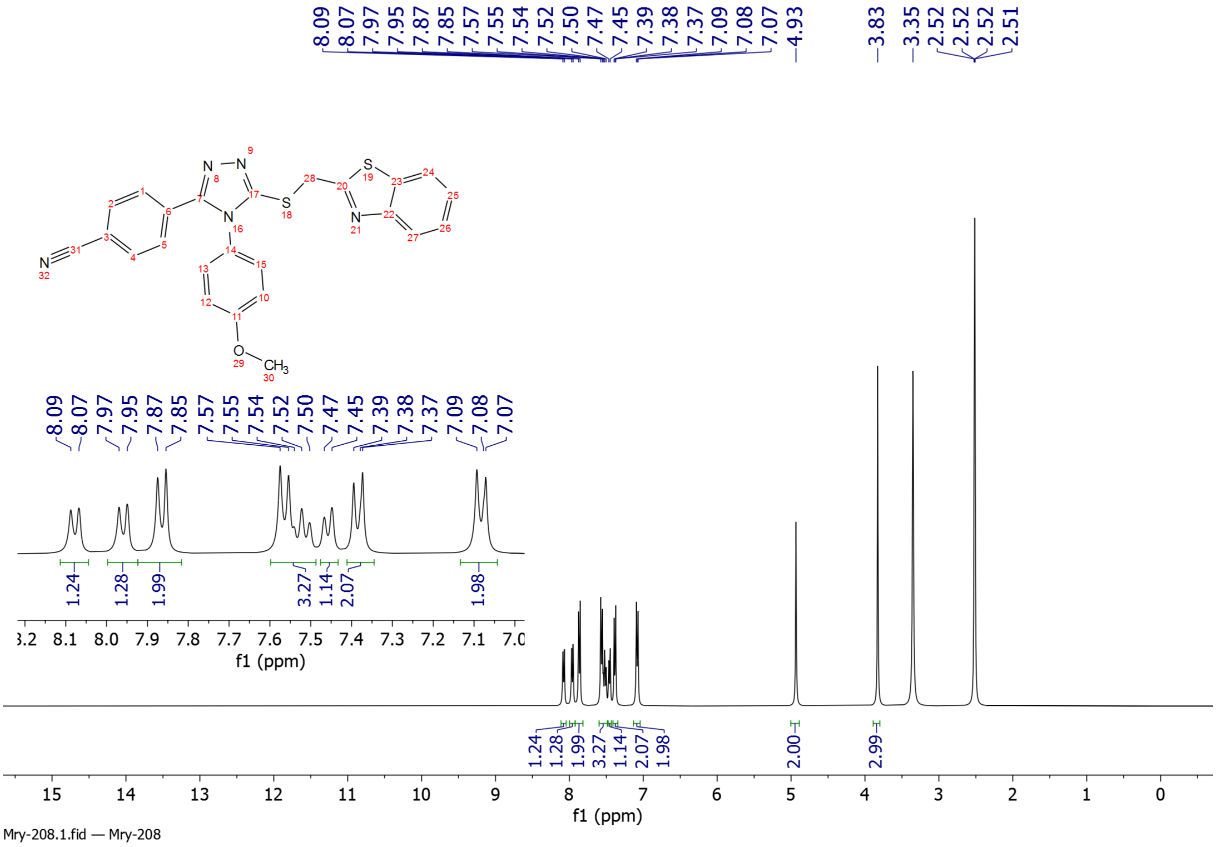

Figure S42. $^{13}C_{APT}$-NMR Spectrum of 6s

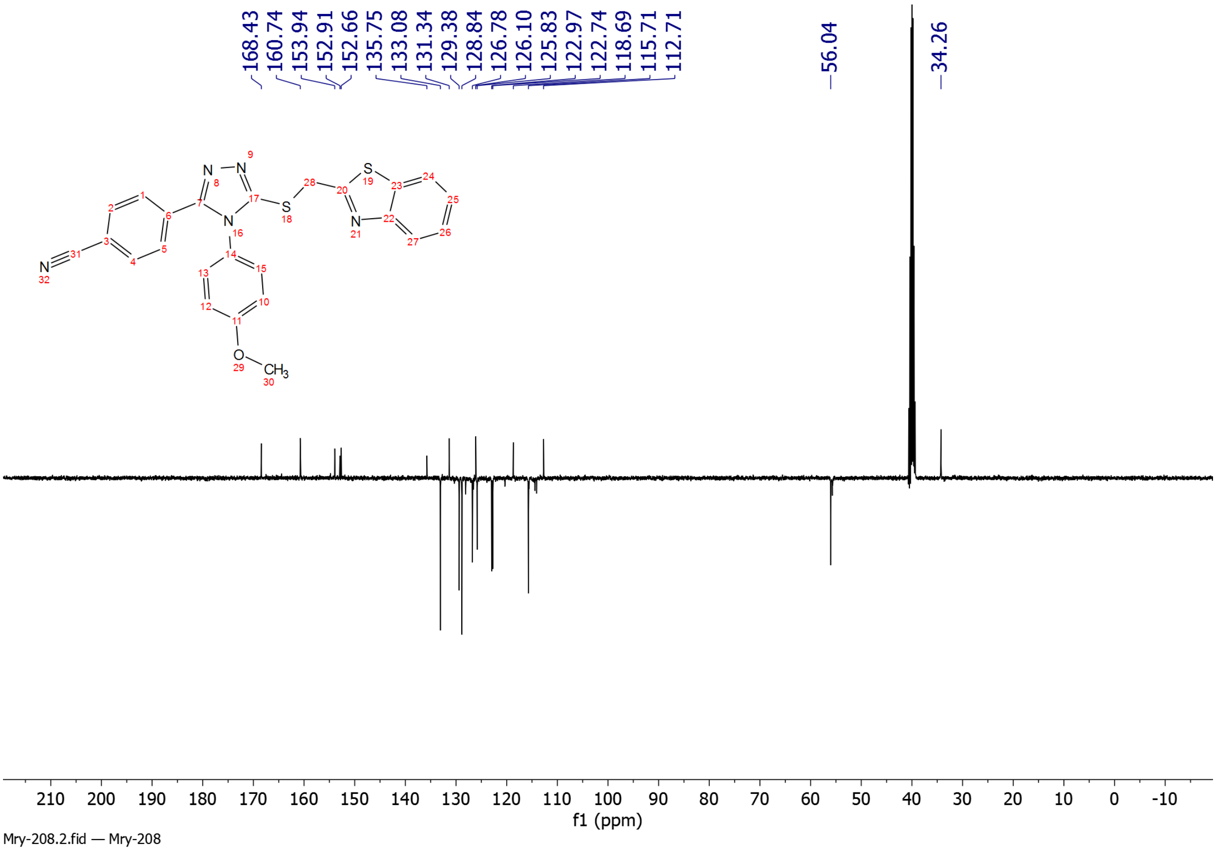

Figure S43. ^{1}H-NMR Spectrum of 6t

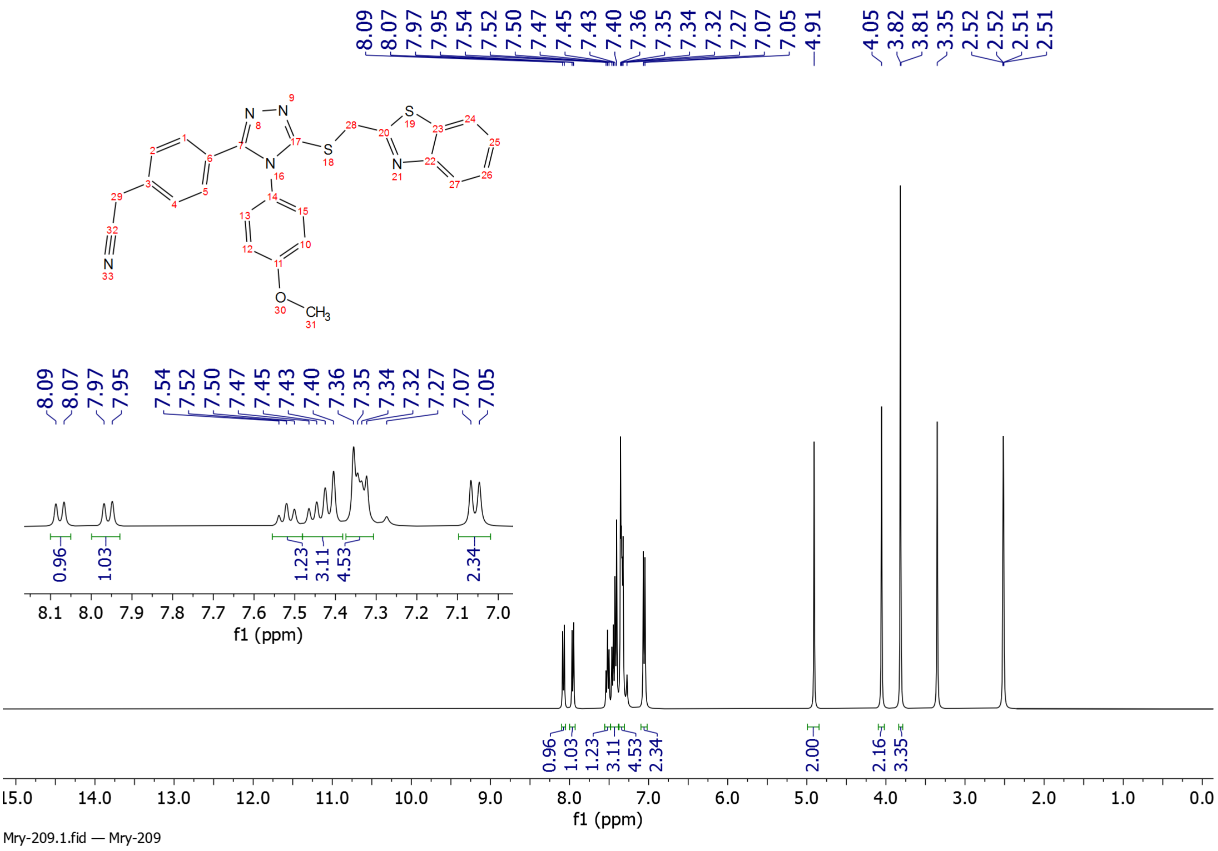

Figure S44. $^{13}C_{APT}$-NMR Spectrum of 6t

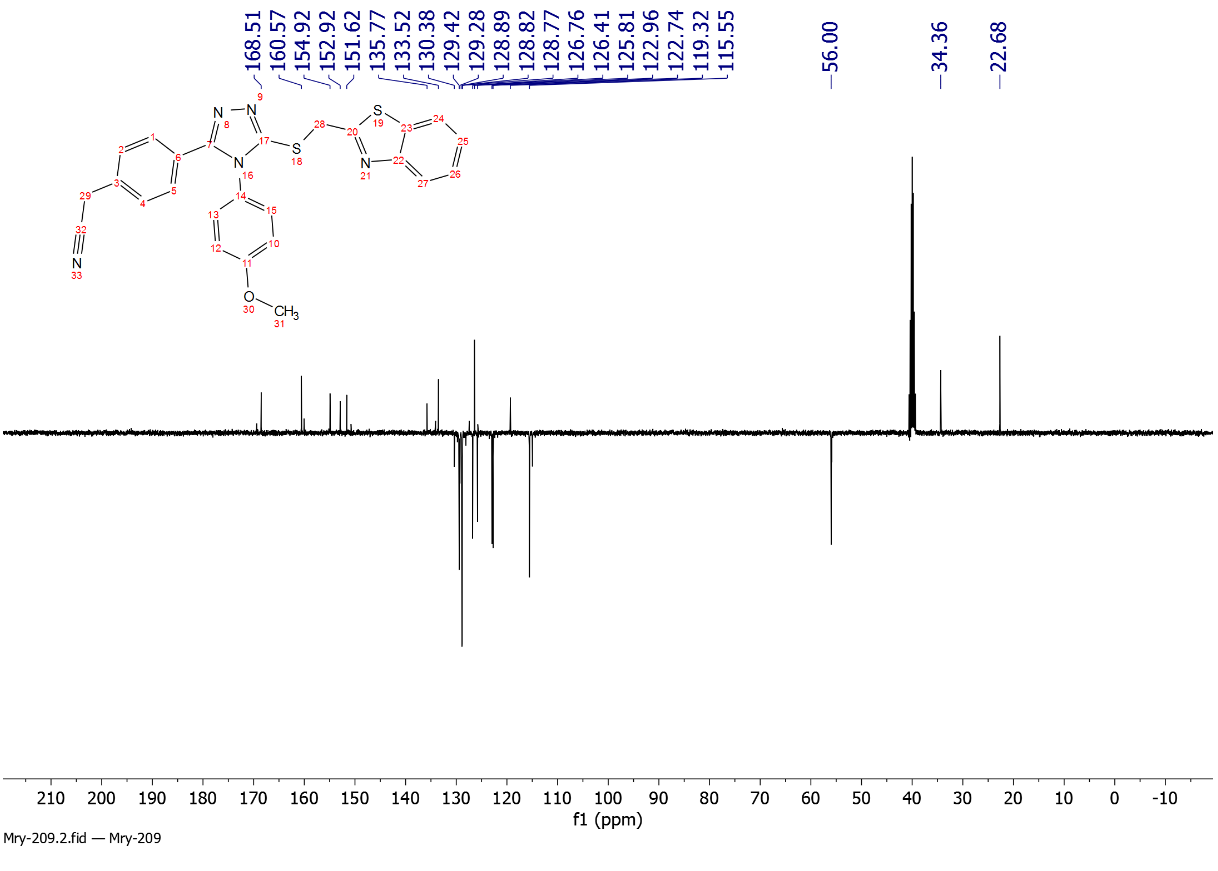

Figure S45. ^{1}H-NMR Spectrum of 6u

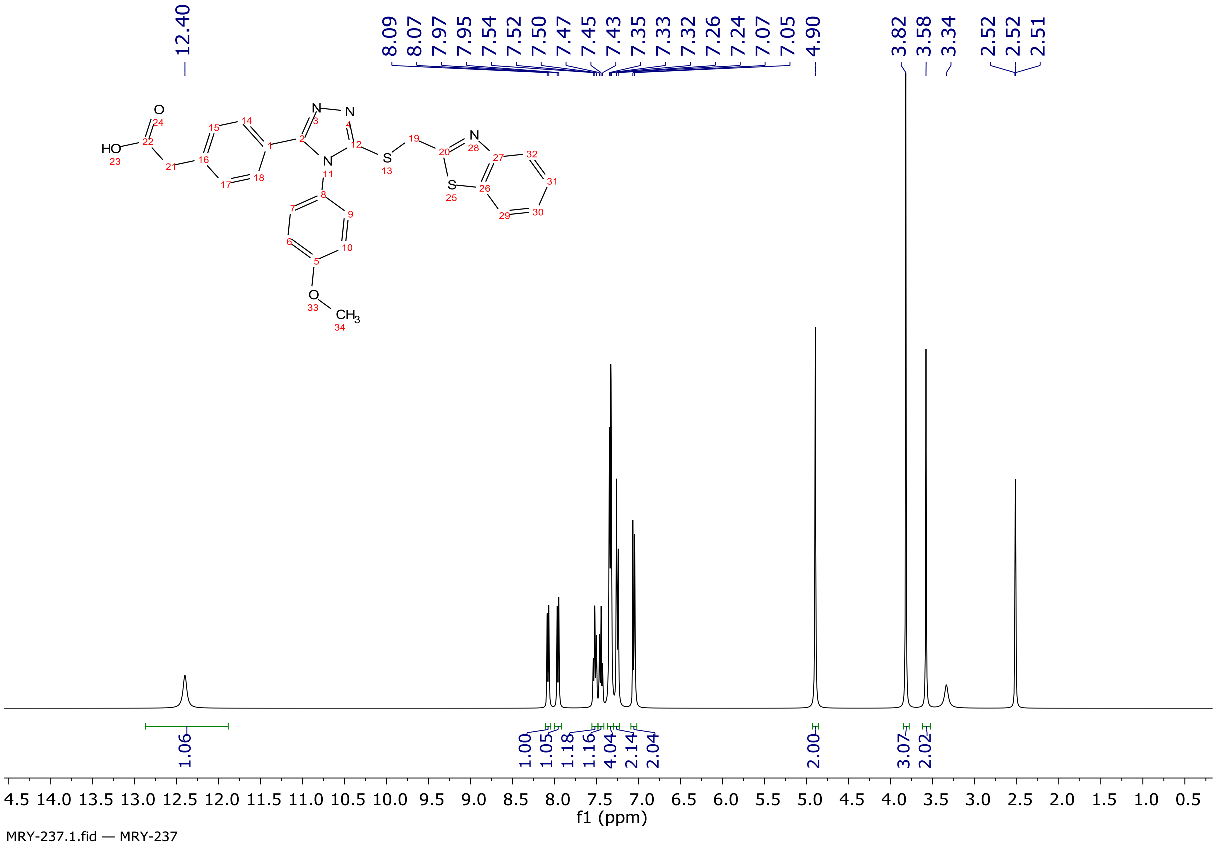

Figure S46. ^{13}C$_{APT}$-NMR Spectrum of 6u

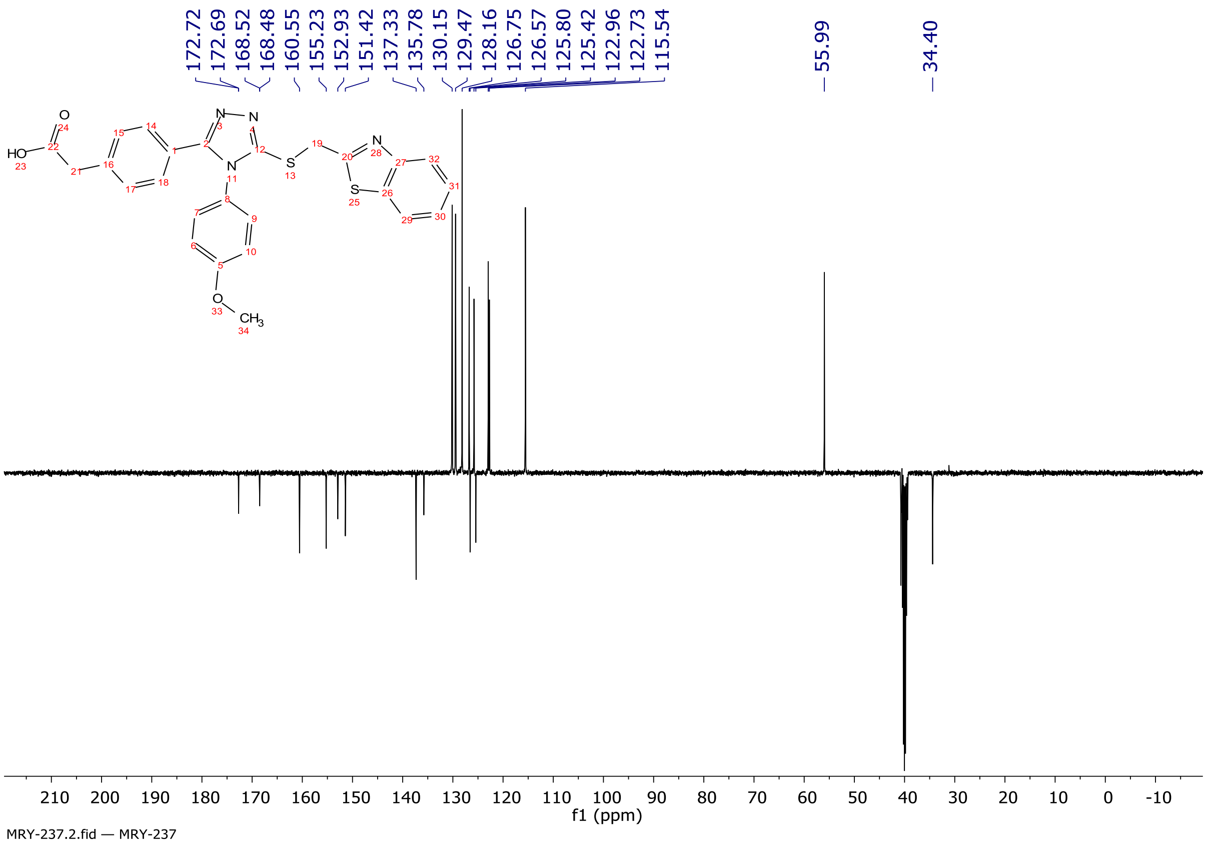

Figure S47. ^{1}H-NMR Spectrum of 6v

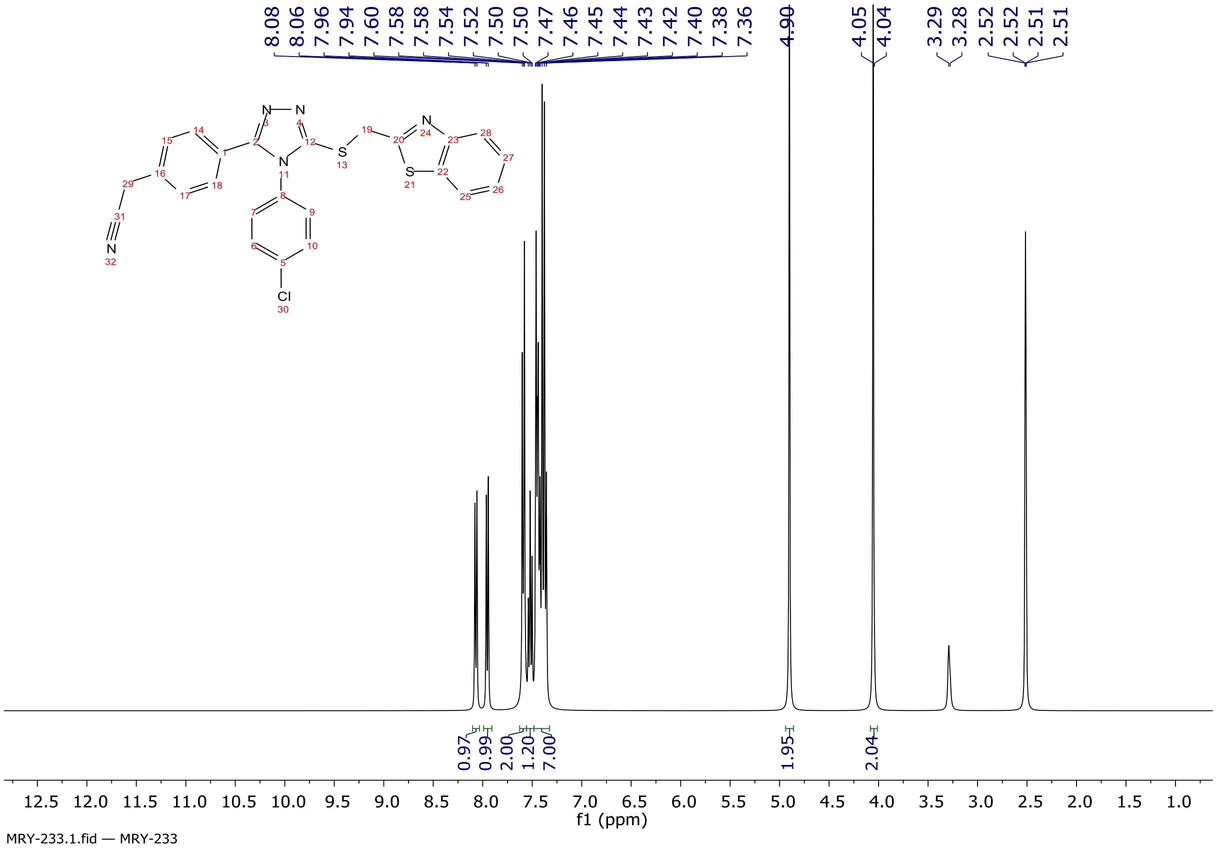

Figure S48. $^{13}C_{APT}$-NMR Spectrum of 6v

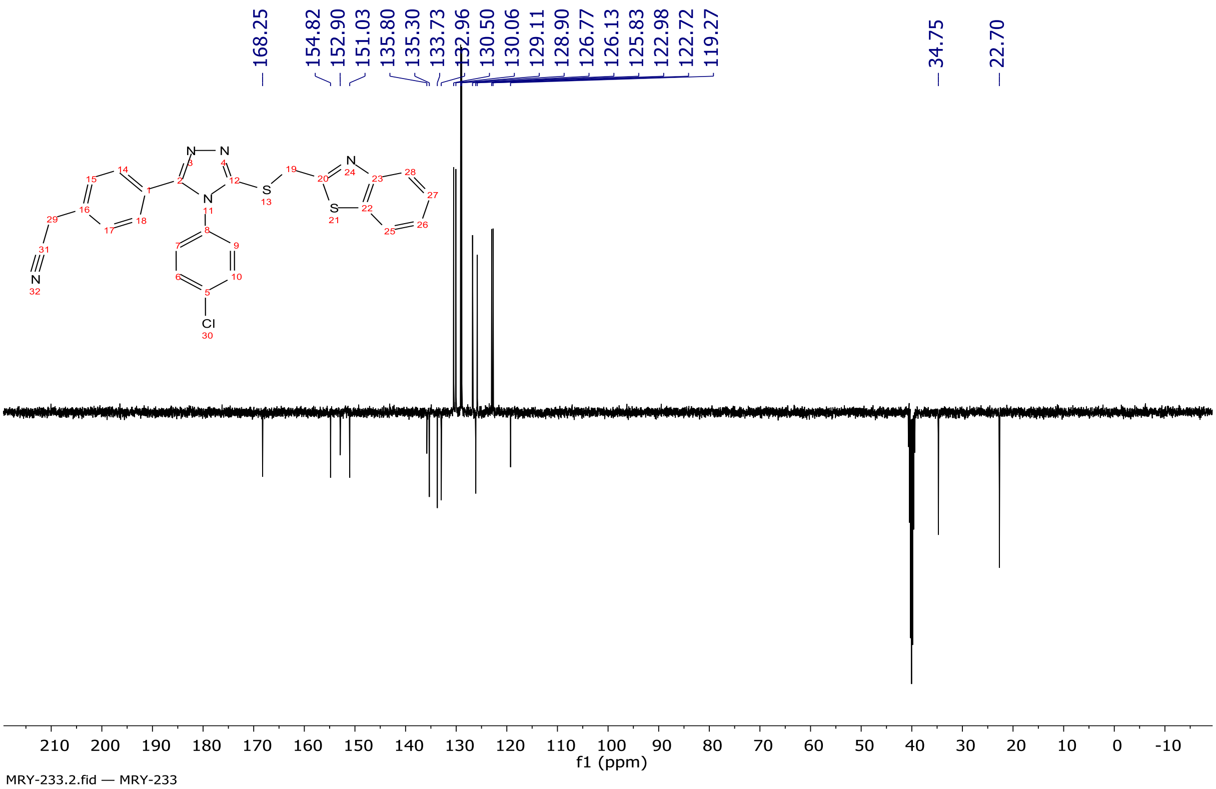

Figure S49. ^{1}H-NMR Spectrum of 6w

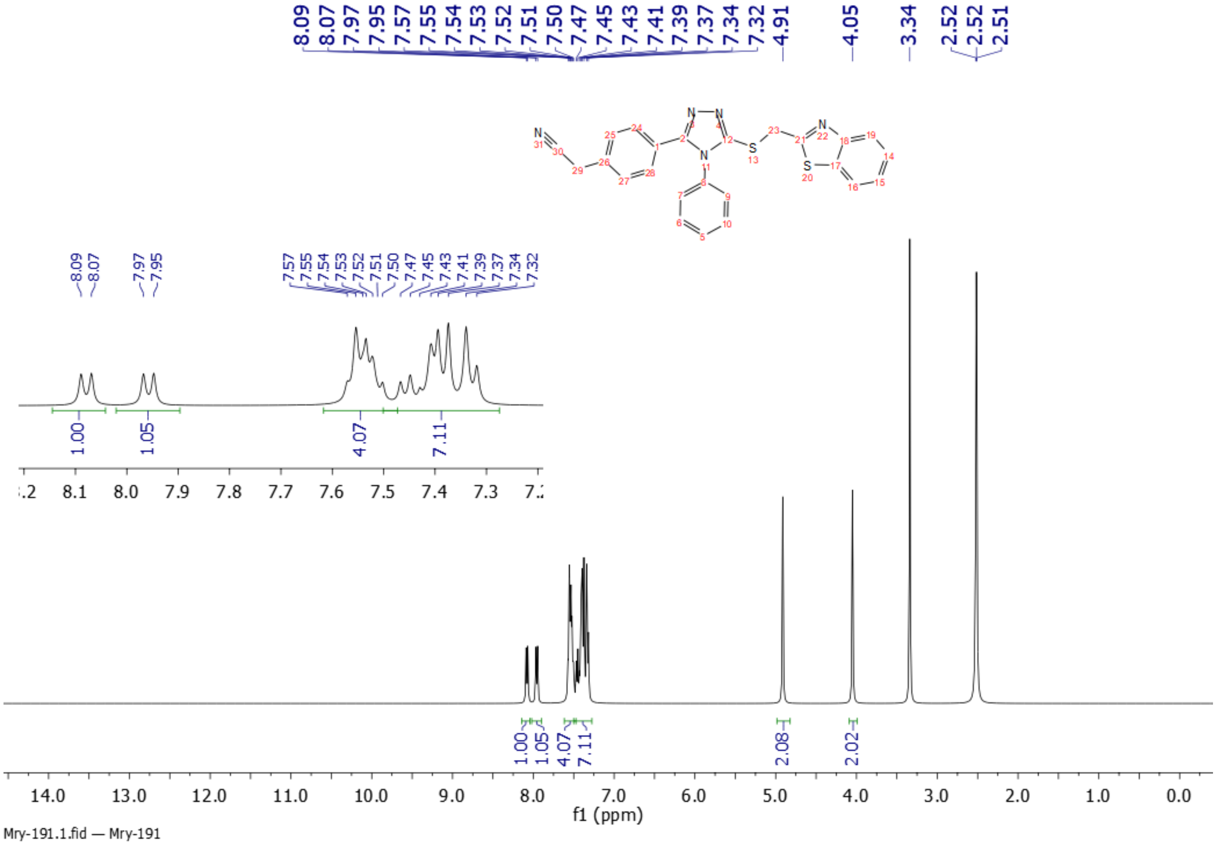

Figure S50. $^{13}C_{APT}$-NMR Spectrum of 6w

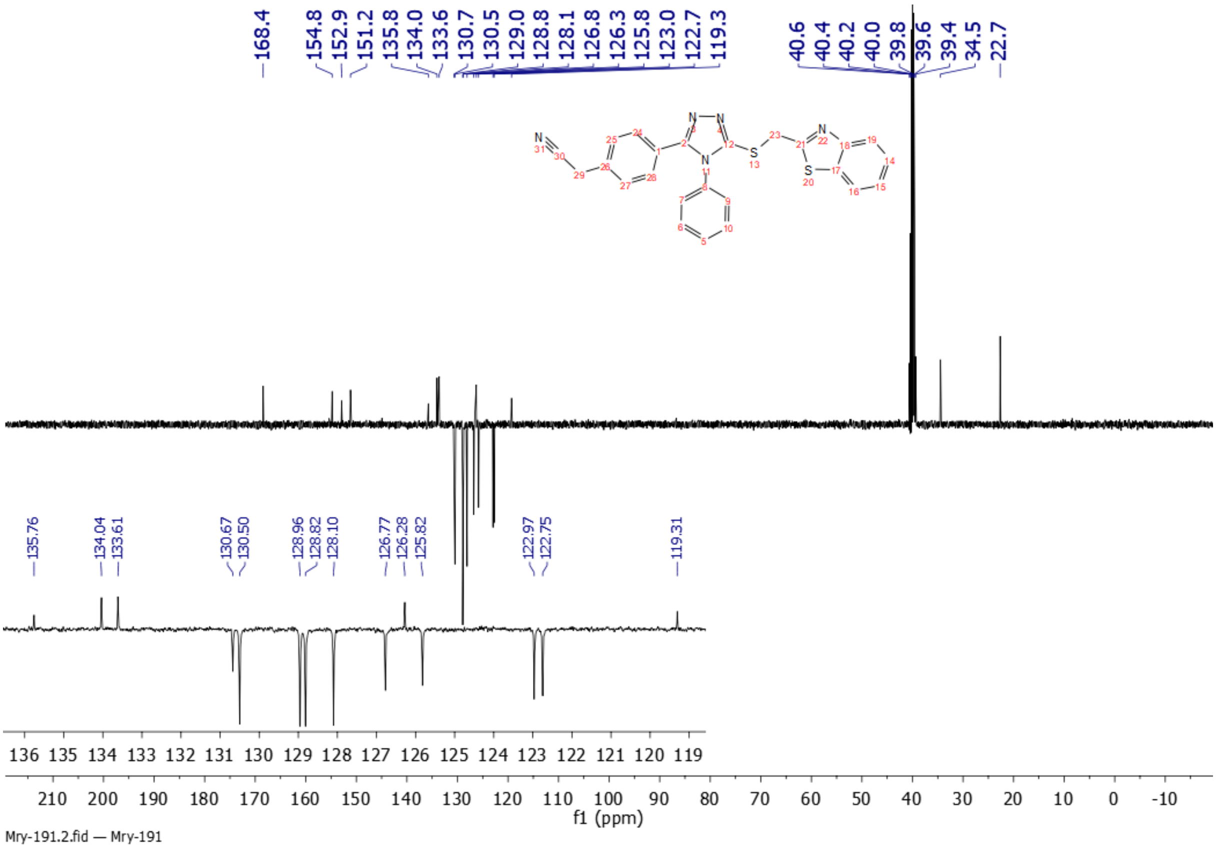

Figure S51. ^{1}H-NMR Spectrum of 6x

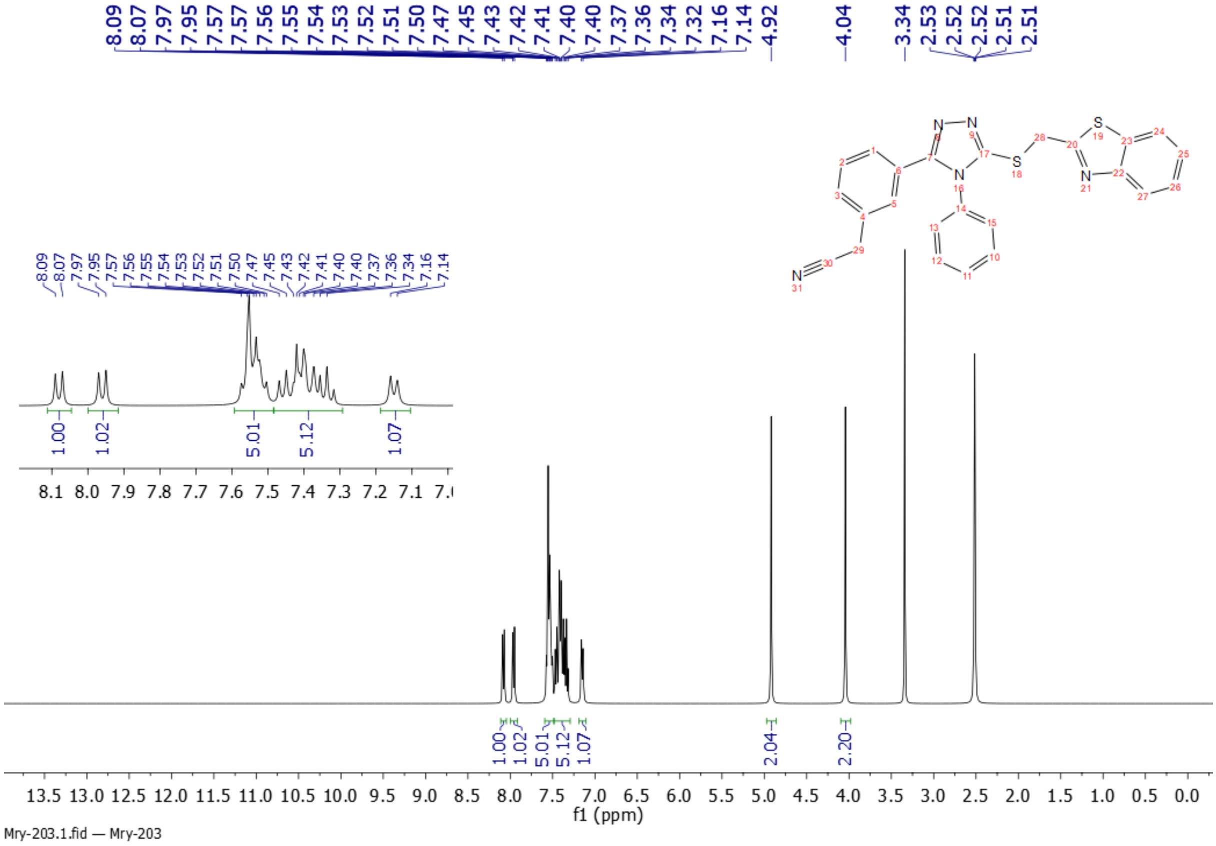

Figure S52. $^{13}C_{APT}$-NMR Spectrum of 6x

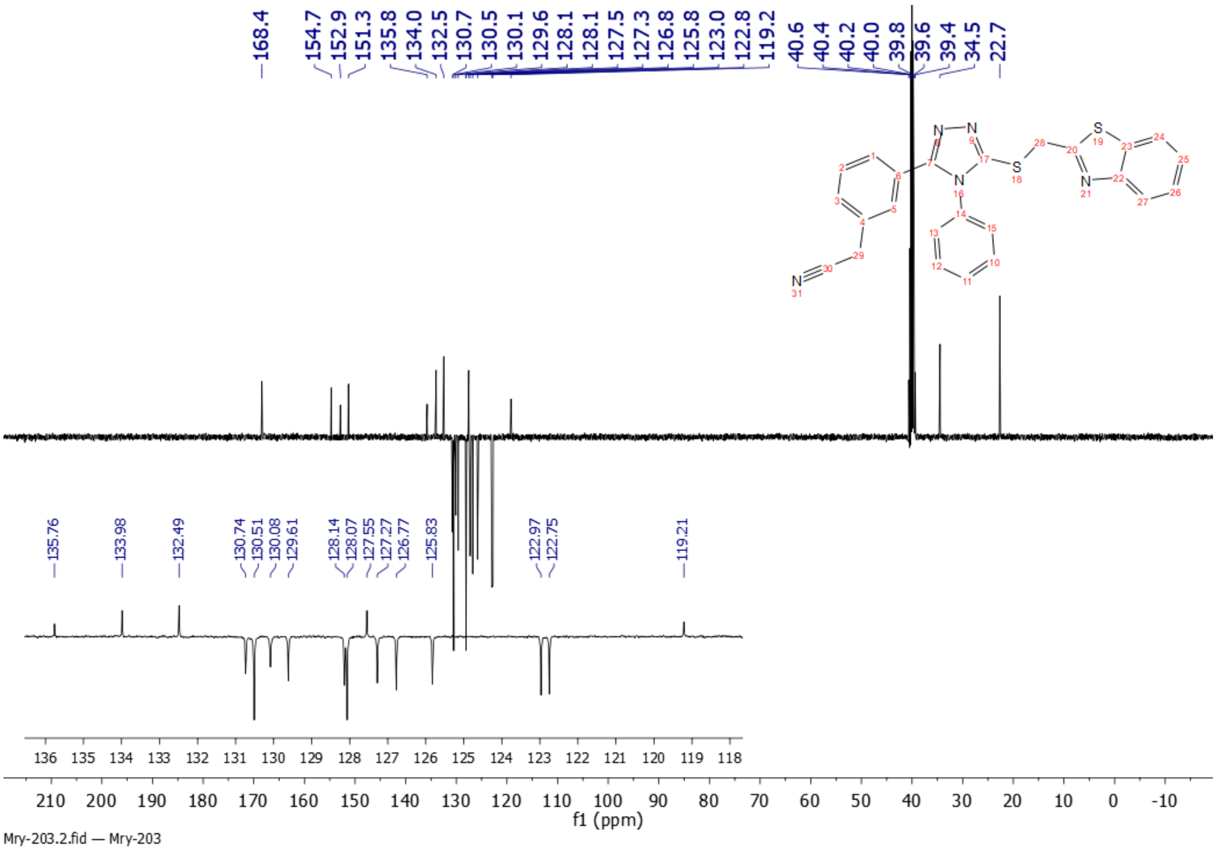

Figure S53. ^{1}H-NMR Spectrum of 6y

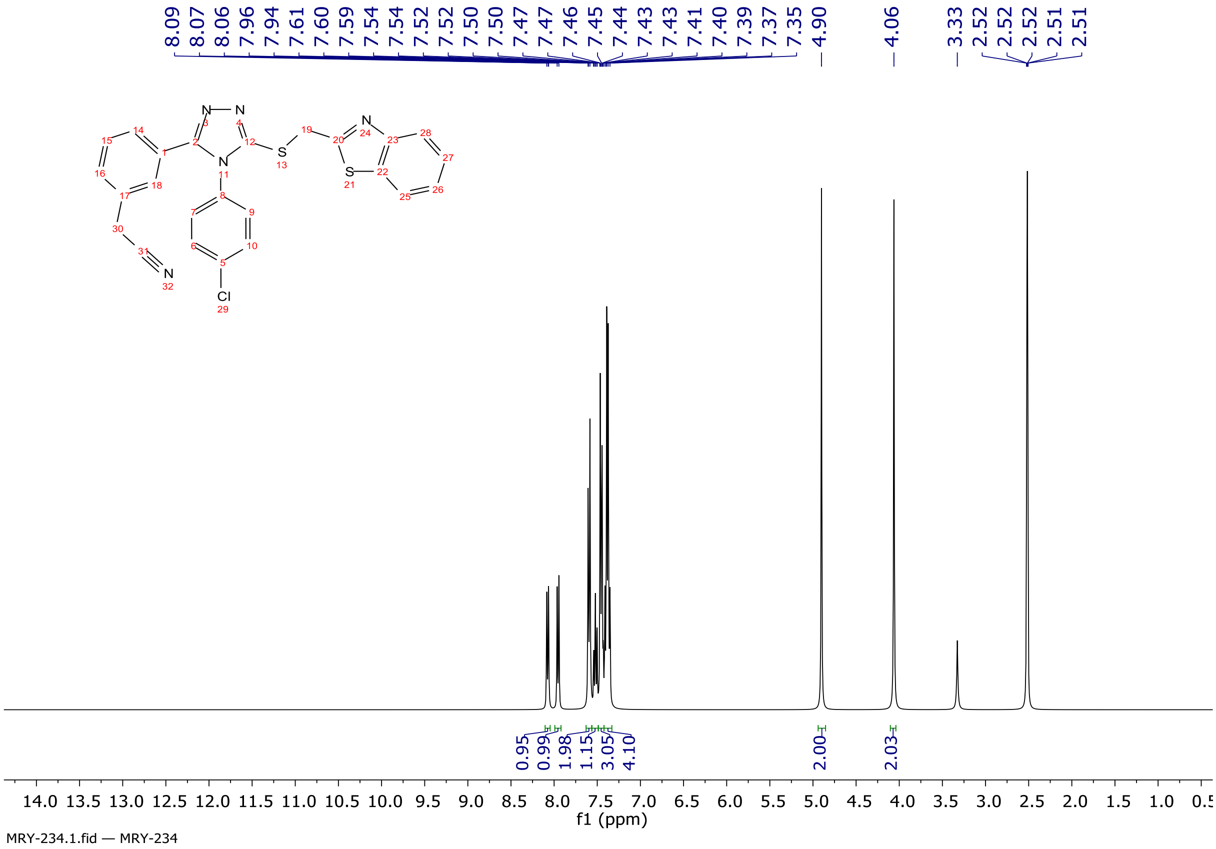

Figure S54. ^{13}C$_{APT}$-NMR Spectrum of 6y

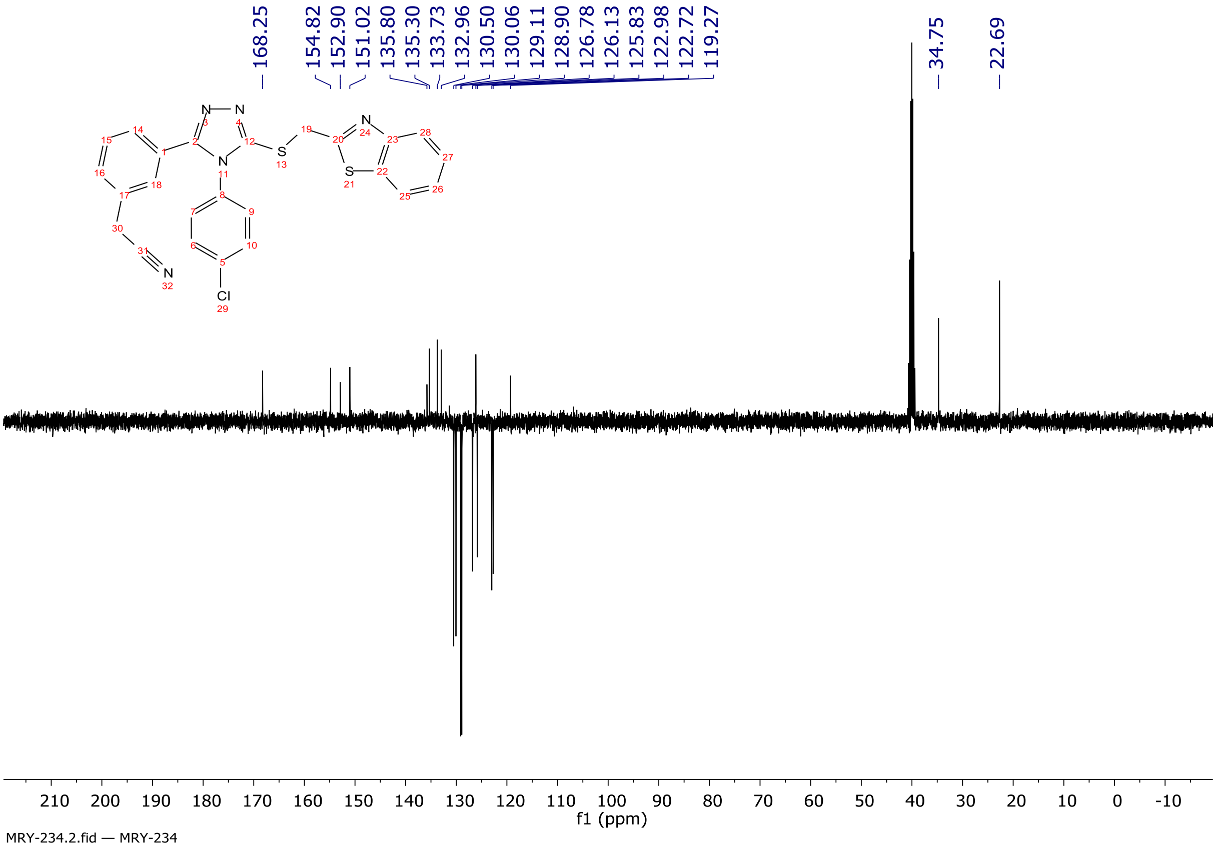

Figure S55. ^{1}H-NMR Spectrum of 6z

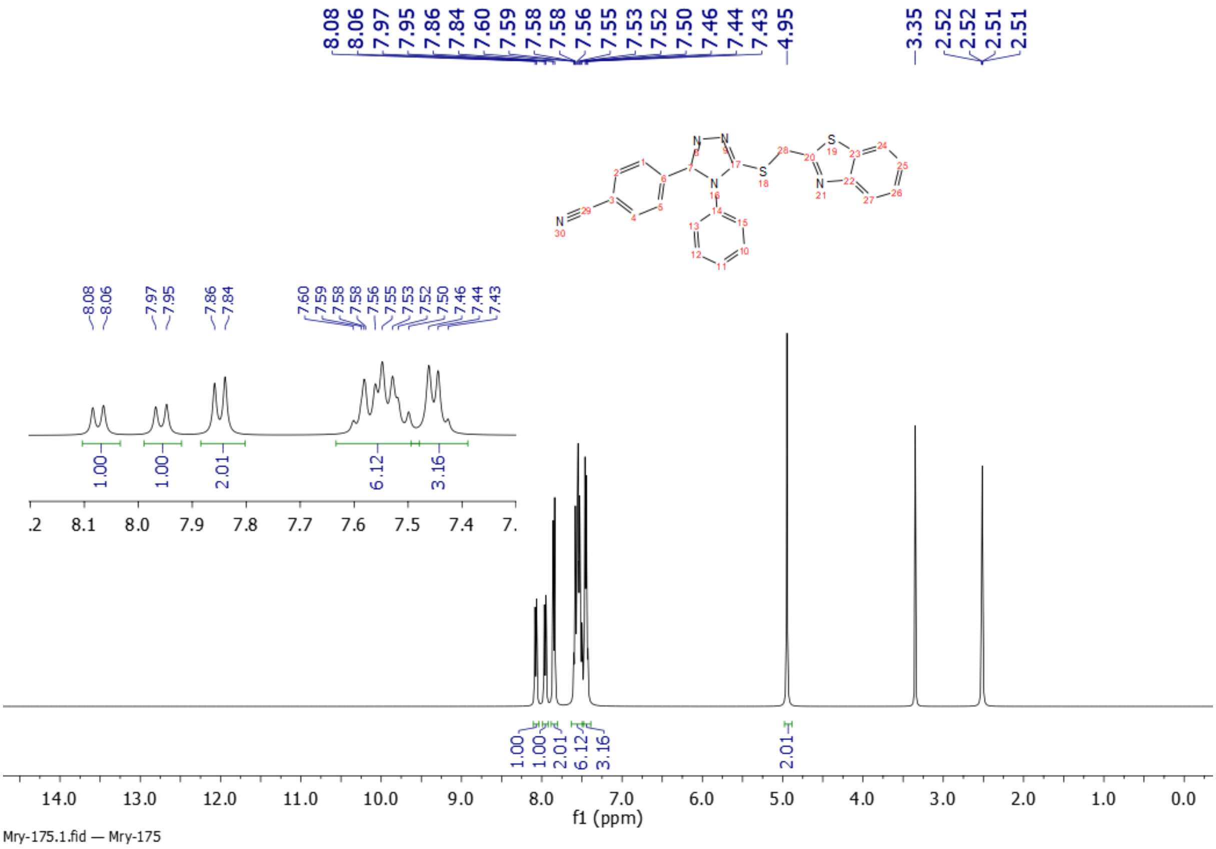

Figure S56. $^{13}C_{APT}$-NMR Spectrum of 6z

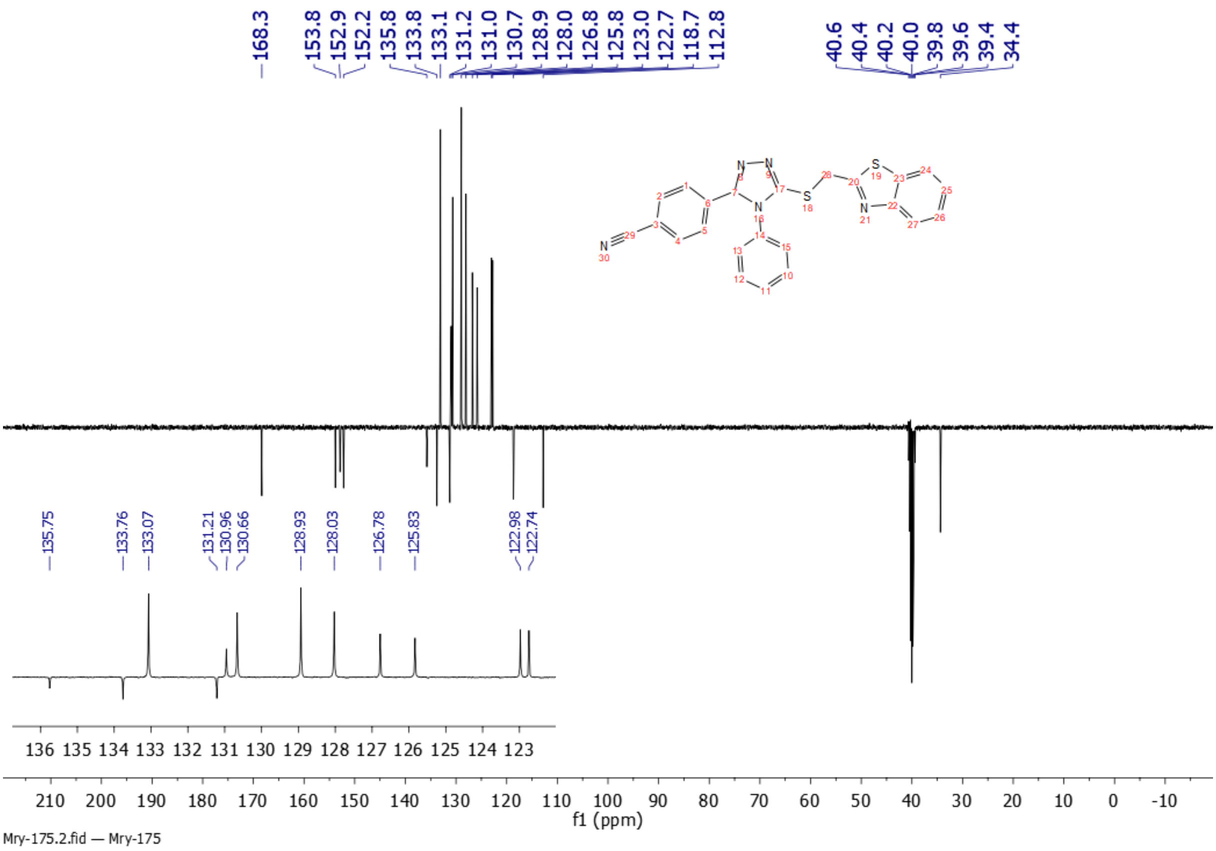

Figure S57. HRMS Spectrum of 6a

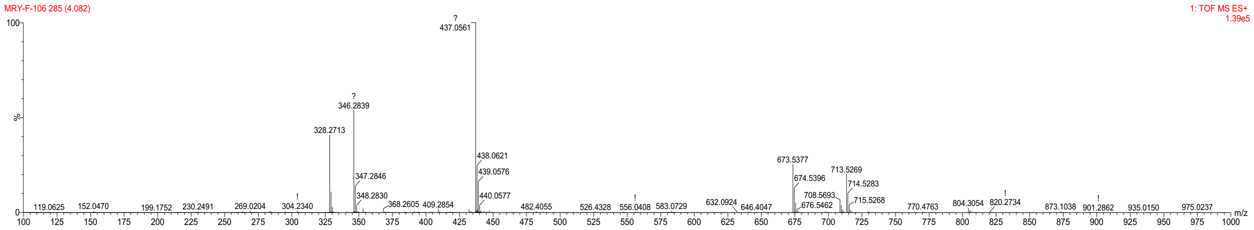

Figure S58. HRMS Spectrum of 6b

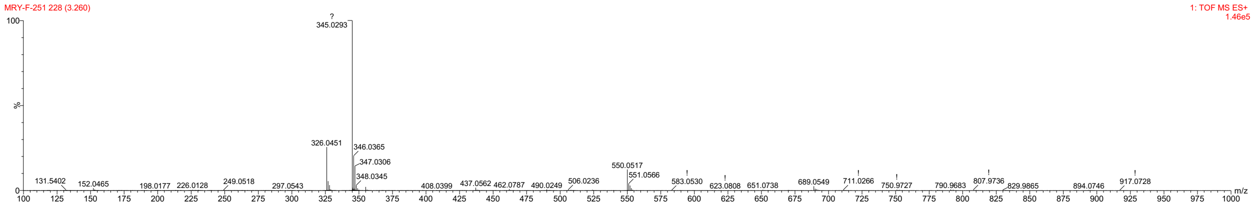

Figure S59. HRMS Spectrum of 6c

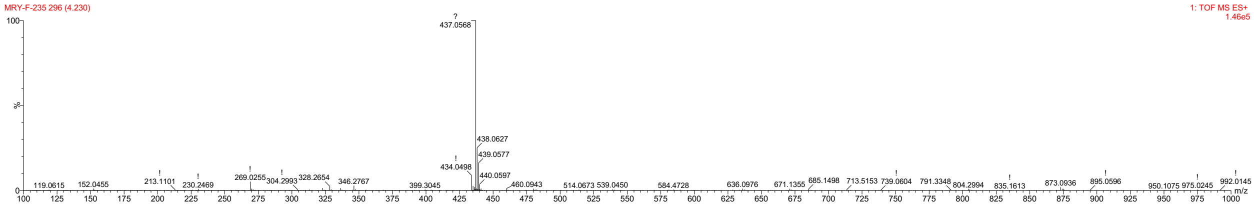

Figure S60. HRMS Spectrum of 6d

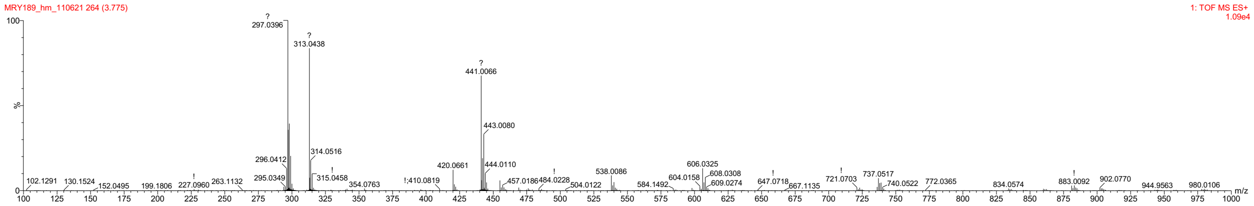

Figure S61. HRMS Spectrum of 6e

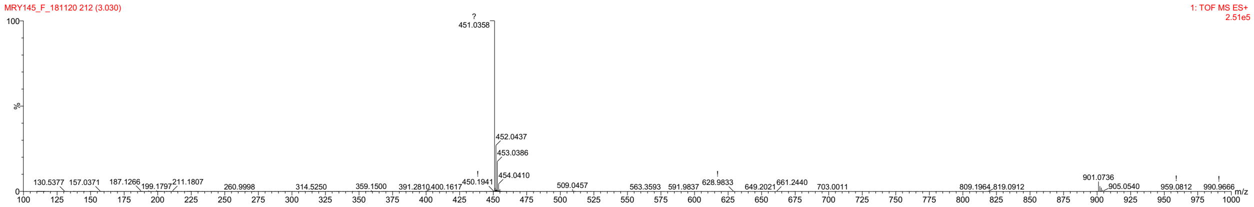

Figure S62. HRMS Spectrum of 6f

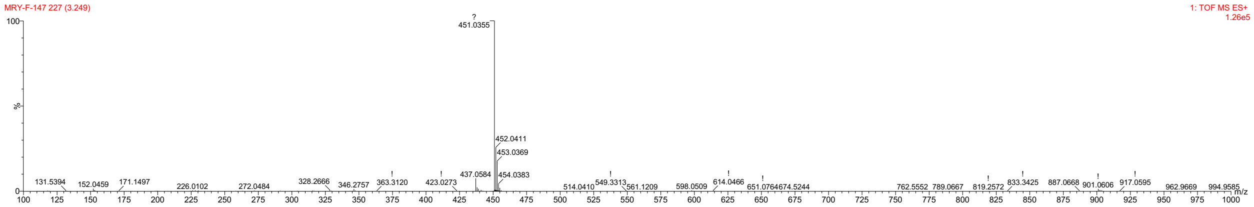

Figure S63. HRMS Spectrum of 6g

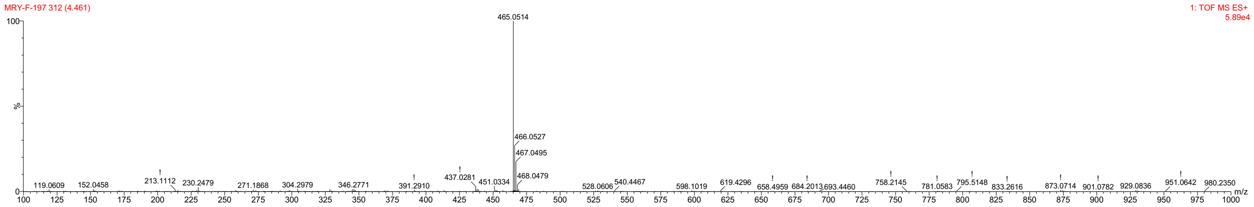

Figure S64. HRMS Spectrum of 6h

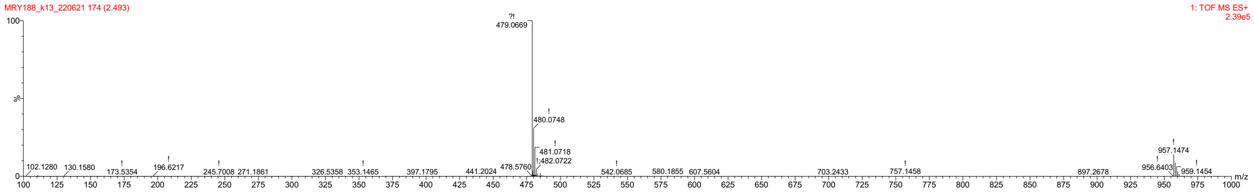

Figure S65. HRMS Spectrum of 6i

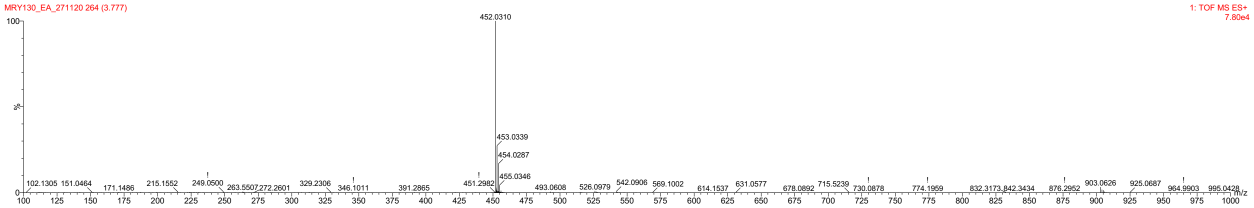

Figure S66. HRMS Spectrum of 6j

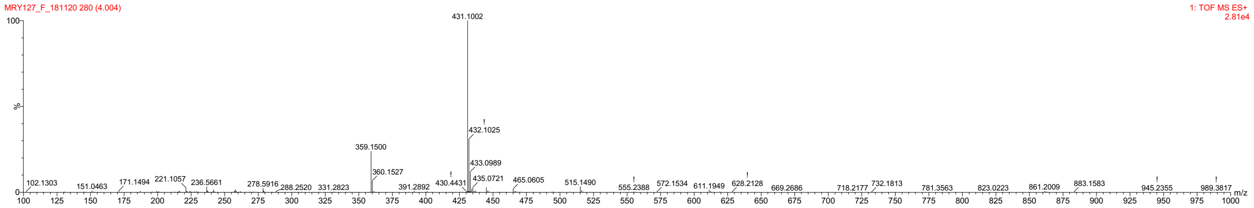

Figure S67. HRMS Spectrum of 6k

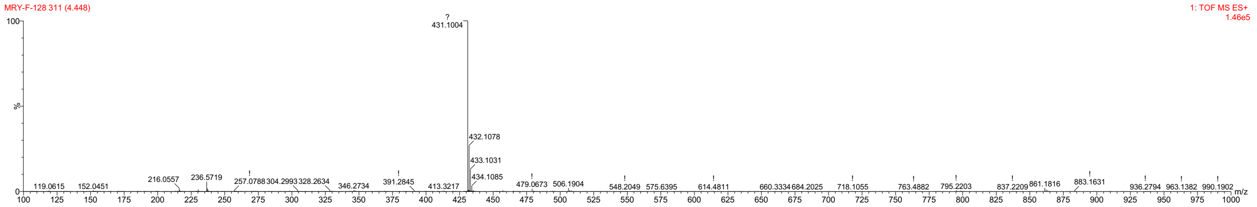

Figure S68. HRMS Spectrum of 6l

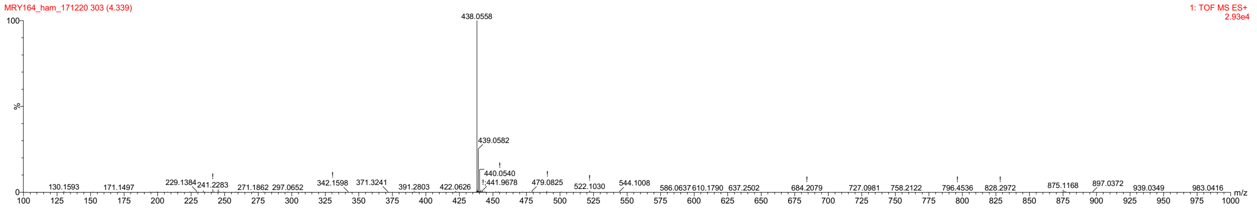

Figure S69. HRMS Spectrum of 6m

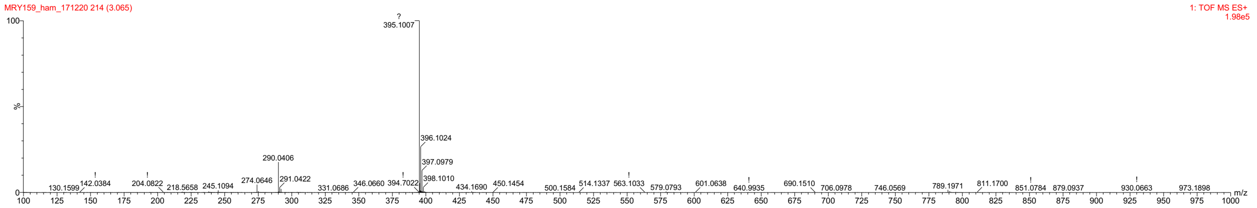

Figure S70. HRMS Spectrum of 6n

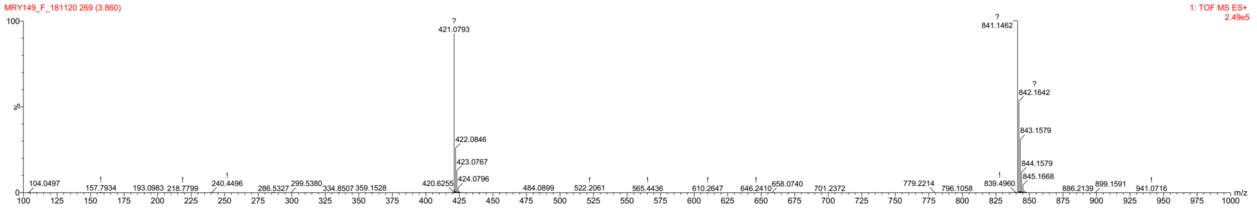

Figure S71. HRMS Spectrum of 6o

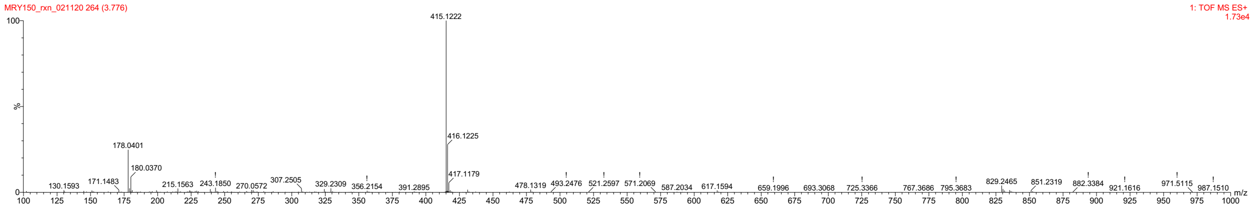

Figure S72. HRMS Spectrum of 6p

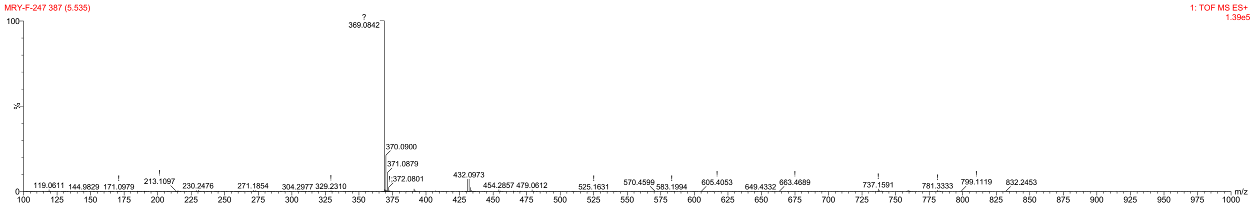

Figure S73. HRMS Spectrum of 6q

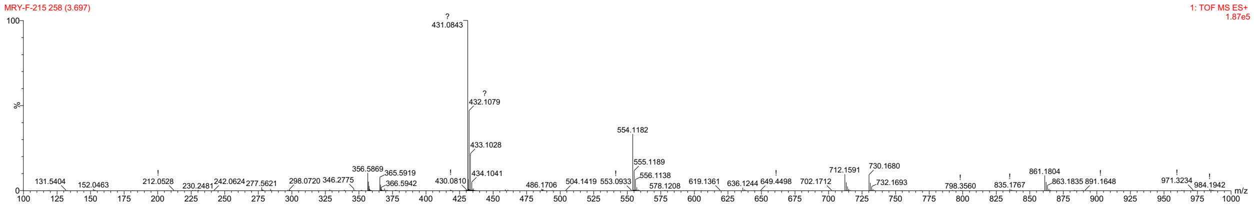

Figure S74. HRMS Spectrum of 6r

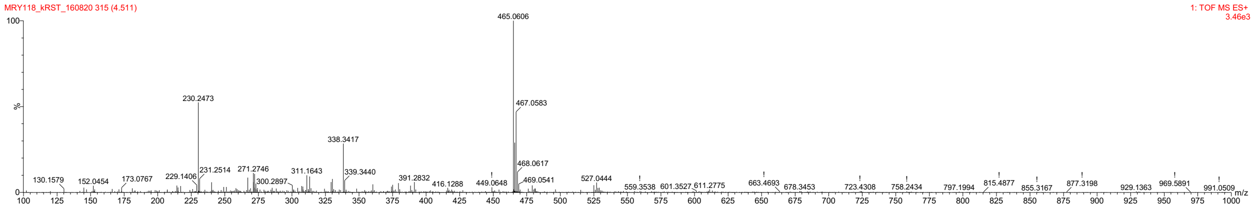

Figure S75. HRMS Spectrum of 6s

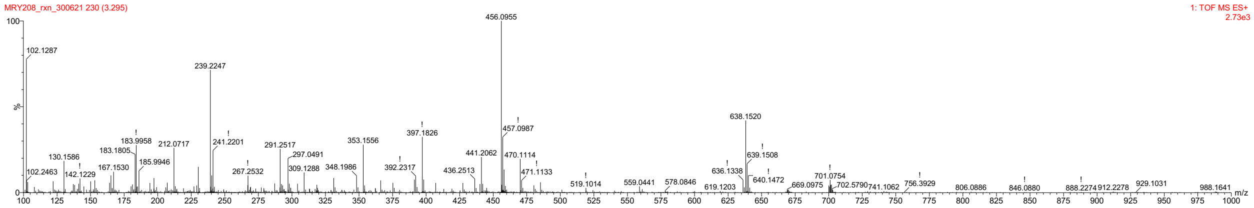

Figure S76. HRMS Spectrum of 6t

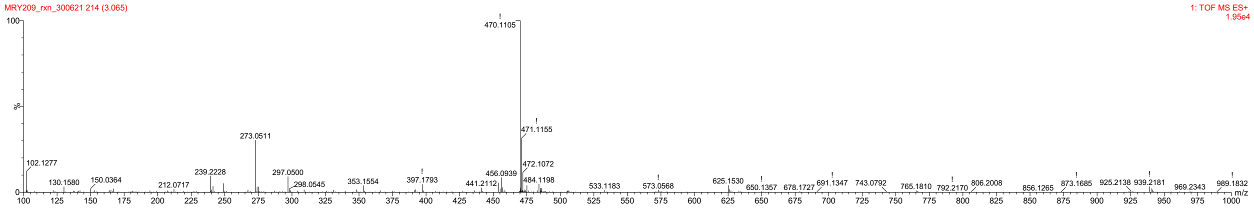

Figure S77. HRMS Spectrum of 6u

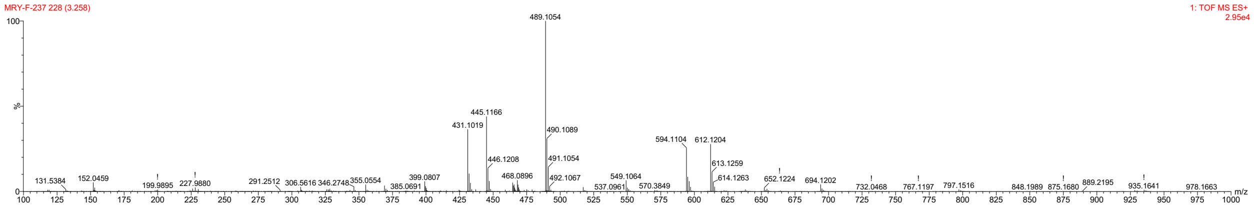

Figure S78. HRMS Spectrum of 6v

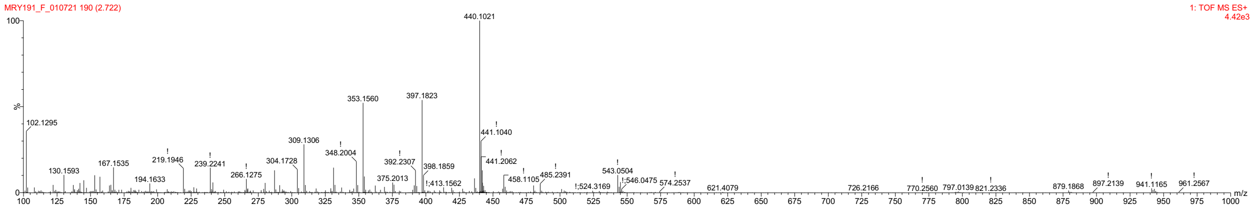

Figure S79. HRMS Spectrum of 6w

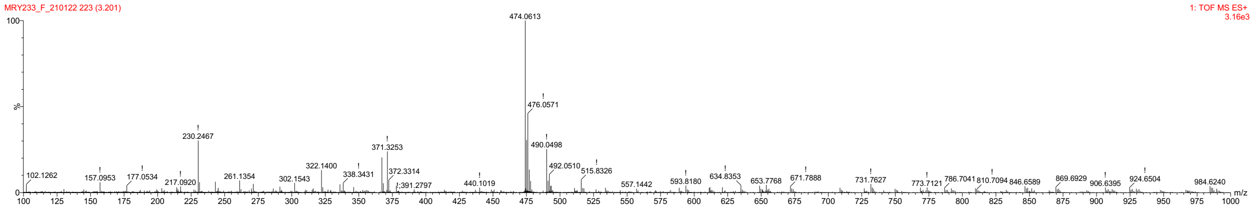

Figure S80. HRMS Spectrum of 6x

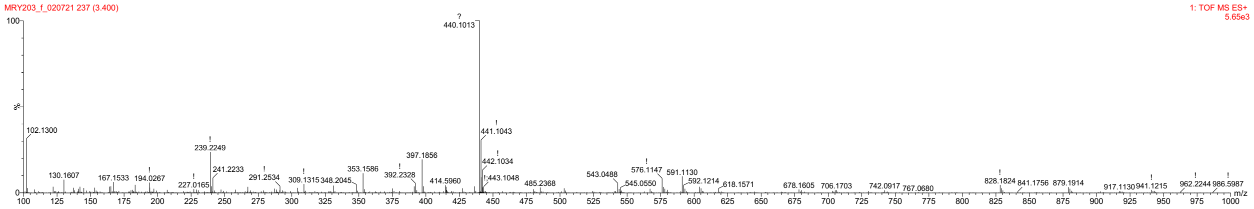

Figure S81. HRMS Spectrum of 6y

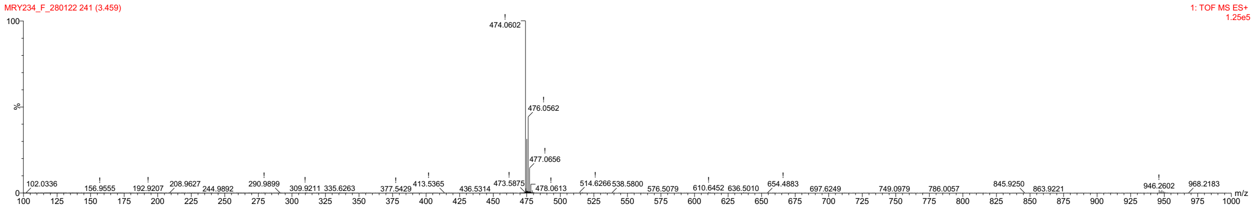

Figure S82. HRMS Spectrum of 6z

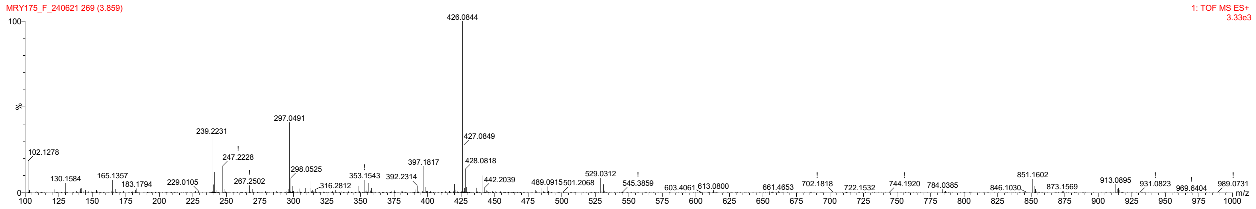

Figure S83. IC_{50} Curves of Compounds 6a, 6c, 6d, 6j-l, 6n, 6o, 6q, 6s, 6t, 6v-z

Compounds were tested in human neutrophils stimulated with 2.5 µM A23187. Data are given as percentage of control at the given inhibitor concentrations (means ± SEM, n = 3).

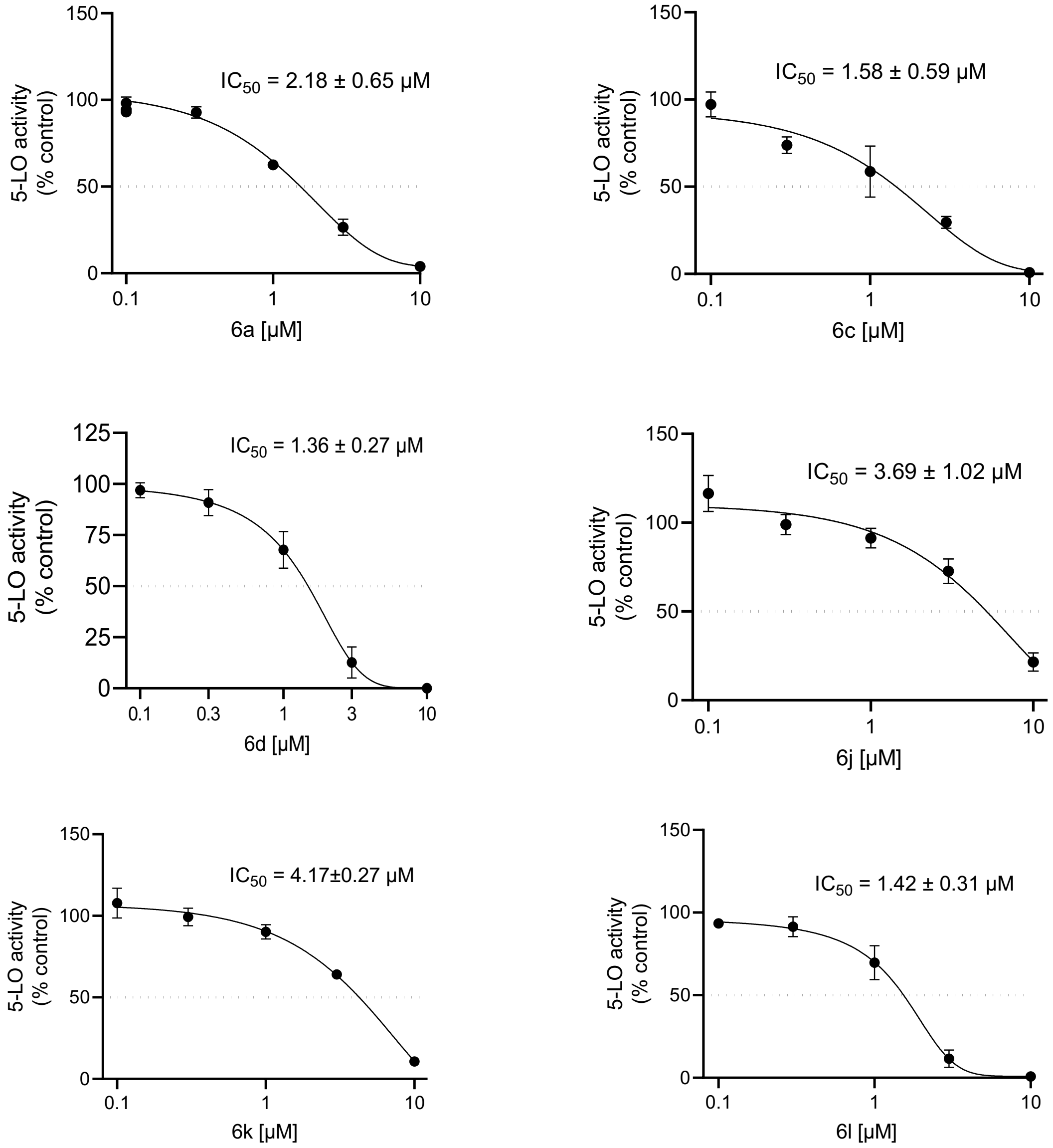

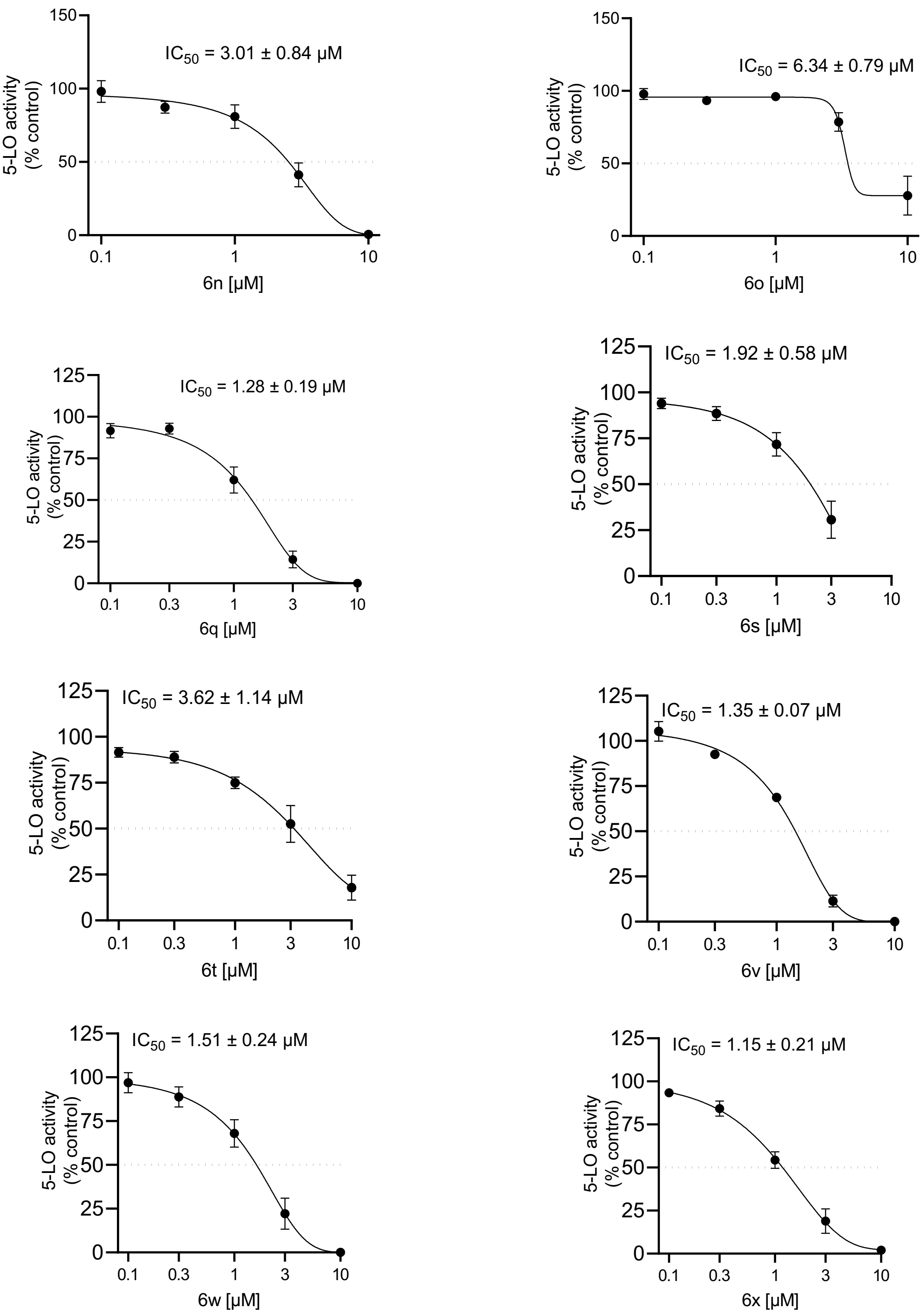

IC50 = 3.01 ± 0.84 µM
5-LO activity (% control)
150
100
50
0
0.1
1
10
6n [µM]
IC50 = 6.34 ± 0.79 µM
5-LO activity (% control)
150
100
50
0
0.1
1
10
6o [µM]
IC50 = 1.28 ± 0.19 µM
5-LO activity (% control)
125
100
75
50
25
0
0.1
0.3
1
3
10
6q [µM]
IC50 = 1.92 ± 0.58 µM
5-LO activity (% control)
125
100
75
50
25
0
0.1
0.3
1
3
10
6s [µM]
IC50 = 3.62 ± 1.14 µM
5-LO activity (% control)
125
100
75
50
25
0
0.1
0.3
1
3
10
6t [µM]
IC50 = 1.35 ± 0.07 µM
5-LO activity (% control)
125
100
75
50
25
0
0.1
0.3
1
3
10
6v [µM]
IC50 = 1.51 ± 0.24 µM
5-LO activity (% control)
125
100
75
50
25
0
0.1
0.3
1
3
10
6w [µM]
IC50 = 1.15 ± 0.21 µM
5-LO activity (% control)
125
100
75
50
25
0
0.1
0.3
1
3
10
6x [µM]

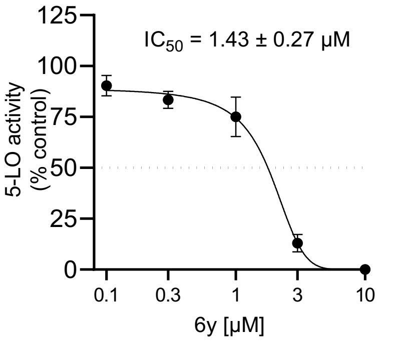

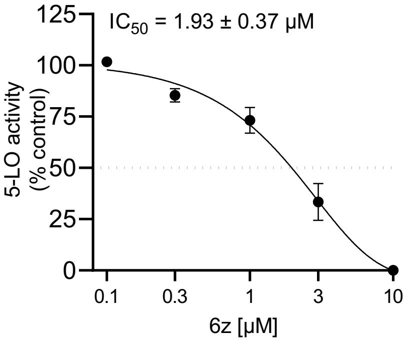

References

[1] The PyMOL Molecular Graphics System, Version 2.5 Schrödinger, LLC.
